# Supplementary material for: Interplay of structure and photophysics of individualized rod-shaped graphene quantum dots with up to 132 sp² carbon atoms
Source: Nat Commun. 2023 Aug 7;14:4728. doi: 10.1038/s41467-023-40376-w (PMC10406913; doi:10.1038/s41467-023-40376-w)
Supplement: Supplementary file 1 — Supplementary Information [file 41467_2023_40376_MOESM1_ESM.pdf]

Supplementary Information

Interplay of structure and photophysics of individualized rod-shaped graphene quantum dots with up to 132 *sp*² carbon atoms

Daniel Medina-Lopez,[1‡] Thomas Liu,[2‡] Silvio Osella,[3] Hugo Levy-Falk,[2] Nicolas Rolland,[4] Christine Elias,[2] Gaspard Huber,[5] Pranav Ticku,[2] Loïc Rondin,[2] Bruno Jousselme,[1] David Beljonne,[4] Jean-Sébastien Lauret[2,*] and Stephane Campidelli[1,*]

[1]Université Paris-Saclay, CEA, CNRS, NIMBE, LICSEN, 91191, Gif-sur-Yvette, France.

[2]Université Paris-Saclay, ENS Paris-Saclay, Centrale Supélec, CNRS, LuMIn, 91400, Orsay, France.

[3]Chemical and Biological Systems Simulation Lab, Centre of New Technologies, University of Warsaw, Banacha 2C, 02-097 Warsaw, Poland.

[4]Laboratory for Chemistry of Novel Materials, University of Mons, 7000 Mons, Belgium.

[5]Université Paris-Saclay, CEA, CNRS, NIMBE, LSDRM, 91191, Gif-sur-Yvette, France.

[‡]These authors contributed equally

Table of Contents

Supplementary methods 4

Supplementary figures and supplementary tables 16

Supplementary Figure 1. Structure and optical characterization of the triangular-shaped C_{96}-*t*Bu_6 16

Supplementary Figure 2. Purification and Mass Analysis of C_{78}-*t*Bu_6, C_{96}-*t*Bu_8, C_{114}-*t*Bu_{10} and C_{132}-*t*Bu_{12} GQDs. 17

Supplementary Figure 3. Cyclic Voltammetry of GQDs. 20

Supplementary Figure 4. Comparison of absorption, PL and PLE of C_{96}-*t*Bu_8 and C_{96}-$(C_{12}H_{25})_6$ GQD. 21

Supplementary Figure 5. Normalized absorption spectra of C_{78}-*t*Bu_6, C_{96}-*t*Bu_8, C_{114}-*t*Bu_{10} and C_{132}-*t*Bu_{12}. 22

Supplementary Figure 6. Calculated absorption spectra of C_{78}-*t*Bu_6, C_{96}-*t*Bu_8, C_{114}-*t*Bu_{10} and C_{132}-*t*Bu_{12} 23

Supplementary Figure 7. Molecular structure of the different conformers of the C_{96}-*t*Bu_8 24

Supplementary Figure 8. Additional calculations on the C_{78}-*t*Bu_6 GQD 28

Supplementary Figure 9. Transition densities for the C_{78}-*t*Bu_6, C_{96}-*t*Bu_8 and C_{114}-*t*Bu_{10} structures 29

Supplementary Figure 10. Vibrations coupled with S0 -> S1 transition. 30

Supplementary Figure 11. Absorption, photoluminescence and time-resolved photoluminescence of C_{78}-*t*Bu_6, C_{96}-*t*Bu_8, C_{114}-*t*Bu_{10} and C_{132}-*t*Bu_{12} 31

Supplementary Figure 12. Absorption and emission spectra of C_{78}-*t*Bu_6, C_{96}-*t*Bu_8, C_{114}-*t*Bu_{10} and C_{132}-*t*Bu_{12} in 1,2,4-trichlorobenzene, dichloromethane, tetrahydrofuran and toluene. 33

Supplementary Figure 13. Photoluminescence Quantum Yield measurements of C_{78}-*t*Bu_6, C_{96}-*t*Bu_8, C_{114}-*t*Bu_{10}, C_{132}-*t*Bu_{12} and Fluoresceine. 34

Supplementary Figure 14. Photoluminescence C_{96}-*t*Bu_8, C_{114}-*t*Bu_{10}, C_{132}-*t*Bu_{12} at two different wavelength 35

Aggregation of GQDs 36

NMR Spectra (400 MHz) 40

^{1}H and ^{13}C NMR of (4,4''-di-*tert*-butyl-[1,1':4',1''-terphenyl]-2',5'-diyl)bis(trimethylsilane) (6) 40

^{1}H and ^{13}C NMR of 4,4''-di-*tert*-butyl-2',5'-diiodo-1,1':4',1''-terphenyl (7) 42

^{1}H and ^{13}C NMR of terphenyl derivative (8) 43

^{1}H and ^{13}C NMR of dendrimer (2) 44

^{1}H and ^{13}C NMR of terphenyl derivative (10) 45

^{1}H and ^{13}C NMR of monoborylated terphenyl derivative (11) 46

^{1}H and ^{13}C NMR of diterphenyl derivative (12) 47

^{1}H and ^{13}C NMR of diterphenyl derivative (13) 48

^{1}H and ^{13}C NMR of diterphenyl derivative (14) 49

^{1}H and ^{13}C NMR of dendrimer (1) 50

^{1}H and ^{13}C NMR of triterphenyl derivative (15) 51

^{1}H and ^{13}C NMR of triterphenyl derivative (16) 52

^{1}H and ^{13}C NMR of triterphenyl derivative (17) ... 53
^{1}H and ^{13}C NMR of dendrimer (3) ... 54
^{1}H and ^{13}C NMR of ditrimetylsilyl tetraterphenyl derivative (18). ... 55
^{1}H and ^{13}C NMR of diiodo tetraterpheyl derivative (19). ... 56
^{1}H and ^{13}C NMR of di-(trimethylsilyl)-ethynyl tetraterphenyl derivative (20). ... 57
^{1}H and ^{13}C NMR of Dendrimer (4) ... 58
NMR Spectra (600 MHz) ... 59
^{1}H NMR spectra of C_{78}-tBu$_6$ in CS_2+THF-d_8 (250 μL + 350 μL) at 298 K and $5.39 \cdot 10^{-4}$ M ... 59
^{1}H NMR spectra of C_{96}-tBu$_8$ in CS_2+THF-d_8 (250 μL + 350 μL) at 298 K and $5.36 \cdot 10^{-4}$ M ... 62
^{1}H NMR spectra of C_{114}-tBu$_{10}$ in CS_2+THF-d_8 (250 μL + 350 μL) at 298 K and $5.86 \cdot 10^{-4}$ M ... 65
^{1}H NMR spectra of C_{132}-tBu$_{12}$ in CS_2+THF-d_8 (250 μL + 350 μL) at 298 K and $5.54 \cdot 10^{-4}$ M ... 68
Supplementary references ... 71

Supplementary methods

The elongation of the GQDs is performed by adding a row of 4,4′′-di-*tert*-butyl-*p*-terphenyl in the acetylenic core. The synthesis of the GQDs **C_{78}-*t*Bu$_6$**, **C_{96}-*t*Bu$_8$**, **C_{114}-*t*Bu$_{10}$** and **C_{132}-*t*Bu$_{12}$** is based on the Scholl oxidation of the corresponding polyphenylene dendrimers **1**, **2**, **3** and **4**.

(4,4''-di-*tert*-butyl-[1,1':4',1''-terphenyl]-2',5'-diyl)bis(trimethylsilane) (6).

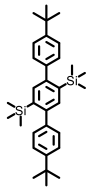

In a round-bottom-flask 1,4-diiodo-2,5-bis(trimethylsilyl)benzene (4 g, 8.50 mmol), 4-*tert*-butylphenylboronic acid (4.54 g, 25.50 mmol), palladium(II) acetate (96 mg, 0.43 mmol), SPhos (355 mg, 0.85 mmol) and potassium phosphate tribasic (3.6 g, 16.86 mmol) were introduced. Toluene (120 mL) and water (12 mL), previously degassed by freeze-pump-thaw, were introduced to the reaction mixture. The reaction was stirred at 90°C under argon for 12 h. The mixture was then filtered through celite and washed with dichloromethane. The organic phase was washed with water and brine then dried with Na_2SO_4, filtered and evaporated. The product was purified by column chromatography (SiO_2, cyclohexanes) and recovered as a white powder (3.70 g, 89% Yield). ^{1}H NMR (400MHz, $CDCl_3$, 298 K): δ(ppm) 7.48 (s, 2H), 7.42 (d, *J* = 8.3 Hz, 4H), 7.29 (d, *J* = 8.3 Hz, 4H), 1.38 (s, 18H), -0.01 (s, 18H). ^{13}C NMR (101 MHz, $CDCl_3$, 298K): δ(ppm) 150.19, 148.88, 141.80, 139.11, 135.78, 129.29, 124.71, 34.71, 31.62, 0.66. MALDI-TOF MS: calc. for $C_{32}H_{46}Si_2$: 486.31; found: 486.31.

4,4''-di-*tert*-butyl-2',5'-diiodo-1,1':4',1''-terphenyl (7).

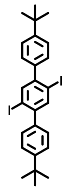

Terphenyl derivative **6** (650 mg, 1.33 mmol) was introduced into a round bottom flask along with freshly distilled non-stabilized dichloromethane (25 mL). The mixture was cooled down in an ice bath to 0°C then iodine monochloride (1 M in dichloromethane, 5.30 mL, 5.30 mmol) added drop wisely. The mixture was allowed to come back to room temperature and stirred for 36 h under argon. ICl was quenched by adding a saturated aqueous solution of sodium thiosulfate. The product was extracted with dichloromethane then washed with water and brine. The organic phase was dried with Na_2SO_4, filtered, and evaporated to give the product as an off-white powder (786 mg, 99% yield). ^{1}H NMR (400MHz, $CDCl_3$, 298 K): δ(ppm) 7.87 (s, 2H), 7.46 (d, *J* = 8.4 Hz, 4H), 7.32 (d, *J* = 8.4 Hz, 4H), 1.38 (s, 18H). ^{13}C NMR (101 MHz, $CDCl_3$, 298K): δ(ppm) 151.17, 146.91, 140.53, 139.47, 128.97, 125.14, 98.30, 34.84, 31.52. MALDI-TOF MS: calc. for $C_{26}H_{28}I_2$: 594.03; found: 594.04.

((4,4''-di-*tert*-butyl-[1,1':4',1''-terphenyl]-2',5'-diyl)bis(ethyne-2,1-diyl))bis(trimethylsilane) (8).

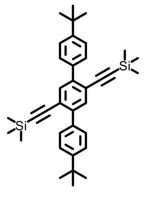

Diiodoterphenyl derivative **7** (1.18 mg, 2.00 mmol), bis(triphenylphosphine)palladium(II) dichloride (175 mg, 0.25 mmol) and copper(I) iodide (150 mg, 1.00 mmol) were dissolved in triethylamine (12 mL) and toluene (8 mL) previously degassed by freeze-pump thaw. Ethynyltrimethylsilane (970 µL, 7.00 mmol)

was added to the mixture which was stirred under argon at 80°C for 24 h. Dichloromethane (DCM) was added to the mixture and filtered through a pad of celite. The filtrate was washed with water and brine, dried with Na_2SO_4, filtered and evaporated and purified by column chromatography (SiO_2, cyclohexanes:dichloromethane 9:1) to give the desired product as a white powder (720 mg, 68% yield). ^{1}H NMR (400MHz, $CDCl_3$, 298 K): δ(ppm) 7.61 – 7.55 (m, *J* = 9.4 Hz, 6H), 7.43 (d, *J* = 8.5 Hz, 4H), 1.37 (s, 18H), 0.13 (s, 18H). ^{13}C NMR (101 MHz, $CDCl_3$, 298K): δ(ppm) 150.71, 142.53, 136.34, 134.08, 129.05, 124.86, 121.80, 104.75, 99.38, 34.74, 31.51, 0.22. MALDI-TOF MS: calc. for $C_{36}H_{46}Si_2$: 534.31; found: 534.31.

4,4''-di-*tert*-butyl-2',5'-diethynyl-1,1':4',1''-terphenyl (8').

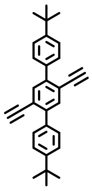

Di-(trimethylsilylacetylene)terphenyl derivative **8** (65 mg, 0.12 mmol) was introduced along with freshly distilled tetrahydrofuran (THF) (10 mL) in a round bottom flask. Tetrabutylammonium fluoride (1 M in THF, 310 µL, 0.31 mmol) was added drop wisely and the mixture left stirring at room temperature under argon for 2 h. The product was extracted with dichloromethane then washed with water and brine. The organic phase was dried with Na_2SO_4, filtered, and evaporated to give a yellowish powder which was used without further purification for the next step.

Dendrimer (2).

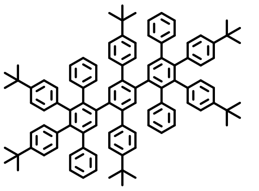

Di-(trimethylsilylacetylene)terphenyl derivative **8'** (46 mg, 0.12 mmol), and 3,4-bis(4-(*tert*-butyl)phenyl)-2,5-diphenylcyclopenta-2,4-dien-1-one (205 mg, 0.41 mmol) were introduced in a round-bottom flask, followed by the addition of dry o-Xylene (4 mL). The solution was degassed sparging argon and then heated to 180°C for 16 h. After the mixture cooled down it was added to an excess methanol to precipitate the product as a white powder and then filtered through a PTFE filter, this process was repeated twice. The product was recovered as a white powder (153 mg, 99% yield). ^{1}H NMR (400MHz, $CDCl_3$, 298 K): δ(ppm) 7.53 (s, 1H), 7.33 (s, 1H), 7.21 – 6.28 (m, 45H), 5.69 (s, 1H), 1.29 (s, 18H), 1.13 (s, 18H), 1.08 (s, 18H). ^{13}C NMR (101 MHz, $CDCl_3$, 298K): δ(ppm) 149.12, 148.07, 147.74, 142.04, 140.21, 139.86, 139.59, 139.36, 138.97, 137.81, 137.65, 137.35, 133.49, 132.86, 131.47, 130.15, 129.36, 127.47, 126.04, 124.87, 124.46, 123.53, 123.08, 77.48, 77.36, 77.16, 76.84, 34.51, 34.26, 34.17, 31.52, 31.36, 31.33. MALDI-TOF MS: calc. for $C_{102}H_{102}$: 1326.80; found: 1326.79.

C_{78}-tBu$_6$.

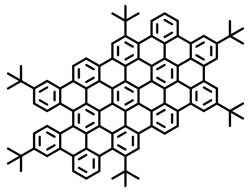

Dendrimer **2** (20 mg, 0.015 mmol) was dissolved in un-stabilized dichloromethane (50 mL). A solution of iron(III) chloride (1 M in $MeNO_2$, 282 mg, 1.74 mmol) was added dropwise to the mixture. The reaction was left at room temperature under a strong flow of extra pure argon for 2 h 30 min. The mixture was then added in one time to methanol (150 mL) and hydrazine (5 mL) was added to the mixture to quench the reaction. The precipitate was filtered through a PTTF filter. The powder was washed with HCl (0.5 M) and dried under vacuum. The powder was solubilized in a small amount of THF, sonicated for 1 min followed by ultracentrifugation. The precipitate was purified in steric exclusion chromatography (THF) and obtained as a dark orange powder (5 mg, 25% yield). ^{1}H NMR (600 MHz, CS_2+THF-d_8, 298 K): δ(ppm) 10.00 (d, *J* = 7.2 Hz, 2H), 9.92 (s, 2H), 9.65 (d, *J* = 7.0 Hz, 2H), 9.54 (s, 2H), 9.18 (s, 2H), 8.96 (t, *J* = 6.9 Hz, 2H), 8.92 (s, 2H), 8.41 (s, 2H), 7.96 (d, *J* = 8.4 Hz, 2H), 7.86 (d, *J* = 8.1 Hz, 2H), 2.22 (s, 18H), 1.83 (s, 18H), 1.79 (s, 18H). ^{13}C NMR (151 MHz, CS_2+THF-d_8, 298 K, only carbons visible by ^{1}H-^{13}C HSQC and HMBC are reported): δ(ppm) 149.64, 147.88, 143.98, 133.82, 131.47, 131.08, 130.50, 127.37, 127.18, 126.79, 126.59, 125.42, 125.03, 123.07, 123.07, 122.49, 122.29, 120.93, 119.95, 119.36, 119.17, 118.58, 39.47, 36.54, 35.96, 35.76, 32.64, 32.64. MALDI-TOF HRMS: calc. for $C_{102}H_{74}$: 1298.5785; found: 1298.5795.

(4,4''-di-*tert*-butyl-5'-iodo-[1,1':4',1''-terphenyl]-2'-yl)trimethylsilane (10).

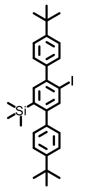

(4,4''-di-*tert*-butyl-[1,1':4',1''-terphenyl]-2',5'-diyl)bis(trimethylsilane) (1.70 g, 3.50 mmol) was dissolved in dry THF (21 mL). Silver tetrafluoroborate (650 mg, 3.30 mmol) was dissolved in anhydrous methanol (7 mL) and then added to the mixture. The reaction mixture was cooled down in an ice bath and Iodine monochloride (1 M in dichloromethane, 3.30 mL, 3.30 mmol) was added drop wisely. The mixture was allowed to heat to room temperature and stirred for 1 h 45 min. A saturated aqueous solution of sodium thiosulfate was added to quench the Iodine monochloride. The aqueous phase was extracted with dichloromethane. The organic phase was then washed with brine, dried with Na_2SO_4, filtered and evaporated. The product was purified by flash chromatography (SiO_2, cyclohexane) and recovered as a white powder (1.17 g, 62 % yield). ^{1}H NMR (400MHz, $CDCl_3$, 298 K): δ(ppm) 7.85 (s, 1H), 7.48 (s, 1H), 7.46 (d, J=8.4 Hz, 2H), 7.41 (d, J=8.3 Hz, 2H), 7.35 (d, J=8.4 Hz, 2H), 7.24 (d, J=8.4 Hz, 2H), 1.39 (s, 9H), 1.39 (s, 9H), -0.02 (s, 9H). ^{13}C NMR (101 MHz, $CDCl_3$, 298K): δ(ppm) 150.78, 150.55, 149.51, 144.20, 141.21, 140.44, 139.73, 138.99, 136.21, 129.19, 129.07, 128.97, 125.14, 125.00, 124.81, 99.65, 34.79, 34.75, 31.58, 31.52, 0.56. MALDI-TOF MS: calc. for $C_{32}H_{46}ISi$: 540.17; found: 540.17.

Monoborylated terphenyl derivative (11).

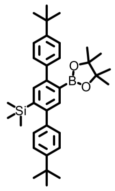

Monoiodoterphenyl derivative **10** (1.08 g, 2.00 mmol), bis(pinacolato)diboron (1.02 g, 4.00 mmol), [1,1'-bis(diphenylphosphino)ferrocene]dichloropalladium(II) (146 mg, 0.20 mmol), and potassium acetate (588 mg, 6.00 mmol) were dissolved in 1,4-dioxane (30 mL) previously degassed by freeze-pump-thaw. The mixture was heated to 95°C under argon for 48 h. The organic phase was then washed with water and brine, dried with Na_2SO_4, filtered and evaporated. The product was purified by chromatography (SiO_2, cyclohexanes:dichloromethane 3:2) and recovered as a white powder (631 mg, 58% yield). ^{1}H NMR (400MHz, CD_2Cl_2, 298 K): δ(ppm) 7.59 (s, 1H), 7.48 (s, 1H), 7.44 – 7.38 (m, 4H), 7.34 (d, *J* = 8.4 Hz, 2H), 7.23 (d, *J* = 8.4 Hz, 2H), 1.35 (s, 18H), 1.18 (s, 12H), -0.01 (s, 9H). ^{13}C NMR (101 MHz, CD_2Cl_2, 298K): δ(ppm) 150.67, 150.40, 147.36, 144.90, 141.69, 141.26, 140.63, 135.71, 135.41, 129.51, 129.14, 125.28, 124.93, 84.16, 34.84, 34.80, 31.59, 31.56, 24.80, 0.57. MALDI-TOF MS: calc. for $C_{35}H_{49}BO_2Si$: 540.36; found: 540.29.

Ditrimethylsilyl diterphenyl derivative (12).

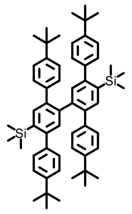

Monoiodoterphenyl derivative **10** (190 mg, 0.36 mmol), monoborylated terphenyl derivative **11** (275 mg, 0.51 mmol), palladium(II) acetate (41 mg, 0.18 mmol), SPhos (148 mg, 0.36 mmol) and potassium phosphate tribasic (152 g, 0.72 mmol) were introduced in a round bottom flask. Previously degassed toluene (19 mL) and water (3 mL) were introduced to the reaction mixture. The reaction was stirred at 80°C under argon for 36 h. Dichloromethane was added to the mixture, the organic phase was washed with water then dried with Na_2SO_4, filtered and evaporated. The product was purified by column chromatography (SiO_2, cyclohexane:dichloromethane 97:3) and recovered as a white powder (247 mg, 83 % Yield). ^{1}H NMR (400MHz, $CDCl_3$, 298 K): δ(ppm) 7.42 (s, 2H), 7.32 (d, J=8.2 Hz, 4H), 7.18 (s, 1H), 7.14 - 7.07 (m, 8H), 6.70 (d, J=8.3 Hz, 4H), 1.37 - 1.32 (m, 18H), 0.00 - -0.02 (m, 18H). ^{13}C NMR (101 MHz, $CDCl_3$, 298K): δ(ppm)150.02, 148.54, 147.89, 141.28, 139.59, 138.50, 138.35, 137.40, 136.52, 133.23, 129.14, 128.91, 127.44, 126.80, 125.90, 124.68, 124.49, 34.64, 34.52, 31.59, 0.81. MALDI-TOF MS: calc. for $C_{58}H_{74}Si_2$: 826.53; found: 826.53.

Diiodo diterphenyl derivative (13).

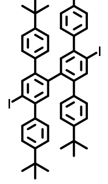

Diterphenyl derivative **12** (195 mg, 0.24 mmol) was dissolved in non-stabilised dichloromethane (5 mL). The reaction mixture was cooled down in an ice bath and iodine monochloride (1 M in dichloromethane, 1 mL, 1 mmol) was added dropwise. The mixture was allowed to heat to room temperature and stirred for 15 h under argon. An aqueous solution of saturated sodium thiosulfate was added. The aqueous phase was extracted with dichloromethane. The organic phase was then washed with water and brine, dried with Na_2SO_4, filtered and evaporated. The product was purified by flash chromatography (SiO_2,

cyclohexane:dichloromethane 97:3) and recovered as a white powder (198 mg, 88 % yield). ^{1}H NMR (400MHz, $CDCl_3$, 298 K): δ(ppm) 7.81 (s, 2H), 7.38 (d, *J* = 8.4 Hz, 4H), 7.21 – 7.10 (m, 10H), 6.69 (d, *J* = 8.3 Hz, 4H), 1.36 (s, 36H). ^{13}C NMR (101 MHz, $CDCl_3$, 298K): δ(ppm) 150.64, 149.62, 145.04, 141.19, 141.07, 140.39, 138.66, 136.33, 133.63, 129.09, 128.68, 125.09, 124.87, 97.53, 34.75, 34.64, 31.54, 31.52. MALDI-TOF MS: calc. for $C_{52}H_{56}I_2$: 934.25; found: 934.25.

Di-(trimethylsilyl)-ethynyl diterphenyl derivative (14).

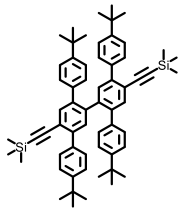

In a round bottom flask, diiodo diterphenyl derivative **13** (82 mg, 0.09 mmol), bis(triphenylphosphine)palladium(II) dichloride (45 mg, 0.06 mmol) and copper(I) iodide (18 mg, 0.12 mmol) were dissolved in triethylamine (6 mL) and toluene (4 mL) previously degassed by freeze-pump thaw. Ethynyltrimethylsilane (46 µL, 0.32 mmol) was added to the mixture which was then stirred under argon at 80°C overnight. Dichloromethane was added to the mixture and filtered through a pad of silica. The filtrate was washed with water and brine, dried with Na_2SO_4, filtered and evaporated and purified by column chromatography (SiO_2, cyclohexanes:chloroform 9:1) to give the desired product as a yellow powder (68 mg, 87% yield). ^{1}H NMR (400MHz, $CDCl_3$, 298 K): δ(ppm) 7.46 (s, 2H), 7.43 (d, *J* = 8.4 Hz, 4H), 7.37 (d, *J* = 8.5 Hz, 4H), 7.33 (s, 2H), 7.10 (d, *J* = 8.3 Hz, 4H), 6.71 (d, *J* = 8.3 Hz, 4H), 1.35 (s, 18H), 1.34 (s, 18H), 0.13 (s, 18H). ^{13}C NMR (101 MHz, $CDCl_3$, 298K): δ(ppm) 150.45, 149.28, 142.57, 139.67, 139.48, 137.09, 136.79, 135.17, 133.10, 132.11, 129.11, 128.75, 128.62, 128.34, 124.95, 124.73, 120.45, 105.19, 97.79, 34.69, 34.58, 31.53, 31.50, -0.15. MALDI-TOF MS: calc. for $C_{62}H_{74}Si_2$: 874.53; found: 874.53.

Diethynyl diterphenyl derivative (14').

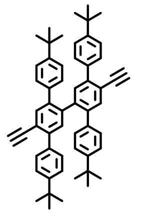

Di-(trimethylsilyl)-ethynyl diterphenyl derivative **14** (63 mg, 0.07 mmol) was dissolved in dry THF (5 mL). Tetrabutylammonium fluoride (1 M in THF, 230 µL, 0.23 mmol) was added dropwise and the brown solution was stirred for 4 h under argon. Dichloromethane and water were then added to the mixture. The organic phase was washed with water and brine, dried with Na_2SO_4, filtered and the solvent evaporated. A yellowish powder was recovered and was used in further reaction without further purification.

Dendrimer (1).

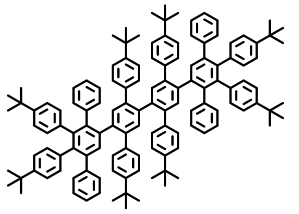

Diethynyl diterphenyl derivative **14'** (20 mg, 0.08 mmol), 3,4-bis(4-(*tert*-butyl)phenyl)-2,5-diphenylcyclopenta-2,4-dien-1-one (114 mg, 0.23 mmol) were dissolved in dry o-Xylene (3 mL). The solution was degassed sparging argon and then heated to 175°C for 16 h. The solvent was evaporated and the product purified by flash column chromatography (SiO_2, cyclohexane:chloroform 87:13 to 4:1) as

a yellow powder (55 mg, 43% yield). ^{1}H NMR (400MHz, $CDCl_3$, 298 K): δ(ppm) 7.57 (s, 1H), 7.40 (s, 1H) 7.34 (s, 1H), 7.18 – 6.41 (m, 55H), 1.36 (s, 18H), 1.29 (s, 18H), 1.13 (s, 18H), 1.09 (s, 18H). ^{13}C NMR (101 MHz, $CDCl_3$, 298K): δ(ppm) 149.25, 148.43, 148.11, 147.82, 142.07, 141.96, 140.39, 139.97, 139.66, 139.43, 138.95, 138.52, 137.61, 137.31, 133.71, 133.27, 132.79, 131.53, 130.12, 129.30, 128.55, 127.49, 126.08, 125.02, 124.51, 124.37, 123.56, 123.16, 34.51, 34.45, 34.27, 34.18, 31.61, 31.51, 31.36, 31.33. MALDI-TOF MS: calc. for $C_{128}H_{130}$: 1667.02; found: 1667.03.

C_{96}-*t*Bu$_8$

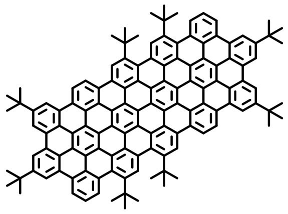

Dendrimer **1** (11.5 mg, 6.90 µmol) was dissolved in non-stabilized dichloromethane (50 mL). A solution of iron(III) chloride (1 M in nitromethane, 185 mg, 1.14 mmol) was added dropwise to the mixture. The reaction was left at room temperature under a strong flow of extra pure argon for 2 h. The mixture was then added to an excess of methanol (150 mL) to precipitate the nanoparticle. The precipitate was filtered through a PTTF filter to recover a greenish powder. The powder was solubilized in a small amount of THF, sonicated for 1 min followed by ultracentrifugation. The precipitate was purified by steric exclusion chromatography (THF) and obtained as a dark green powder (1 mg, 8% yield). ^{1}H NMR (600 MHz, CS_2+THF-d_8, 298 K): δ(ppm) 11.02 (s, 2H), 10.72 (d, *J* = 8.3 Hz, 2H), 10.50 (d, *J* = 8.3 Hz, 2H), 10.23 (s, 2H), 10.05 (s, 2H), 9.71 (s, 2H), 9.62 (s, 4H), 9.56 (d, *J* = 7.5 Hz, 2H), 9.49 (d, *J* = 7.2 Hz, 2H), 8.47 (t, *J* = 7.3 Hz, 2H), 2.42 (s, 18H), 2.08 (s, 18H), 2.03 (s, 18H), 1.99 (s, 18H). ^{13}C NMR (151 MHz, CS_2+THF-d_8, 298 K, only carbons visible by ^{1}H-^{13}C HSQC and HMBC are reported): δ(ppm) 150.23, 150.03, 146.91, 139.87, 135.97, 133.23, 133.04, 131.86, 131.67, 131.28, 131.08, 130.11, 129.91, 129.52, 129.52, 129.13, 129.13, 126.59, 126.59, 126.00, 125.42, 124.83, 124.64, 124.44, 124.64, 123.07, 122.88, 122.88, 122.68, 120.54, 120.34, 120.15, 120.14, 119.95, 119.75, 40.25, 39.47, 36.54, 36.15, 36.15, 35.18, 32.44, 32.44. MALDI-TOF HRMS: calc. for $C_{128}H_{94}$: 1630.7350; found: 1630.7397.

Ditrimethylsilyl triterphenyl derivative (15).

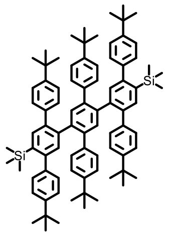

Monoborylated terphenyl derivative **11** (640 mg, 1.18 mmol), diiodo-terphenyl derivative **7** (292 mg, 0.49 mmol), tetrakis(triphenylphosphine)palladium(0) (25 mg, 0.02 mmol), and potassium carbonate (691 mg, 5.00 mmol) were introduced along with previously degassed by freeze-pump-thaw toluene (20 mL), ethanol (4 mL) and water (4 mL). The mixture was stirred under argon at 80°C for 16 h. The mixture was filtered through a celite pad with dichloromethane then washed with water and brine. The organic phase was dried with Na_2SO_4, filtered and evaporated. The product was purified by flash column chromatography (SiO_2, cyclohexanes:dichloromethane 93:7) and recovered as a white powder (372

mg, 65% yield). ^{1}H NMR (400MHz, $CDCl_3$, 298 K): δ(ppm) 7.43 (s, 2H), 7.39 – 7.28 (m, 12H), 7.10 (d, *J* = 8.2 Hz, 4H), 6.96 (d, *J* = 8.3 Hz, 4H), 6.56 (d, *J* = 8.3 Hz, 4H), 6.49 (d, *J* = 8.3 Hz, 4H), 1.36 – 1.32 (m, 54H), 0.03 (s, 18H). ^{13}C NMR (101 MHz, $CDCl_3$, 298K): δ(ppm) 150.18, 148.60, 148.50, 141.17, 140.15, 139.76, 139.02, 138.17, 137.63, 137.48, 136.20, 133.37, 132.73, 129.14, 128.78, 128.51, 124.64, 124.51, 124.23, 34.65, 34.46, 34.43, 31.58, 31.55, 29.86, 0.89, 0.81. MALDI-TOF MS: calc. for $C_{84}H_{102}Si_2$: 1166.75; found: 1166.72.

Diiodo triterphenyl derivative (16).

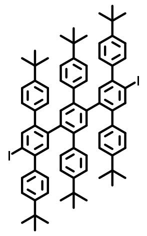

Ditrimethylsilyl triterphenyl derivative **15** (360 mg, 0.31 mmol) was dissolved in freshly distilled non-stabilized dichloromethane (15 mL). The mixture was cooled to 0°C in an ice bath and iodine monochloride (0.5 M in dichloromethane, 2.48 mL, 1.24 mmol) was added dropwise. The mixture was stirred overnight at room temperature under argon, then quenched with a saturated aqueous solution of sodium thiosulfate, extracted with dichloromethane, washed with water and brine, dried with Na_2SO_4, filtered and evaporated. The crude product was purified by flash chromatography (SiO_2, petroleum ether 60/80:chloroform 7:1) and recovered as a white powder (320 mg, 85% yield). ^{1}H NMR (400MHz, $CDCl_3$, 298 K): δ(ppm) 7.75 (s, 2H), 7.46 (s, 2H), 7.42 – 7.38 (m, 7H), 7.31 (s, 2H), 7.12 – 7.06 (m, 6H), 7.03 – 6.97 (m, 4H), 6.53 – 6.44 (m, 7H), 1.34 (s, 54H). ^{13}C NMR (101 MHz, $CDCl_3$, 298K): δ(ppm) 150.71, 140.99, 139.85, 137.08, 135.64, 133.24, 129.16, 128.51, 128.39, 124.95, 124.82, 124.38, 97.29, 34.75, 34.54, 31.54, 31.50, 27.07. MALDI-TOF MS: calc. for $C_{78}H_{84}I_2$: 1274.47; found: 1274.47.

Di-(trimethylsilyl)-ethynyl triterphenyl derivative (17).

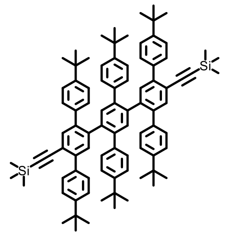

Di-(trimethylsilyl)-ethynyl triterphenyl derivative **16** (310 mg, 0.24 mmol), bis-(triphenylphosphine)palladium(II) dichloride (84 mg, 0.12 mmol) and copper(I) iodide (42 mg, 0.25 mmol) were dissolved in triethylamine (15 mL) and toluene (10 mL) previously degassed by freeze-pump-thaw. Ethynyltrimethylsilane (120 µL, 0.84 mmol) was added to the mixture, which was stirred under argon at 85°C for 21 h. Dichloromethane was added to the mixture and then washed with water and brine, dried with Na_2SO_4, filtered and evaporated and purified by column chromatography (SiO_2, cyclohexanes:dichloromethane 19:1) to give the desired product as a white powder (101 mg, 35% yield). ^{1}H NMR (400MHz, $CDCl_3$, 298 K): δ(ppm) 7.65 (d, *J* = 8.3 Hz, 4H), 7.60 (s, 2H), 7.42 – 7.32 (m, 8H), 7.08 (d, *J* = 8.3 Hz, 4H), 6.96 (d, *J* = 8.3 Hz, 4H), 6.53 (d, *J* = 8.3 Hz, 4H), 6.48 (d, *J* = 8.2 Hz, 4H), 1.35 – 1.31 (m, 54H), 0.15 (s, 18H). ^{13}C NMR (101 MHz, $CDCl_3$, 298K): δ(ppm) 150.55, 148.94, 143.10, 140.74, 139.90, 139.20, 137.16, 136.93, 136.73, 134.70, 133.38, 132.48, 129.11, 128.58, 128.44, 124.81,

124.75, 124.27, 120.44, 105.34, 97.65, 34.70, 34.50, 31.52, 31.50, -0.13. MALDI-TOF MS: calc. for $C_{88}H_{102}Si_2$: 1214.75; found: 1214.60.

Diethynyl triterphenyl derivative (17')

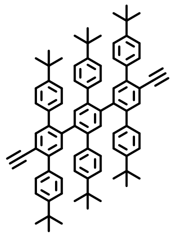

Di-(trimethylsilyl)-ethynyl triterphenyl derivative **17** (93 mg, 0.08 mmol) and freshly distilled THF (8 mL) were introduced in a round bottom flask. Tetrabutylammonium fluoride (1 M in THF, 228 µL, 0.23 mmol) was added dropwise and the brown solution was stirred for 2 h under argon. Dichloromethane and water were then added to the mixture. The organic phase was washed with water and brine, dried with Na_2SO_4, filtered and the solvent evaporated. A yellowish powder was recovered and was used in further reaction without further purification.

Dendrimer (3).

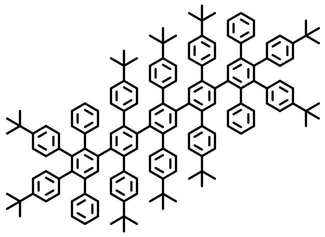

Diethynyl triterphenyl derivative **17'** (85 mg, 0.08 mmol), 3,4-bis(4-(*tert*-butyl)phenyl)-2,5-diphenylcyclopenta-2,4-dien-1-one (114 mg, 0.23 mmol) were dissolved in dry o-Xylene (4 mL). The solution was degassed sparging argon and then heated to 180°C for 16 h. The solvent was evaporated and the product purified by flash column chromatography (SiO_2, cyclohexane:dichloromethane 17:3) as a yellow powder (110 mg, 72% yield). ^{1}H NMR (400MHz, $CDCl_3$, 298 K): δ(ppm) 7.57 (s, 2H), 7.46 (s, 2H), 7.41 (s, 2H), 7.36 (s, 2H), 7.15 – 6.44 (m, 60H), 1.35 (s, 18H), 1.30 (s, 18H), 1.28 (s, 18H), 1.14 (s, 18H), 1.09 (s, 18H). ^{13}C NMR (101 MHz, $CDCl_3$, 298K): δ(ppm) 149.30, 148.75, 148.61, 148.13, 147.84, 142.12, 141.93, 140.42, 139.94, 139.88, 139.77, 139.73, 139.68, 139.46, 139.27, 138.95, 137.61, 137.55, 137.30, 133.50, 133.34, 132.89, 131.53, 130.82, 130.08, 129.22, 128.63, 128.52, 127.48, 126.07, 125.03, 124.55, 124.36, 124.17, 123.56, 123.17, 34.50, 34.45, 34.39, 34.27, 34.19, 31.60, 31.50, 31.36, 31.33, 29.85. MALDI-TOF MS: calc. for $C_{154}H_{158}$: 2007.24; found: 2007.13.

C_{114}-*t*Bu$_{10}$.

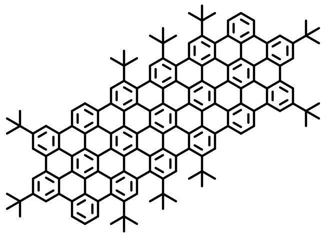

Dendrimer **3** (50 mg, 0.025 mmol) was dissolved in non-stabilized dichloromethane (50 mL). A solution of iron(III) chloride (1 M in nitromethane, 733 mg, 4.50 mmol) was added dropwise to the mixture. The reaction was left at room temperature under a strong flow of extra pure argon for 2 h 15 min. The mixture was then added to an excess of methanol (150 mL) to precipitate the nanoparticle. The precipitate was filtered through a PTTF filter to recover a greenish powder. The powder was solubilized in a small amount of THF, sonicated for 1 min followed by ultracentrifugation. The precipitate was purified by steric exclusion chromatography

(THF) and obtained as a dark green powder (3 mg, 6% yield). ^{1}H NMR (600 MHz, CS_2+THF-d_8, 298 K): δ(ppm) 11.13 (s, 2H), 10.80 (d, *J* = 8.2 Hz, 2H), 10.62 (s, 2H), 10.55 (d, *J* = 8.9 Hz, 2H), 10.43 (s, 2H), 10.08 (s, 2H), 9.73 (s, 2H), 9.65 – 9.60 (m, 4H), 9.55 (d, *J* = 7.3 Hz, 2H), 9.48 (d, *J* = 7.3 Hz, 2H), 8.47 (t, *J* = 7.2 Hz, 2H), 2.56 (s, 18H), 2.31 (s, 18H), 2.13 (s, 18H), 2.10 (s, 18H), 2.00 (s, 18H). ^{13}C NMR (151 MHz, CS_2+THF-d_8, 298 K, only carbons visible by ^{1}H-^{13}C HSQC and HMBC are reported): δ(ppm) 150.42, 150.03, 146.90, 140.65, 140.45, 136.16, 135.77, 133.42, 133.04, 131.86, 131.86, 131.86, 131.47, 130.88, 130.88, 129.91, 129.91, 129.91, 129.71, 129.71, 129.52, 129.52, 128.93, 128.93, 128.34, 126.59, 126.59, 126.40, 126.00, 125.61, 125.02, 124.63, 124.63, 124.44, 123.46, 123.27, 123.07, 122.87, 122.87, 122.68, 122.49, 120.73, 120.53, 120.34, 120.14, 119.94, 119.75, 119.56, 40.44, 39.86, 39.47, 36.34, 36.34, 36.34, 35.56, 35.37, 32.63, 32.44. MALDI-TOF HRMS: calc. for $C_{154}H_{114}$: 1962.8915; found: 1962.8854.

Ditrimetylsilyl tetraterphenyl derivative (18).

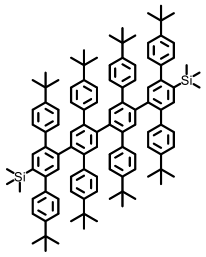

Monoborylated terphenyl derivative **11** (222 mg, 0.41 mmol), diiodo diterphenyl derivate **13** (163 mg, 0.17 mmol), tetrakis(triphenylphosphine)palladium(0) (39 mg, 0.03 mmol), and potassium carbonate (117 mg, 0.85 mmol) were introduced along with previously degassed by freeze-pump-thaw toluene (10 mL), ethanol (2 mL) and water (2 mL). The mixture was stirred under argon at 85°C for 20 h. The mixture was filtered through a celite pad with dichloromethane then washed with water and brine. The organic phase was dried with Na_2SO_4, filtered and evaporated. The product was purified by flash column chromatography (SiO_2, cyclohexanes:dichloromethane 9:1) and recovered as a white powder (186 mg, 72% yield). ^{1}H NMR (400MHz, $CDCl_3$, 298 K): δ(ppm) 7.49 (s, 2H), 7.46 (s, 2H), 7.43 – 7.36 (m, 8H), 7.32 (d, *J* = 8.3 Hz, 4H), 7.13 – 7.07 (m, 8H), 6.92 (d, *J* = 8.3 Hz, 4H), 6.63 (d, *J* = 8.3 Hz, 4H), 6.56 (d, *J* = 8.3 Hz, 4H), 6.46 (d, *J* = 8.4 Hz, 4H), 1.37 (s, 18H), 1.36 – 1.32 (m, 36H), 1.30 (s, 18H), 0.05 (s, 18H). ^{13}C NMR (101 MHz, $CDCl_3$, 298K): δ(ppm) 150.19, 148.99, 148.60, 148.53, 141.19, 140.13, 140.08, 139.59, 139.20, 139.07, 138.18, 138.14, 137.70, 137.43, 137.20, 136.26, 133.28, 133.01, 132.69, 129.12, 128.77, 128.31, 124.67, 124.54, 124.47, 124.25, 34.67, 34.42, 31.68, 31.59, 31.52, 27.06, 0.90. MALDI-TOF MS: calc. for $C_{110}H_{130}Si_2$: 1506.97; found: 1506.94.

Diiodo tetraterpheyl derivative (19).

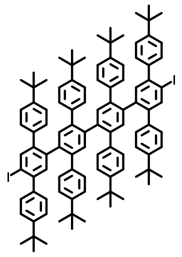

Ditrimetylsilyl tetraterphenyl derivative **18** (166 mg, 0.11 mmol) was dissolved in freshly distilled non-stabilized dichloromethane (8 mL). The mixture was cooled to 0°C in an ice bath and iodine monochloride (1 M in dichloromethane, 0.44 mL, 0.44 mmol) was added dropwise. The mixture was allowed to come back to room temperature and was stirred overnight under argon, then quenched with a saturated

aqueous solution of sodium thiosulfate, extracted with dichloromethane, washed with water and brine, dried with Na_2SO_4, filtered and evaporated. The crude product was purified by flash chromatography (SiO_2, cyclohexanes:dichloromethane 9:1) and recovered as an off white powder (164 mg, 93% yield). ^1H NMR (400MHz, $CDCl_3$, 298 K): δ(ppm) 7.75 (s, 2H), 7.50 (s, 2H), 7.43 – 7.36 (m, 12H), 7.11 – 7.06 (m, 8H), 6.93 (d, *J* = 8.4 Hz, 4H), 6.59 (d, *J* = 8.1 Hz, 4H), 6.50 (d, *J* = 8.2 Hz, 4H), 6.43 (d, *J* = 8.2 Hz, 4H), 1.36 (s, 18H), 1.33 (s, 18H), 1.32 (s, 18H), 1.29 (s, 18H). ^{13}C NMR (101 MHz, $CDCl_3$, 298K): δ(ppm) 150.70, 149.37, 149.22, 148.94, 145.46, 141.06, 140.60, 140.39, 140.31, 140.06, 139.70, 137.24, 136.94, 136.26, 133.18, 131.86, 129.15, 128.70, 128.52, 128.23, 124.95, 124.70, 124.37, 124.32, 97.21, 34.77, 34.47, 31.52, 31.47. MALDI-TOF MS: calc. for $C_{104}H_{112}I_2$: 1614.69; found: 1614.72.

Di-(trimethylsilyl)-ethynyl tetraterphenyl derivative (20).

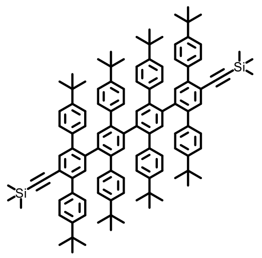

Diiodo tetraterpheyl derivative **19** (164 mg, 0.1 mmol), bis-(triphenylphosphine)palladium(II) dichloride (35 mg, 0.05 mmol) and copper(I) iodide (15 mg, 0.1 mmol) were dissolved in triethylamine (6 mL) and toluene (4 mL) previously degassed by freeze-pump-thaw along with ethynyltrimethylsilane (55 µL, 0.4 mmol). The mixture was stirred under argon at 80°C for 24 h. Dichloromethane was added to the mixture and then washed with water and brine, dried with Na_2SO_4, filtered and evaporated and purified by column chromatography (SiO_2, cyclohexanes:dichloromethane 19:1) to give the desired product as a white powder (70 mg, 45% yield). ^1H NMR (400MHz, $CDCl_3$, 298 K): δ(ppm) 7.70 – 7.64 (m, 5H), 7.45 – 7.38 (m, 10H), 7.15 – 7.06 (m, 9H), 6.90 (d, *J* = 8.3 Hz, 4H), 6.63 (d, *J* = 8.3 Hz, 4H), 6.53 (d, *J* = 8.3 Hz, 4H), 6.44 (d, *J* = 8.3 Hz, 4H), 1.37 (s, 18H), 1.36 – 1.33 (m, 36H), 1.28 (s, 18H), 0.16 (s, 18H). ^{13}C NMR (101 MHz, $CDCl_3$, 298K): δ(ppm) 150.55, 149.17, 149.11, 148.86, 143.07, 140.86, 140.19, 139.66, 139.41, 139.28, 138.69, 137.29, 136.98, 136.94, 136.75, 134.75, 133.17, 133.14, 132.37, 129.11, 128.71, 128.61, 128.29, 124.84, 124.77, 124.63, 124.33, 124.26, 120.42, 105.36, 97.65, 34.71, 34.46, 34.43, 31.53, 31.50, 27.06, -0.13. MALDI-TOF MS: calc. for $C_{114}H_{130}Si_2$: 1554.97; found: 1555.01.

Diethynyl tetraterphenyl derivative (20').

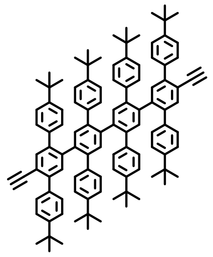

Di-(trimethylsilyl)-ethynyl tetraterphenyl derivative **20** (35 mg, 0.02 mmol) and freshly distilled THF (3 mL) were introduced in a round bottom flask. Tetrabutylammonium fluoride (1 M in THF, 80 µL, 0.08 mmol) was added dropwise and the brown solution was stirred for 2 h under argon. Dichloromethane and water were then added to the mixture. The organic phase was washed with water and brine, dried with Na_2SO_4, filtered and the solvent evaporated. A yellowish powder was recovered and was used in further reaction without further purification.

Dendrimer (4).

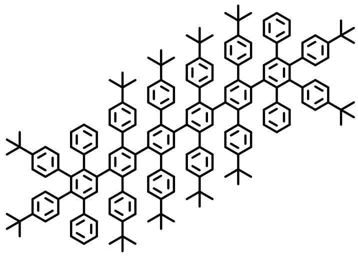

Di-(trimethylsilyl)-ethynyl tetraterphenyl derivative **20'** (35 mg, 0.02 mmol), 3,4-bis(4-(*tert*-butyl)phenyl)-2,5-diphenylcyclopenta-2,4-dien-1-one (45 mg, 0.08 mmol) were dissolved in dry o-Xylene (4 mL). The solution was degassed sparging argon and then heated to 180°C for 18 h. The solvent was evaporated and the product purified by flash column chromatography (SiO_2, cyclohexanes:dichloromethane 87:13) as a yellow powder (29 mg, 59% yield). ^{1}H NMR (400MHz, $CDCl_3$, 298 K): δ(ppm) 7.57 (s, 2H), 7.48 (s, 2H), 7.45 (s, 2H), 7.42 (s, 2H), 7.37 (s, 2H), 7.13 – 6.73 (m, 52H), 6.61 – 6.43 (m, 20H), 1.34 (s, 18H), 1.32 – 1.29 (m, 36H), 1.28 (s, 18H), 1.13 (s, 18H), 1.09 (s, 18H). ^{13}C NMR (101 MHz, $CDCl_3$, 298K): δ(ppm) 149.31, 148.99, 148.76, 148.63, 148.13, 147.84, 142.12, 141.93, 140.44, 139.95, 139.74, 139.45, 139.26, 138.98, 137.61, 137.54, 137.29, 133.52, 133.15, 132.99, 132.88, 131.53, 130.82, 130.08, 129.19, 128.66, 128.43, 127.48, 126.08, 125.02, 124.56, 124.35, 124.21, 123.56, 123.18, 34.51, 34.44, 34.40, 34.27, 34.19, 31.58, 31.50, 31.36, 31.33, 27.07. MALDI-TOF MS: calc. for $C_{180}H_{186}$: 2347.46; found: 2347.50.

C_{132}-tBu$_{12}$

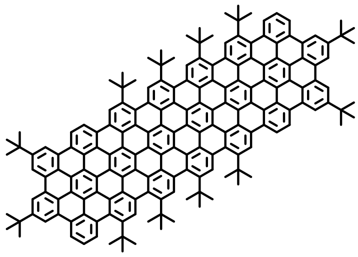

Dendrimer **4** (10 mg, 0.004 mmol) and 2,3-Dichloro-5,6-dicyano-1,4-benzoquinone (43 mg, 0.190 mmol) were dissolved in freshly distilled un-stabilized dichloromethane (15 mL). The solution was allowed to cool down to 0°C in an ice bath. Triflic acid (0.9 mL) was slowly added to the mixture. The solution is stirred for 40 min under argon at 0°C. Distilled trimethylamine (5 mL) was added to quench the reaction and the mixture is then added in one time to methanol (100 mL). The precipitate was filtered through a PTTF filter. The obtained powder was solubilized in a small amount of THF followed by ultracentrifugation. The precipitate yielded the product as a dark green powder (3 mg, 33%). ^{1}H NMR (600 MHz, CS_2+THF-d_8, 298 K): δ(ppm) 11.17 (s, 2H), 10.89 – 10.81 (m, 4H), 10.77 (s, 2H), 10.58 (d, J = 8.5 Hz, 2H), 10.42 (s, 2H), 10.10 (s, 2H), 9.74 (s, 2H), 9.67 – 9.61 (m, 4H), 9.58 (d, J = 7.2 Hz, 2H), 9.51 (d, J = 7.0 Hz, 2H), 8.49 (t, J = 7.0 Hz, 2H), 2.61 (s, 18H), 2.46 (s, 18H), 2.42 (s, 18H), 2.13 (s, 18H), 2.11 (s, 18H), 2.01 (s, 18H). ^{13}C NMR (151 MHz, CS_2+THF-d_8, 298 K, only carbons visible by ^{1}H-^{13}C HSQC and HMBC are reported): δ(ppm) 150.42, 150.23, 147.10, 141.24, 140.65, 140.46, 136.16, 135.97, 135.77, 133.62, 133.23, 131.86, 131.86, 131.86, 131.67, 130.89, 130.89, 130.11, 130.11, 129.91, 129.91, 129.72, 129.72, 129.13, 129.13, 126.59, 126.59, 126.40, 126.00, 125.81, 125.03, 124.83, 124.83, 123.86, 123.66, 123.47, 123.27, 123.07, 123.07, 122.88, 122.68, 120.93, 120.54, 120.34, 120.14, 119.75, 119.75, 40.64, 40.06,

40.06, 39.67, 36.54, 36.54, 36.35, 35.96, 35.76, 35.37, 32.64, 32.44. MALDI-TOF MS HMRS: calc. for $C_{180}H_{134}$: 2295.0480; found: 2295.0502.

Supplementary figures and supplementary tables

Supplementary Figure 1. Structure and optical characterization of the triangular-shaped C_{96}-*t*Bu_6

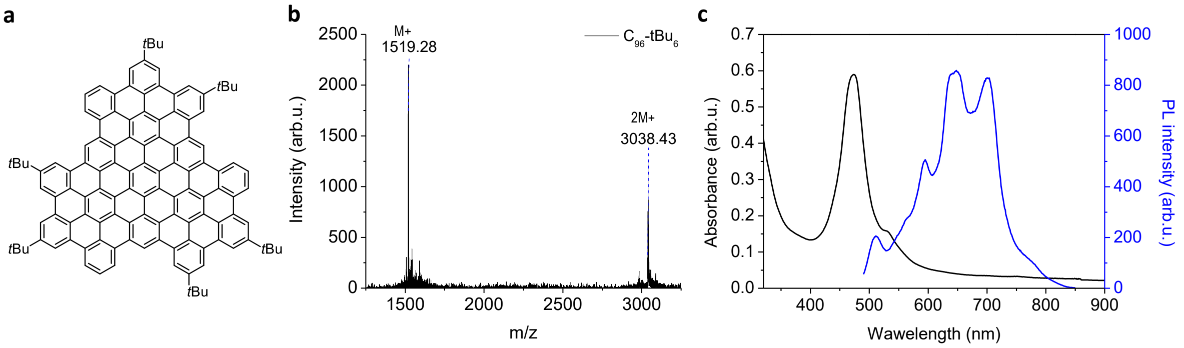

Supplementary Figure 1 | Structure, mass spectrum and optical characterization of the triangular-shaped C_{96}-*t*Bu_6. a) The synthesis of **C_{96}-*t*Bu_6** was adapted from literature procedure;[1] b) mass spectrum of **C_{96}-*t*Bu_6** in TCNQ (7,7,8,8-tetracyanoquinodimethane) as matrix; c) Absorption and emission spectra of **C_{96}-*t*Bu_6** in 1,2,4-trichlorobenzene.

Supplementary Figure 2. Purification and Mass Analysis of C_{78}-*t*Bu_6, C_{96}-*t*Bu_8, C_{114}-*t*Bu_{10} and C_{132}-*t*Bu_{12} GQDs.

In Suppl. Fig. 2, we present the mass spectrum of the oxidation reaction before and after purification. It can be noticed that for all three nanoparticles the main peak observed corresponds to the completely cyclodehydrogenated product (M^+). In the mass spectrum of the crude, peaks at higher *m/z* correspond to partially fused, chlorinated, or hydroxylated side products. Substitution of a *tert*-butyl group by a chlorine atom was also observed, giving rise to the presence of a peak at lower *m/z*. To isolate the desired nanoparticle, first, the powder obtained after oxidation was dissolved in a small amount of THF and left still overnight. The solution was then ultra-centrifugated at 130,000x g for 30 min and the precipitate recovered. For **C_{78}-*t*Bu_6**, no further purification is required since no impurities are observed in the mass spectrum besides a peak at double the mass attributed to the dimer structure. For **C_{96}-*t*Bu_8**, the chlorinated and hydroxylated by-products are still observed. Nonetheless the proportion of the monochlorinated product diminishes after precipitation. In the case of **C_{114}-*t*Bu_{10}**, the precipitation helps to get rid of most of the monochlorinated and monosubstituted by-products. A second purification step is done for **C_{96}-*t*Bu_8** and **C_{114}-*t*Bu_{10}** by size exclusion chromatography (SEC) on Bio-beads S-X1 or S-X3 using THF as eluent. In this step the hydroxylated and the rest of the chlorinated by-products are separated from M^+. Complete cyclodehydrogenation of **C_{132}-*t*Bu_{12}** is achieved using a milder oxidant 2,3-Dichloro-5,6-dicyano-1,4-benzoquinone (DDQ). These milder conditions allow for purification of the GQDs by a single step of ultracentrifugation at 130,000x g for 30 min in THF.

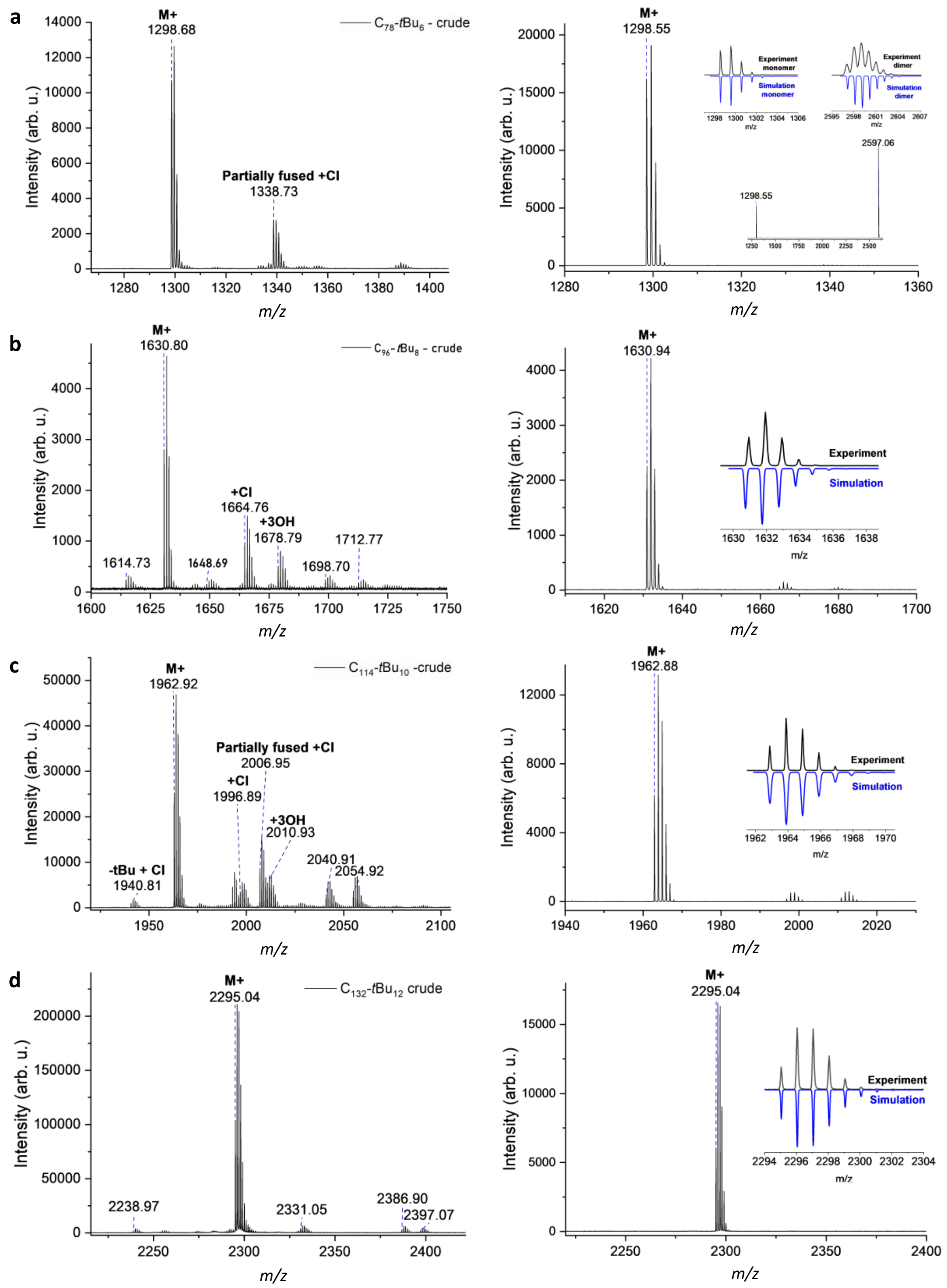

Supplementary Figure 2 | MALDI-ToF MS spectra of C_{78}-*t*Bu$_6$, C_{96}-*t*Bu$_8$, C_{114}-*t*Bu$_{10}$ and C_{132}-*t*Bu$_{12}$. Left part: mass spectra of the crude **C_{78}-*t*Bu$_6$** (a), **C_{96}-*t*Bu$_8$** (b), **C_{114}-*t*Bu$_{10}$** (c) and **C_{132}-*t*Bu$_{12}$** (d) after the Scholl reaction. Right part: mass spectra of the graphene quantum dots after purification. The inserts correspond to the zoom on the $M^{+\bullet}$ peak (black) and a comparison to the simulated spectra (blue).

The insert in Suppl. Fig. 2a (right) show the full spectrum with the signal of the monomer and of the dimer for **C_{78}-*t*Bu$_6$**.

Supplementary Figure 3. Cyclic Voltammetry of GQDs.

The cyclic voltammetry of **C_{78}-*t*Bu_6, C_{96}-*t*Bu_8, C_{114}-*t*Bu_{10} and C_{132}-*t*Bu_{12}** was performed in 1,2-dichlorobenzene (*o*-DCB) because the GQDs present a better solubility in *o*-DCB than in THF, dichloromethane or toluene. In the positive potential region, two reversible oxidation waves were observed for the GQDs while in the negative potential region only partially reversible peaks were observed (Supplementary Figure 3). The half wave potential ($E_{1/2} = \frac{E_{p,a}+E_{p,c}}{2}$) (1) were calculated from experimental peak anodic potential ($E_{p,a}$) and peak cathodic potential ($E_{p,c}$), they are reported in Supplementary Table 1.

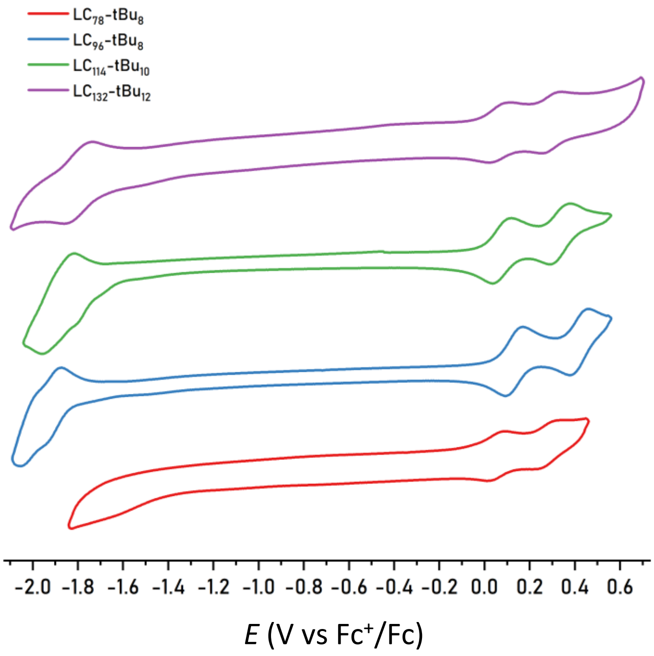

Supplementary Figure 3 | Cyclic voltammetry of C_{78}-*t*Bu_6, C_{96}-*t*Bu_8, C_{114}-*t*Bu_{10} and C_{132}-*t*Bu_{12}. Cyclic voltammograms of **C_{78}-*t*Bu_6**, **C_{96}-*t*Bu_8, C_{114}-*t*Bu_{10}** and **C_{132}-*t*Bu_{12}**. Measurements were done in a 0.1 M tetra-*n*-butylammonium hexafluorophosphate solution in 1,2-dichlorobenzene, with a platinum working electrode at a scan rate of 100 mV s^{-1}.

Supplementary Table 1. Half wave oxidation potentials for C_{78}-*t*Bu_6, C_{96}-*t*Bu_8, C_{114}-*t*Bu_{10} and C_{132}-*t*Bu_{12} in 0.1 M tetra-*n*-butylammonium hexafluorophosphate solution in 1,2-dichlorobenzene. The potential values are reported vs Fc^+/Fc.

GQD	$E_{1/2, ox1}$ (V)	$E_{1/2,ox2}$ (V)
**C_{78}-*t*Bu_6**	0.055	0.285
**C_{96}-*t*Bu_8**	0.132	0.421
**C_{114}-*t*Bu_{10}**	0.078	0.335
**C_{132}-*t*Bu_{12}**	0.062	0.287

Supplementary Figure 4. Comparison of absorption, PL and PLE of C_{96}-*t*Bu_8 and C_{96}-$(C_{12}H_{25})_6$ GQD.

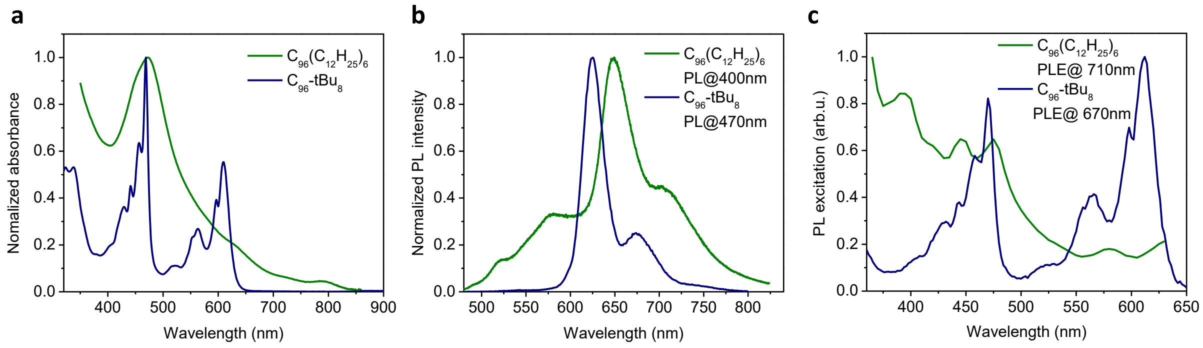

Supplementary Figure 4 | Comparison of C_{96}-*t*Bu_8 and the previously studied C_{96}-$(C_{12}H_{25})_6$ GQD. a) Normalized absorption spectra of **C_{96}-*t*Bu_8** (blue) and **C_{96}-$(C_{12}H_{25})_6$** (green) in 1,2,4-trichlorobenzene; b) normalized emission spectra of **C_{96}-*t*Bu_8** (blue) and **C_{96}-$(C_{12}H_{25})_6$** (green) in 1,2,4-trichlorobenzene; c) normalized PLE spectra of **C_{96}-*t*Bu_8** (blue) and **C_{96}-$(C_{12}H_{25})_6$** (green) in 1,2,4-trichlorobenzene.

Supplementary Figure 5. Normalized absorption spectra of C_{78}-*t*Bu_6, C_{96}-*t*Bu_8, C_{114}-*t*Bu_{10} and C_{132}-*t*Bu_{12}.

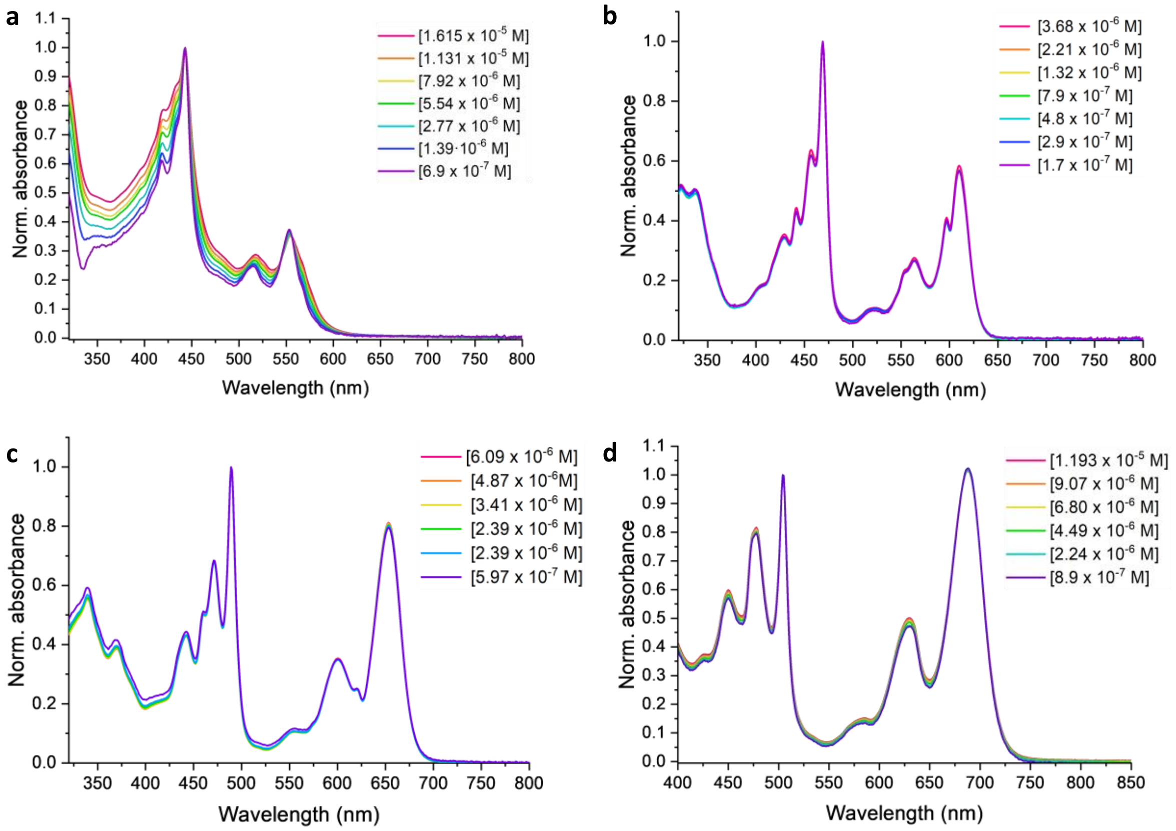

Supplementary Figure 5 | Normalized absorption spectra of C_{78}-*t*Bu_6, C_{96}-*t*Bu_8, C_{114}-*t*Bu_{10} and C_{132}-*t*Bu_{12} as a function of the concentration in 1,2,4-trichlorobenzene. The spectra of **C_{78}-*t*Bu_6** (a) clearly show a broadening of the transition as the concentration increases; it is attributed to the formation of aggregates/multimeric species in solution. The spectra of **C_{96}-*t*Bu_8** (b), **C_{114}-*t*Bu_{10}** (c), and **C_{132}-*t*Bu_{12}** (d) are perfectly reproducible and independent of the concentration (in the concentration range studied).

Supplementary Figure 6. Calculated absorption spectra of C_{78}-*t*Bu_6, C_{96}-*t*Bu_8, C_{114}-*t*Bu_{10} and C_{132}-*t*Bu_{12}

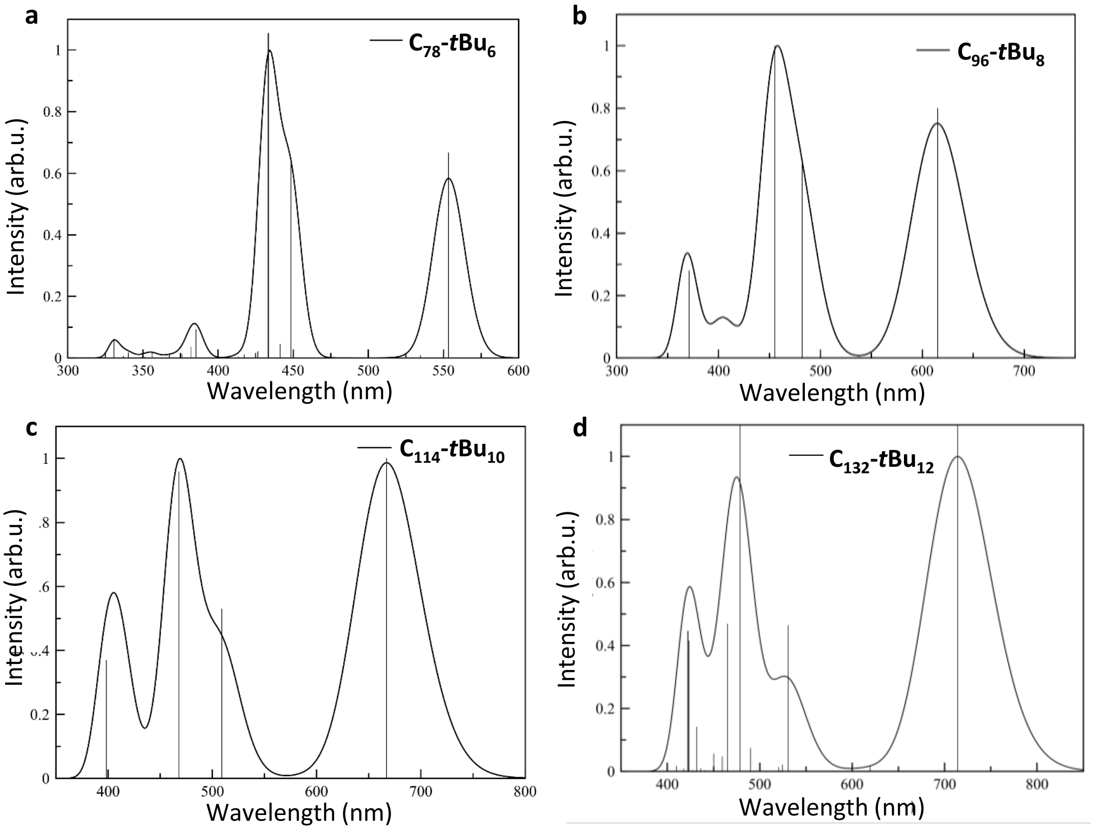

Supplementary Figure 6 | Calculated absorption spectra of the graphene quantum dots. a) **C_{78}-*t*Bu_6**; b) **C_{96}-*t*Bu_8**; c) **C_{114}-*t*Bu_{10}** and d) **C_{132}-*t*Bu_{12}**.

Supplementary Figure 7. Molecular structure of the different conformers of the C_{96}-*t*Bu$_8$

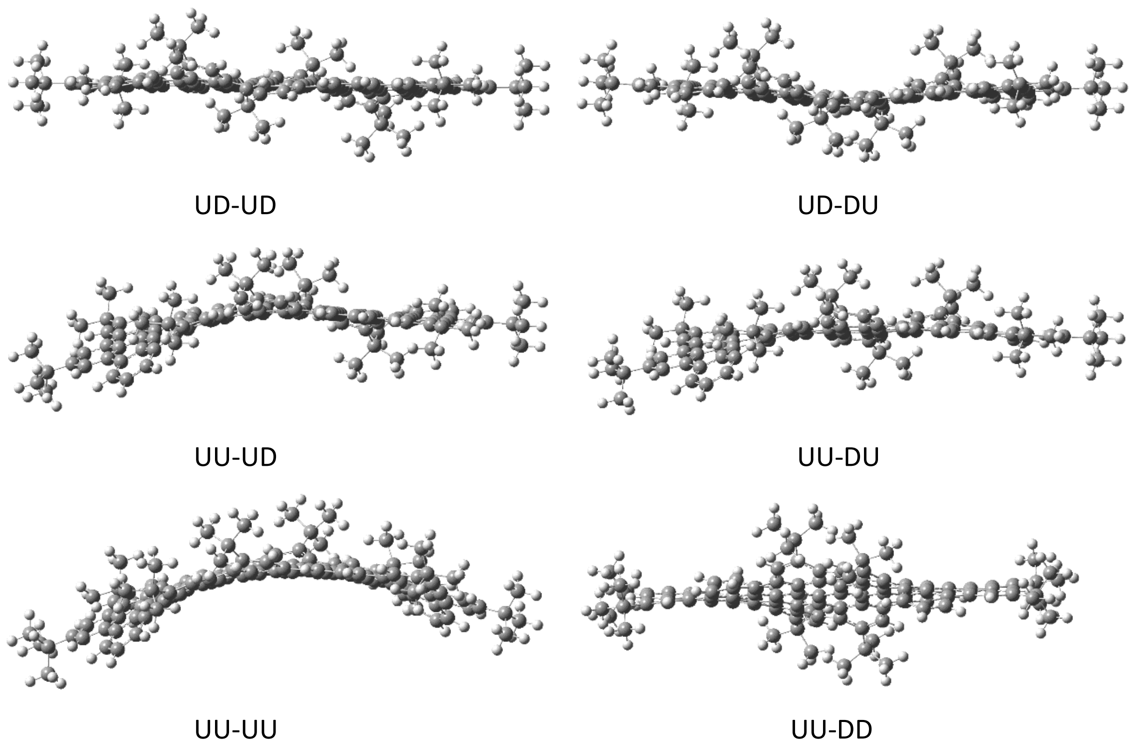

Supplementary Figure 7 | Molecular structure of the conformers of the C_{96}-*t*Bu$_8$ considering different relative positions of the *t*Bu groups along the long axis of the molecule.

Supplementary Table 2 | Absorption and emission wavelengths, oscillator strengths and transition. Only the bright excited states are reported.

C_{78}-*t*Bu_6 Abs	Wavelength in nm (oscillator strength)	Transition	C_{78}-*t*Bu_6 Em	Wavelength in nm (oscillator strength)	Transition
S1	554 (0.66)	H → L	S1	600 (0.82)	H → L
S4	449 (0.63)	H → L+1	S4	461 (0.43)	H → L+1
S6	433 (1.05)	H-1 → L+1	S5	457 (0.24)	H-2 → L
S13	385 (0.09)	H-2 → L+2	S9	425 (0.68)	H-1 → L+1
S30	331 (0.06)	H-5 → L+1			
**C_{96}-*t*Bu_8 Abs**	**Wavelength in nm (oscillator strength)**	**Transition**	**C_{96}-*t*Bu_8 Em**	**Wavelength in nm (oscillator strength)**	**Transition**
S1	615 (1.01)	H → L	S1	669 (1.16)	H → L
S4	482 (0.62)	H → L+1	S6	494 (0.59)	H → L+1
S9	455 (1.19)	H-1 → L+1	S11	444 (0.81)	H-1 → L+1
S17	404 (0.12)	H-2 → L+2			
S25	371 (0.28)	H-3 → L+2	S28	373 (0.23)	H-2 → L+3
**C_{114}-*t*Bu_{10} Abs**	**Wavelength in nm (oscillator strength)**	**Transition**	**C_{114}-*t*Bu_{10} Em**	**Wavelength in nm (oscillator strength)**	**Transition**
S1	667 (1.37)	H → L	S1	728 (1.53)	H → L
S5	509 (0.53)	H → L+1	S7	521 (0.43)	H → L+1
S10	468 (1.32)	H-1 → L+1	S12	457 (1.13)	H-1 → L+1
S27	398 (0.37)	H-3 → L+3	S26	402 (0.26)	H-3 → L+2
**C_{132}-*t*Bu_{12} Abs**	**Wavelength in nm (oscillator strength)**	**Transition**			
S1	714 (1.73)	H → L			
S6	530 (0.46)	H → L+2			

S12	479 (1.17)	H-1 → L+2
S14	465 (0.47)	H-2 → L+1
S27	422 (0.45)	H-3 → L+3

Supplementary Table 3 | Relative energy and frontier orbitals energetics for the different conformers of the **C_{96}-*t*Bu$_8$** molecule. All energies are reported in eV, apart from the total energy which is in Hartree. The UD-UD conformer is considered as reference energy point and its ΔE (in eV) is set to zero in both gas phase and solvent. Excitation energies are reported in eV.

**Conformer**	**UD-UD**	**UD-DU**	**UU-DU**	**UU-UD**	**UU-UU**	**UU-DD**
**LUMO+1**	-2.08	-2.08	-2.07	-2.06	-2.04	-2.06
**LUMO**	-2.51	-2.51	-2.50	-2.50	-2.50	-2.49
**HOMO**	-4.43	-4.43	-4.43	-4.42	-4.41	-4.42
**HOMO-1**	-4.80	-4.80	-4.79	-4.79	-4.49	-4.79
**Energy gap**	1.92	1.92	1.93	1.92	1.91	1.93
**Total E (gas)**	-4929.7182	-4929.7188	-4929.7056	-4929.7072	-4929.6961	-4929.6920
**ΔE (gas)**	0.00	-0.02	0.34	0.30	0.60	0.71
**Total E (solvent)**	-4929.7263	-4929.7270	-4929.7137	-4929.7154	-4929.7043	-4929.7002
**ΔE (solvent)**	0.00	-0.02	0.34	0.30	0.60	0.71
**Excitation energy (gas)**	2.017	2.015	2.022	2.016	2.013	2.031
**Excitation energy (solvent)**	1.972	1.969	1.977	1.969	1.965	1.985

Supplementary Table 4 | Absorption energy and oscillator strength. Energy and oscillator strength for the different conformers of the **C_{96}-*t*Bu$_8$** GQD. Only the bright excited states are reported.

**UD-UD**	**Energy (eV) (oscillator strength)**
S1	2.017 (1.01)
S2	2.168 (0.01)
S4	2.751 (0.62)
S9	2.728 (1.19)
S17	3.068 (0.12)
S25	3.345 (0.28)
**UU-UD**	**Energy (eV) (oscillator strength)**
S1	2.016 (1.02)
S2	2.175 (0.01)
S4	2.57 (0.53)
S9	2.744 (1.10)
S16	3.052 (0.15)
S27	3.389 (0.19)
**UU-UU**	**Energy (eV) (oscillator strength)**
S1	2.013 (1.02)
S2	2.181 (0.01)
S4	2.572 (0.55)
S9	2.758 (1.04)
S15	3.038 (0.15)
S27	3.389 (0.13)

**UD-DU**	**Energy (eV) (oscillator strength)**
S1	2.015 (1.02)
S2	2.169 (0.01)
S4	2.571 (0.61)
S9	2.731 (1.18)
S17	3.066 (0.12)
S25	3.345 (0.24)
**UU-DU**	**Energy (eV) (oscillator strength)**
S1	2.022 (1.02)
S2	2.175 (0.01)
S4	2.571 (0.52)
S9	2.736 (1.08)
S16	3.052 (0.16)
S26	3.371 (0.16)
**UU-DD**	**Energy (eV) (oscillator strength)**
S1	2.031 (1.02)
S2	2.182 (0.01)
S4	2.574 (0.57)
S9	2.738 (1.10)
S15	3.033 (0.20)
S28	3.397 (0.14)

Supplementary Figure 8. Additional calculations on the C_{78}-*t*Bu_6 GQD

Additional TDDFT excited-state calculations were performed on the **C_{78}-*t*Bu_6** GQD using three additional DFT functionals: CAM-B3LYP, PBE0 and wB97xD. The results displayed below show that, compared to HSE and the experimental results, CAM-B3LYP and wB97xD largely overestimate the excitation energies, with PBE0 doing a bit better. It is comforting to find that the overall shape of the optical absorption spectrum (namely the existence of two intense optical transitions) as well as the polarization of the electronic transitions and their nature is method independent. Yet, HSE unambiguously provides a better quantitative description.

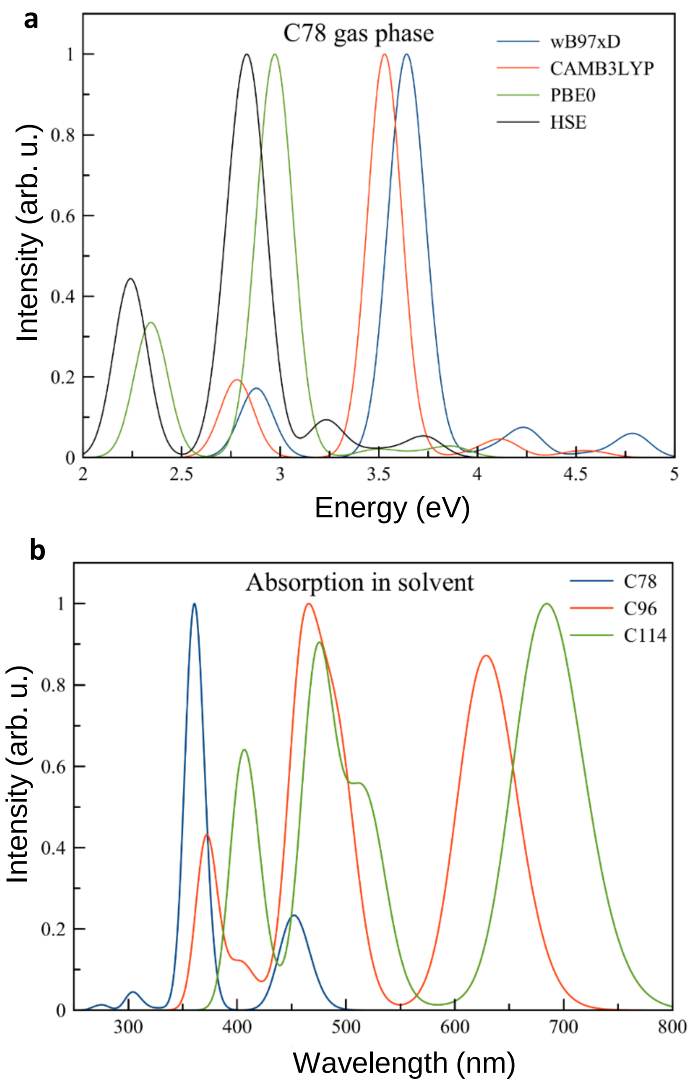

Supplementary Figure 8 | TDDFT excited-state calculations performed on the C_{78}-*t*Bu_6 and simulated absorption. a) Absorption spectra of the **C_{78}-*t*Bu_6** molecule in gas phase obtained with different long-range corrected functionals; b) absorption spectra for the **C_{78}-*t*Bu_6**, **C_{96}-*t*Bu_8** **C_{114}-*t*Bu_{10}** in 1,2,4-trichlorobenzene solvent.

Supplementary Figure 9. Transition densities for the C_{78}-*t*Bu_6, C_{96}-*t*Bu_8 and C_{114}-*t*Bu_{10} structures

**C_{78}-*t*Bu_6**	554 nm	449 nm	433 nm	385 nm	331 nm
Transition density distribu-tion	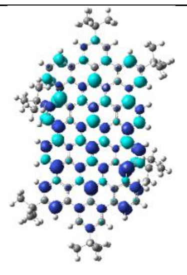	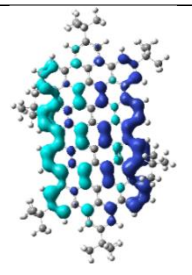	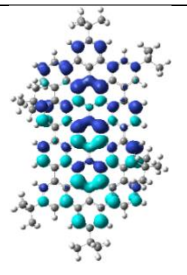	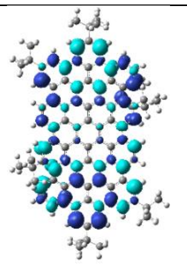	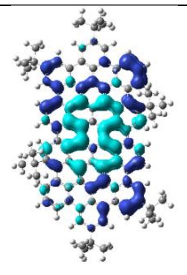
**C_{96}-*t*Bu_8**	615 nm	482 nm	455 nm	404 nm	371 nm
Transition density distribu-tion	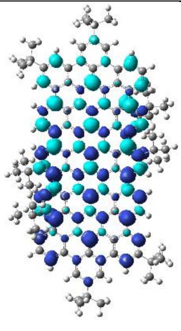	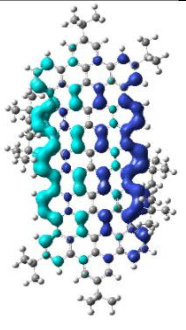	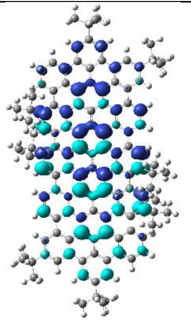	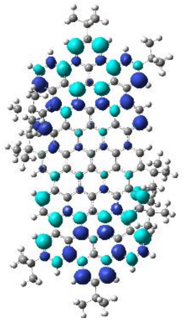	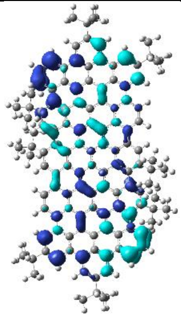
**C_{114}-*t*Bu_{10}**	667 nm	509 nm	468 nm	398 nm	
Transition density distribu-tion	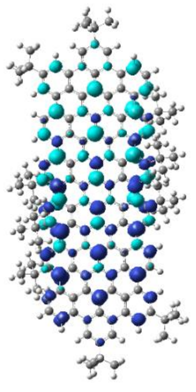	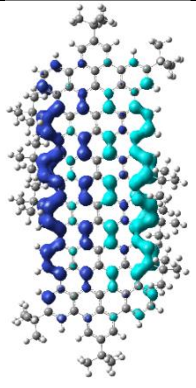	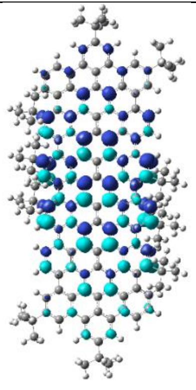	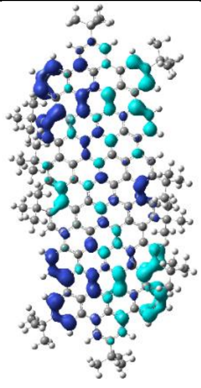	
**C_{132}-*t*Bu_{12}**	714 nm	530 nm	479 nm	465 nm	422 nm
Transition density distribu-tion	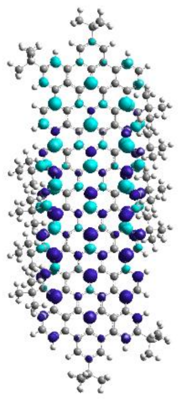	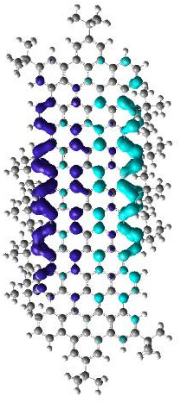	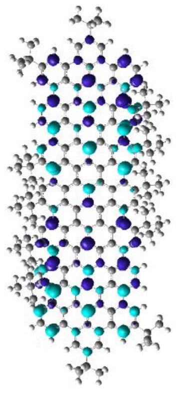	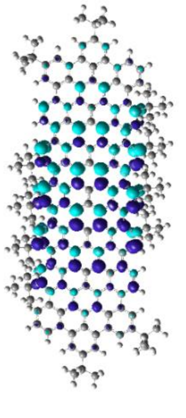	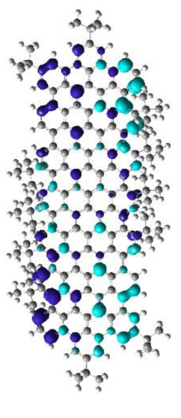

Supplementary Figure 9 | Transition densities for the C_{78}-*t*Bu_6, C_{96}-*t*Bu_8, C_{114}-*t*Bu_{10} and C_{132}-*t*Bu_{12} structures, for the most prominent peaks in the absorption spectrum.

Supplementary Figure 10. Vibrations coupled with S0 -> S1 transition.

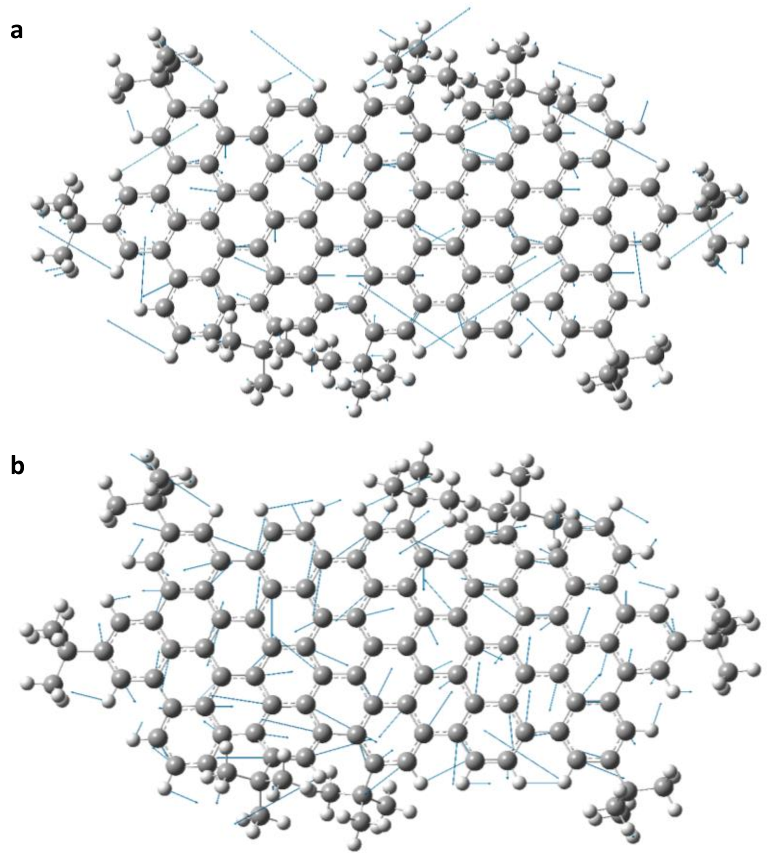

Supplementary Figure 10 | Vibrations coupled with S0 -> S1 transition for the C_{96}-*t*Bu_8 GQD. Panel (a) represents the vibration at 166 meV (1338.88 cm^{-1}); panel (b) represents the vibration at 205 meV (1653.44 cm^{-1}).

Supplementary Figure 11. Absorption, photoluminescence and time-resolved photoluminescence of C_{78}-*t*Bu_6, C_{96}-*t*Bu_8, C_{114}-*t*Bu_{10} and C_{132}-*t*Bu_{12}

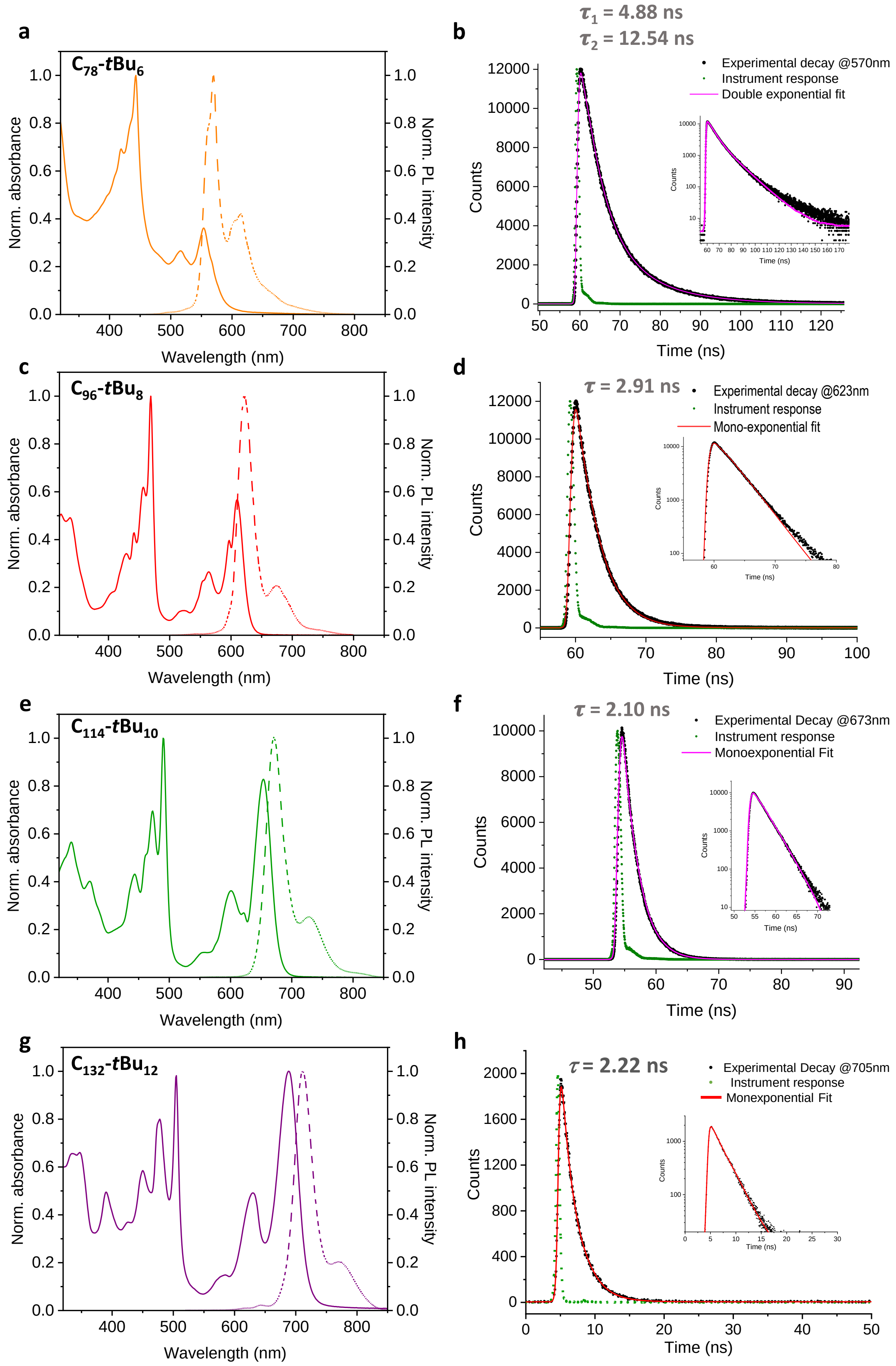

Supplementary Figure 11 | Summary of the absorption, photoluminescence and time-resolved photoluminescence of C_{78}-*t*Bu$_6$, C_{96}-*t*Bu$_8$, C_{114}-*t*Bu$_{10}$ and C_{132}-*t*Bu$_{12}$. Absorption (solid line) and photoluminescence (dashed line) spectra of (a) **C_{78}-*t*Bu$_6$** (orange curve), of (c) **C_{96}-*t*Bu$_8$** (red curve), of (e) **C_{114}-*t*Bu$_{10}$** (green curve) and of (g) **C_{132}-*t*Bu$_{12}$** (purple curve). Time-resolved photoluminescence spectra of (b) **C_{78}-*t*Bu$_6$** (black curve: measurement, pink curve: bi-exponential fit and green curve: IRF (Impulse Response Function)), of (d) **C_{96}-*t*Bu$_8$** (black curve: measurement, red curve: mono-exponential fit and green curve: IRF), of (f) **C_{114}-*t*Bu$_{10}$** (black curve: measurement, pink curve: mono-exponential fit and green curve: IRF) and of (h) **C_{132}-*t*Bu$_{12}$** (black curve: measurement, red curve: mono-exponential fit and green curve: IRF). For **C_{78}-*t*Bu$_6$** (b) the decay is bet fitted by a bi-exponential with a short and long component of 4.88 and 12.54 ns, respectively. The non-mono exponential behavior is attributed to the presence of multimeric species in the solution; for **C_{96}-*t*Bu$_8$** (d), **C_{114}-*t*Bu$_{10}$** (f) and **C_{132}-*t*Bu$_{12}$** (h) the decays are best fitted by a mono exponential and the extracted lifetimes are 2.91, 2.10 and 2.22 ns, respectively. The inset curves are given in Log scale.

Supplementary Figure 12. Absorption and emission spectra of C_{78}-*t*Bu$_6$, C_{96}-*t*Bu$_8$, C_{114}-*t*Bu$_{10}$ and C_{132}-*t*Bu$_{12}$ in 1,2,4-trichlorobenzene, dichloromethane, tetrahydrofuran and toluene.

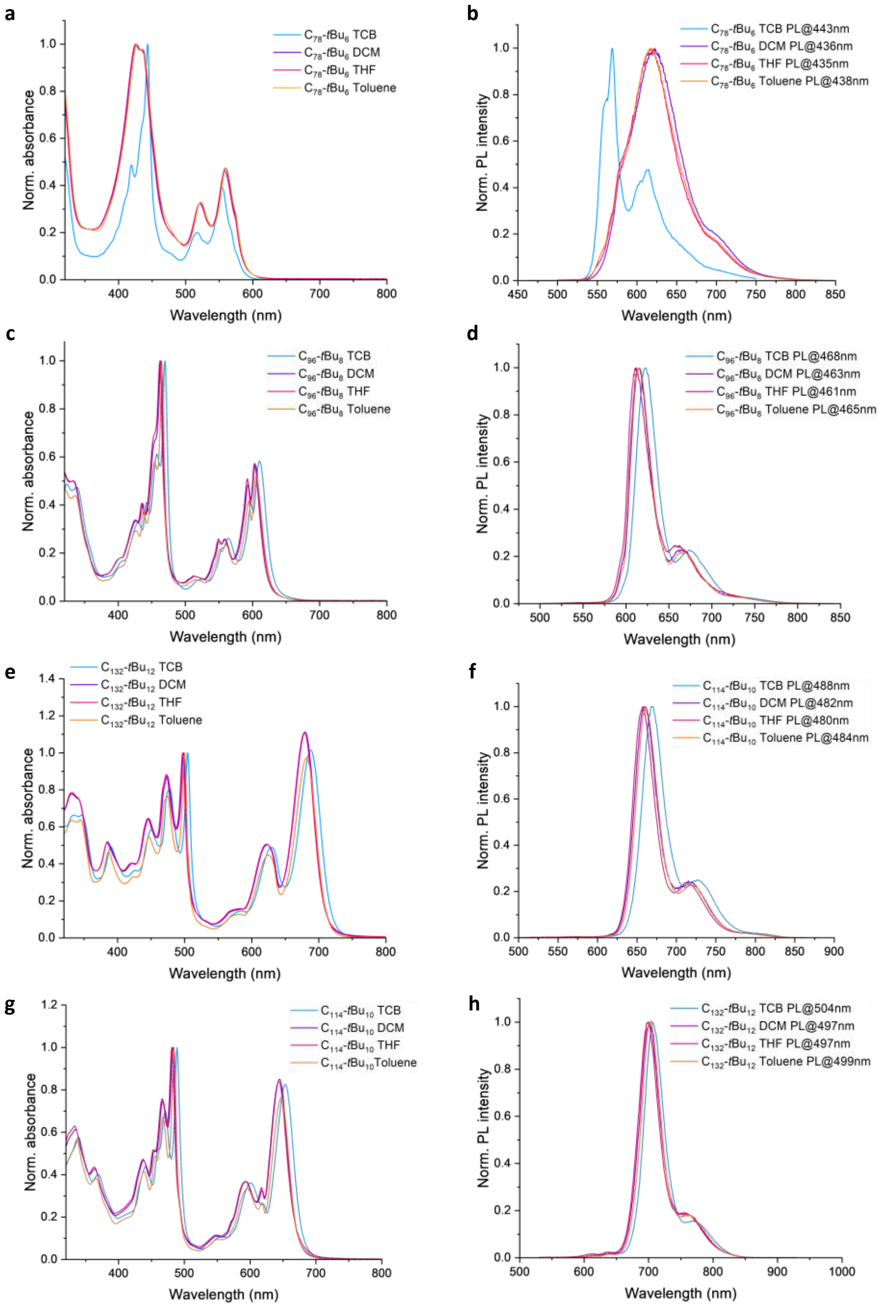

Supplementary Figure 12 | Absorption (left) and photoluminescence (right) of C_{78}-*t*Bu$_6$, C_{96}-*t*Bu$_8$, C_{114}-*t*Bu$_{10}$ and C_{132}-*t*Bu$_{12}$ in different organic solvents. a) absorption, b) photoluminescence (PL) of **C_{78}-*t*Bu$_6$**, c) absorption, d) PL of **C_{96}-*t*Bu$_8$**, e) absorption, f) PL of **C_{114}-*t*Bu$_{10}$**, g) absorption, h) PL of **C_{132}-*t*Bu$_{12}$** in 1,2,4-trichlorobenzene (TCB – blue), dichloromethane (DCM – purple), tetrahydrofuran (THF – pink) and toluene (orange). **C_{96}-*t*Bu$_8$**, **C_{114}-*t*Bu$_{10}$** and **C_{132}-*t*Bu$_{12}$** exhibit similar absorption and PL spectra in all four solvents while **C_{78}-*t*Bu$_6$** exhibits different behaviors in the solvents indicating a rather poor solubility in dichloromethane, tetrahydrofuran, and toluene.

Supplementary Figure 13. Photoluminescence Quantum Yield measurements of C_{78}-*t*Bu_6, C_{96}-*t*Bu_8, C_{114}-*t*Bu_{10}, C_{132}-*t*Bu_{12} and Fluoresceine.

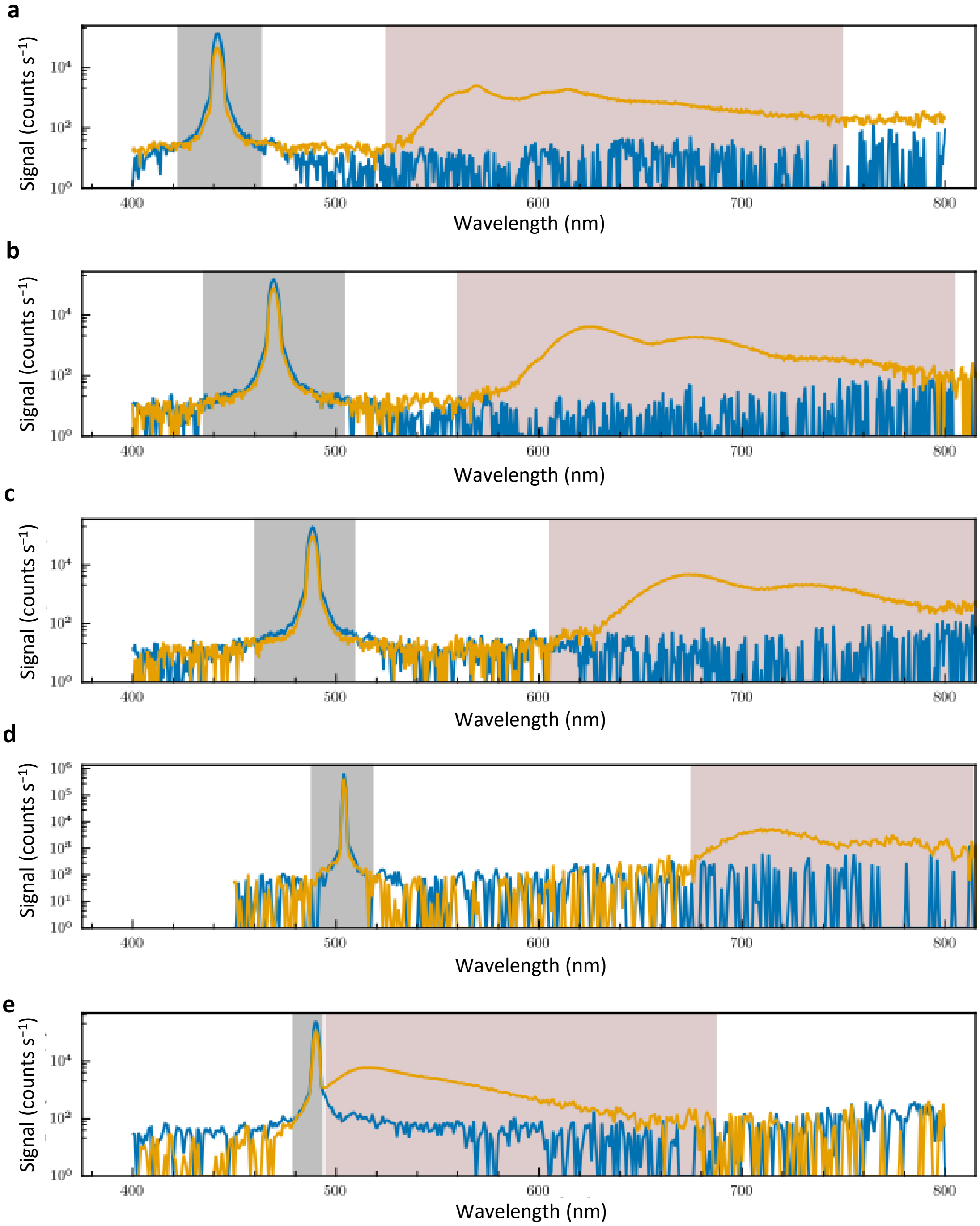

Supplementary Figure 13| PLQY measurements of C_{78}-*t*Bu_6, C_{96}-*t*Bu_8, C_{114}-*t*Bu_{10}, C_{132}-*t*Bu_{12} in 1,2,4 trichlorobenzene, and of Fluoresceine in a 0.1 M NaOH. a) PLQY of **C_{78}-*t*Bu_6** is 56.53%. b) PLQY of **C_{96}-*t*Bu_8** is 94.34% c) PLQY of **C_{114}-*t*Bu_{10}** is 91.06%. d) PLQY of **C_{132}-*t*Bu_{12}** is 88.35%. e) PLQY of Fluoresceine is 91.18%. Blank curves are in blue and measured sample curves are in orange. Scattering region is highlighted in grey, emission region in light red.

Supplementary Figure 14. Photoluminescence C_{96}-*t*Bu_8, C_{114}-*t*Bu_{10}, C_{132}-*t*Bu_{12} at two different wavelength

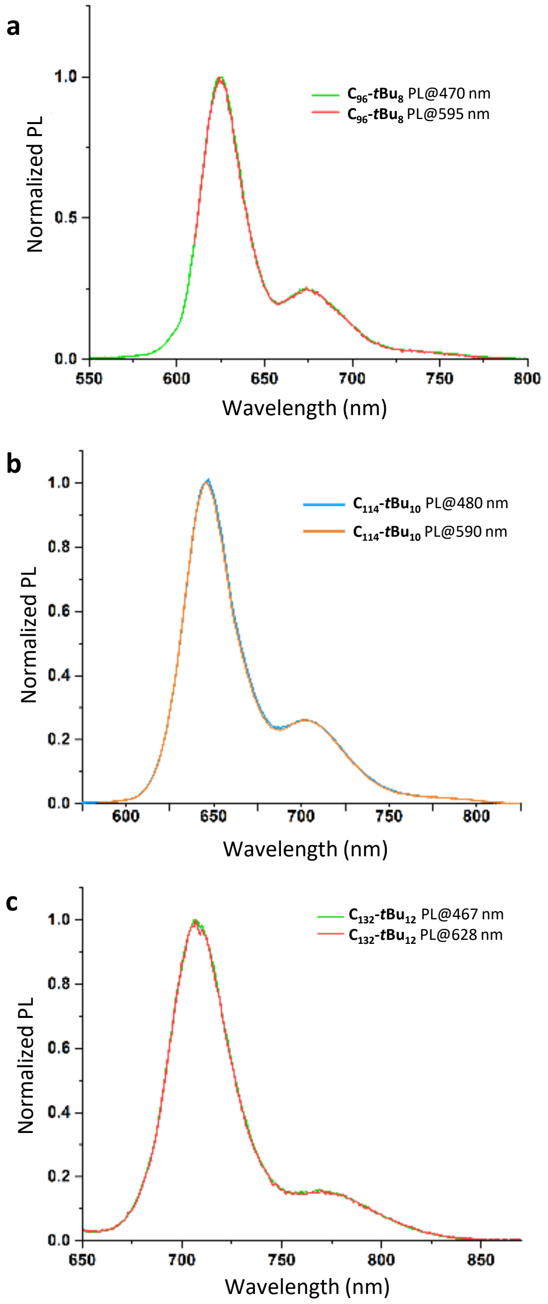

Supplementary Figure 14| PL spectra of C_{96}-*t*Bu_8, C_{114}-*t*Bu_{10}, C_{132}-*t*Bu_{12} at two wavelength in 1,2,4-trichlorobenzene. a) PL spectra of **C_{96}-*t*Bu_8** after excitation at 470 and 595 nm. b) PL spectra of **C_{114}-*t*Bu_{10}** after excitation at 480 and 590 nm. c) PL spectra of **C_{132}-*t*Bu_{12}** after excitation at 467 and 628 nm.

Aggregation of GQDs

Molecular Dynamics Simulation were performed using LAMMPS (Large-scale Atomic/Molecular Massively Parallel Simulator) software[2] and the classical force-field GAFF.[3] The atomic partial charges were assigned with the RESP method, using the Multiwfn package,[4] based on DFT calculations at the B3LYP/6-31g(d,p) using the Gaussian software.[5] All possible conformers were considered during this parameterization.

Umbrella sampling and Weighted Histogram Analysis Method were performed using the PLUMED package.[6] Push and pull trajectories along the reaction coordinate, to generate starting configurations for the Umbrella Sampling, were done by MD simulations in the NPT ensemble at 298.15 K and 1 atm. For the push trajectories, a moving harmonic restraint with a force constant of 2000 kcal mol^{-1} was applied to the GQDs Center Of Mass, starting at an equilibrium distance of 20 Å and down to 2 Å, with a velocity of 0.0005 nm ps^{-1}. The same method was applied to the pull trajectories, starting from local minima configurations obtained from the pushing. Then, evenly spaced configurations were selected along the trajectories, every 0.2 Å, and Umbrella Sampling were performed in these windows by enforcing a harmonic restraint on the GQDs Center Of Mass with a force constant of 50 kcal mol^{-1}. 100 ps of equilibration were first run in each window in the NPT ensemble at 298.15 K and 1 atm., prior to 1 ns of production run.

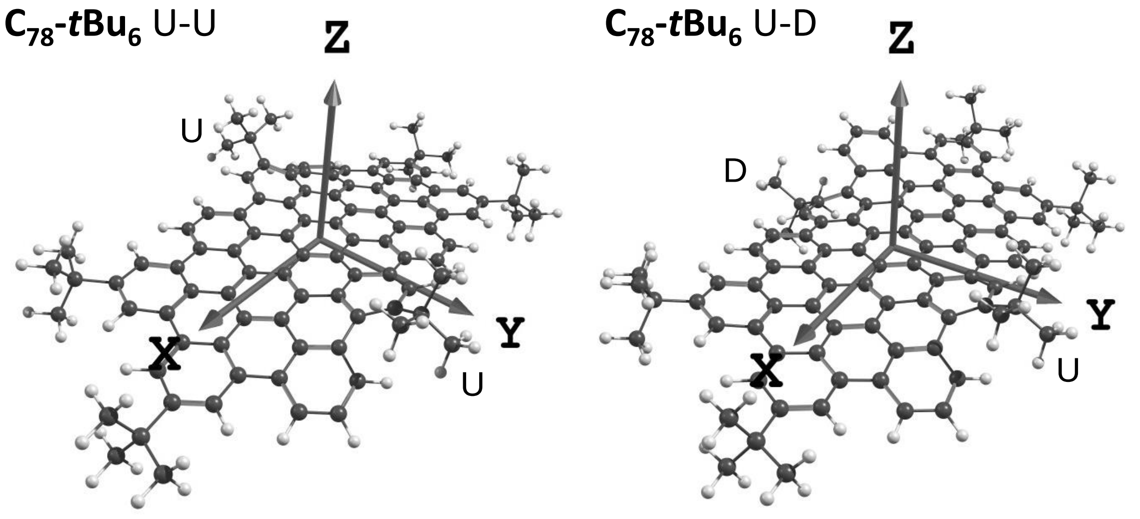

Supplementary Figure 15 | C_{78}-*t*Bu_6 conformers. *Tert*-butyl groups can lie above (U: up) or below (D: down) the molecular plane (XY).

Supplementary Table 5 | C_{78}-*t*Bu_6 conformers' energies and relative abundance at 298.15K

C_{78}-tBu$_6$ conformer	Energy eV @ B3LYP/6-31g(d,p)	Energy eV @ HSE/6-31g(d,p)	Relative abundance @ 293.15K
U-U	0	-	0.5785
U-D	0.008137595	-	0.4215

Supplementary Table 6 | C_{96}-*t*Bu_8 conformers' energies and relative abundance at 298.15K

C_{96}-tBu$_8$ conformer	Energy eV @ B3LYP/6-31g(d,p)	Energy eV @ HSE/6-31g(d,p)	Relative abundance @ 293.15K
UD-DU	0	0	0.6851
UD-UD	0.019972905	0.02	0.3149
UU-UD	0.338914059	0.32	1.28E-06
UU-DU	0.386675752		1.99E-07
UU-UU	0.664410653		4.03E-12
UU-DD	0.784096258	0.72	3.82E-14

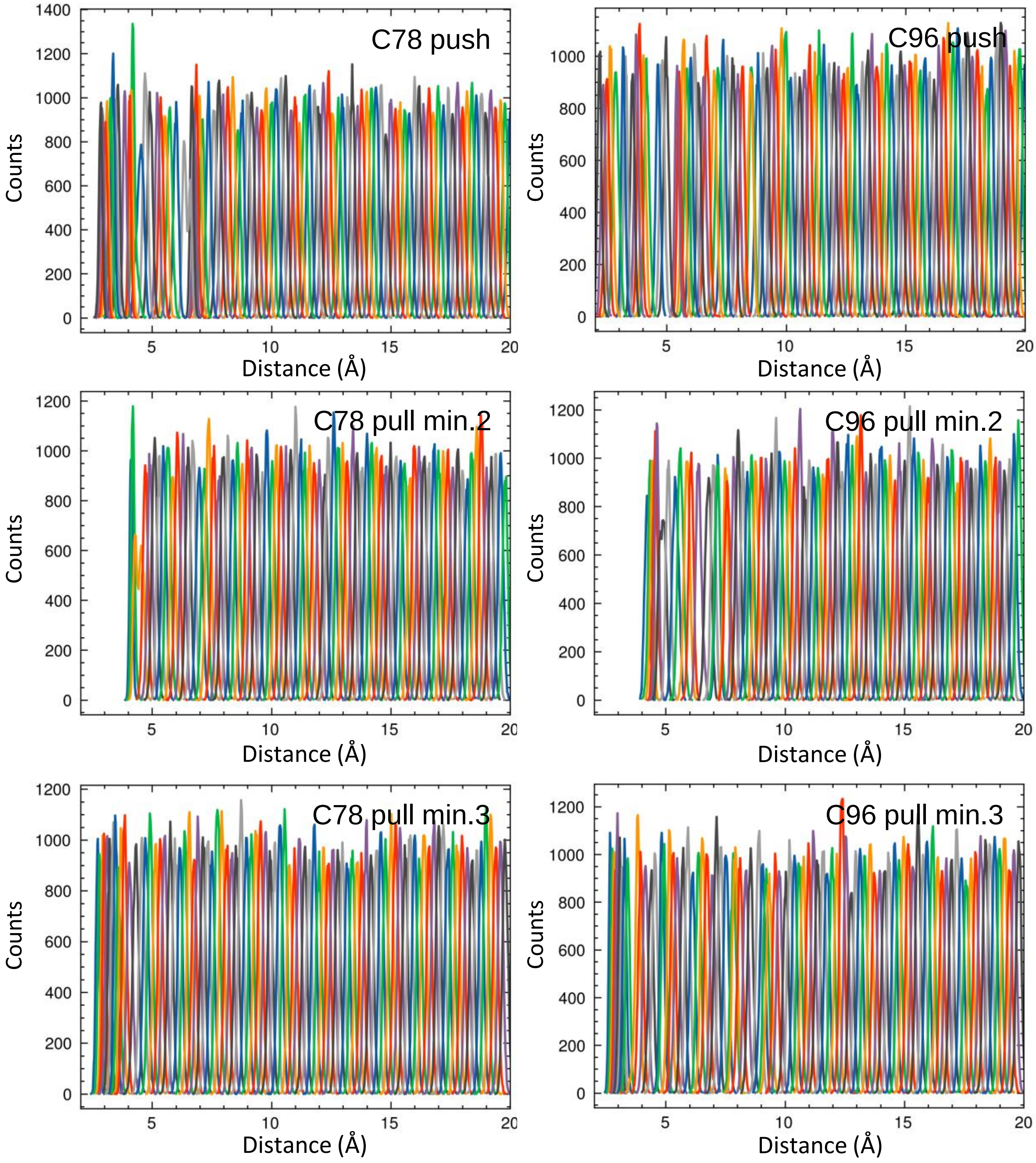

Supplementary Figure 16 | Histogram of the Umbrella Sampling (within each sampling windows) as a function of the reaction coordinate d, that is the distance between the whole GQDs Center Of Mass.

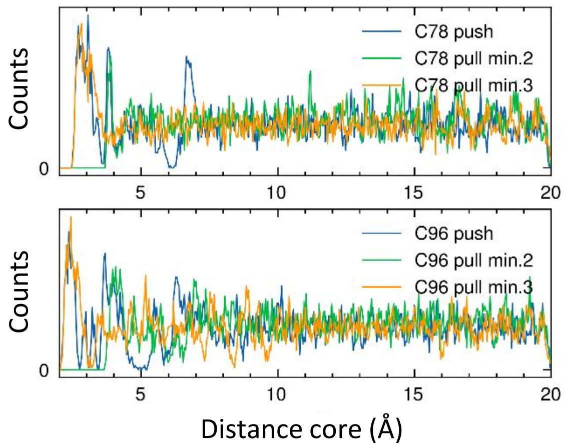

Supplementary Figure 17 | Histogram of the Umbrella sampling (all sampling windows merged) as a function of the Center Of Mass of the GQDs core, i.e. molecule without the *tert*-butyl group.

NMR Spectra (400 MHz)

^{1}H and ^{13}C NMR of (4,4''-di-*tert*-butyl-[1,1':4',1''-terphenyl]-2',5'-diyl)bis(trimethylsilane) (6)

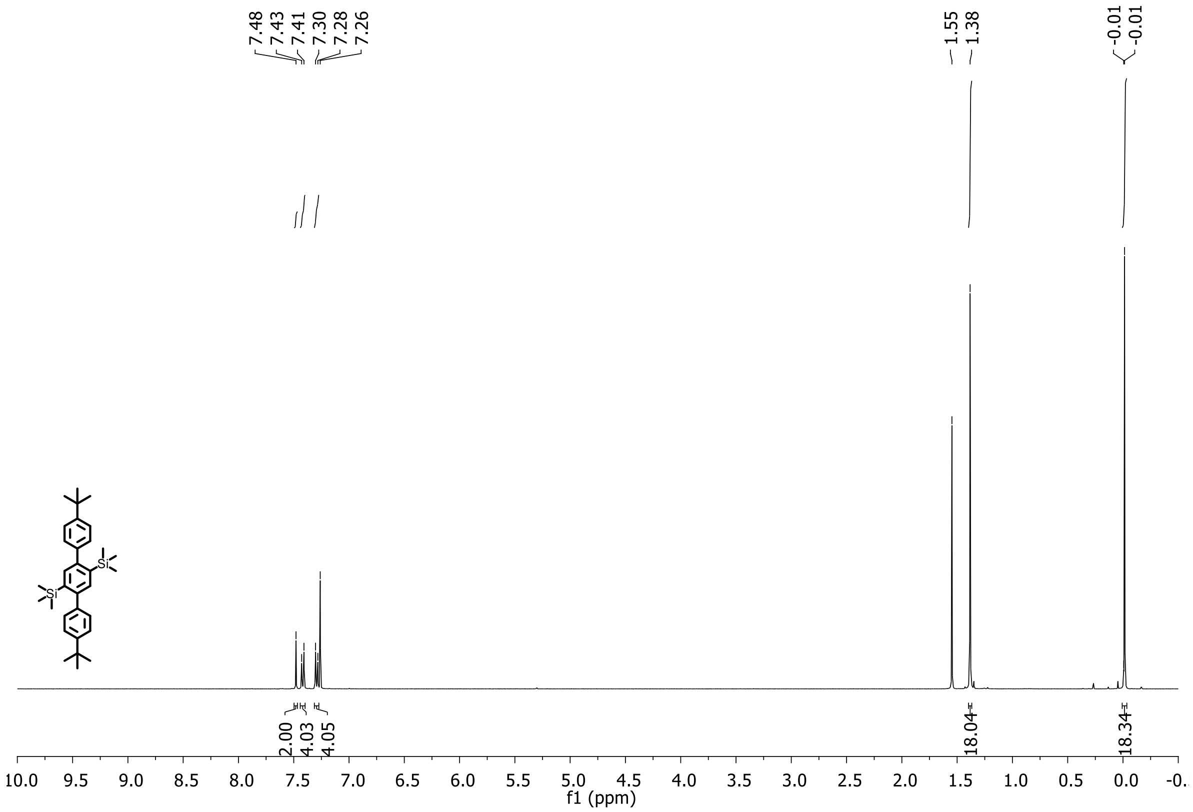

Supplementary Figure 18 | ^{1}H NMR of (4,4''-di-*tert*-butyl-[1,1':4',1''-terphenyl]-2',5'-diyl)bis(trimethylsilane) (**6**).

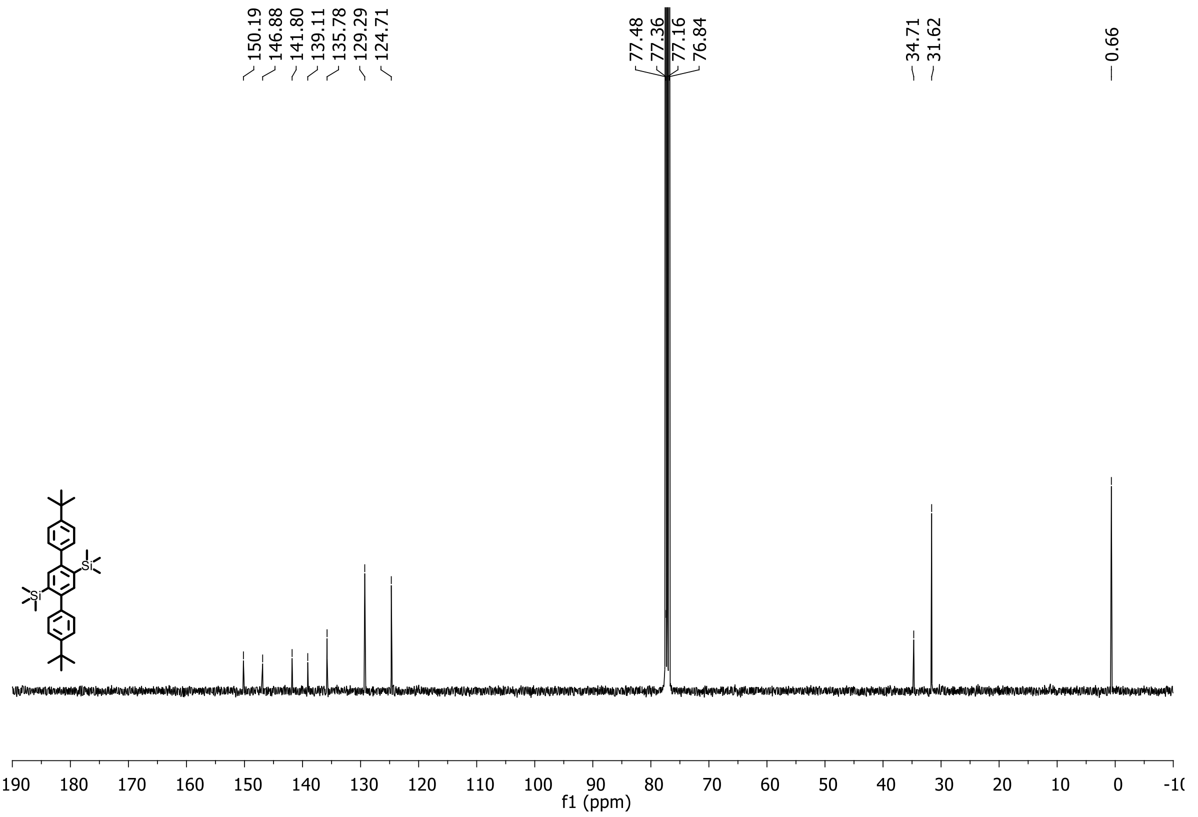

Supplementary Figure 19 | ^{13}C NMR of (4,4''-di-*tert*-butyl-[1,1':4',1''-terphenyl]-2',5'-diyl)bis(trimethylsilane) (**6**).

^{1}H and ^{13}C NMR of 4,4''-di-*tert*-butyl-2',5'-diiodo-1,1':4',1''-terphenyl (7)

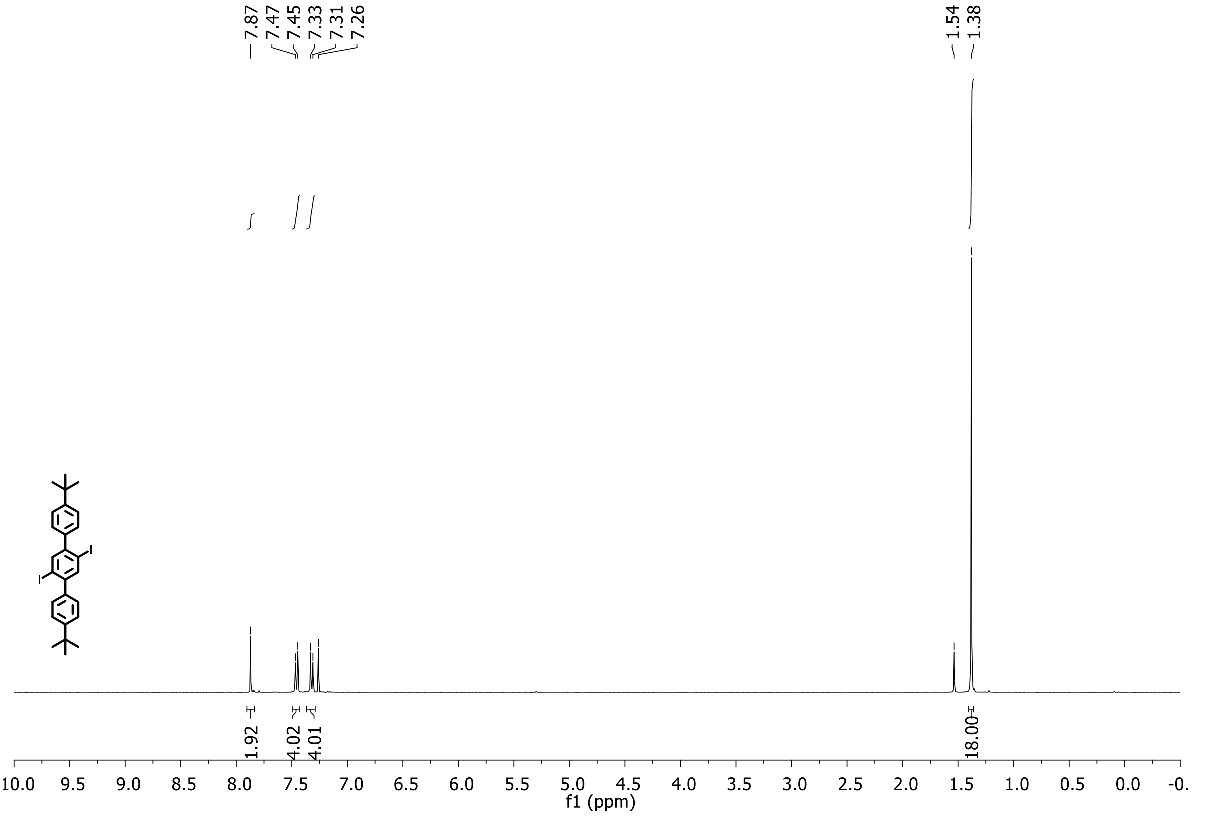

Supplementary Figure 20 | ^{1}H NMR of 4,4''-di-*tert*-butyl-2',5'-diiodo-1,1':4',1''-terphenyl (**7**).

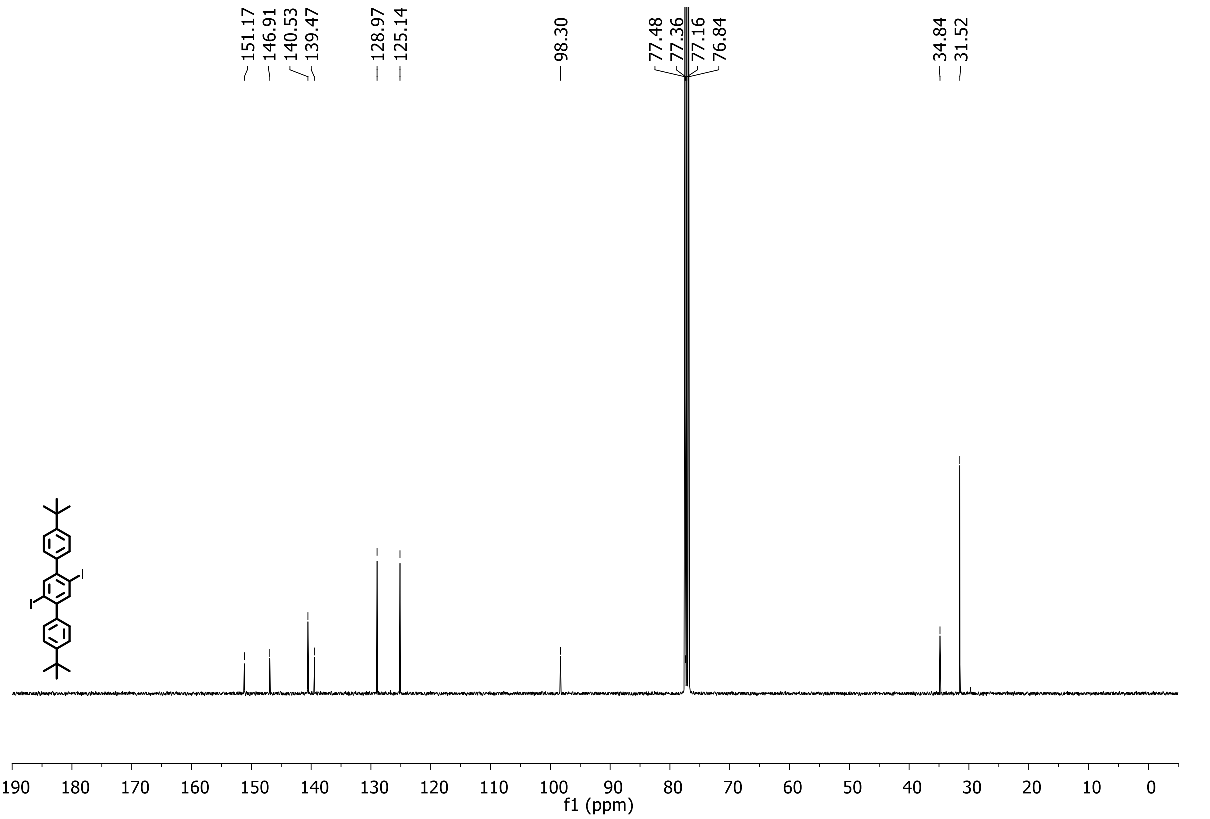

Supplementary Figure 21 | ^{13}C NMR of 4,4''-di-*tert*-butyl-2',5'-diiodo-1,1':4',1''-terphenyl (**7**).

^{1}H and ^{13}C NMR of terphenyl derivative (8)

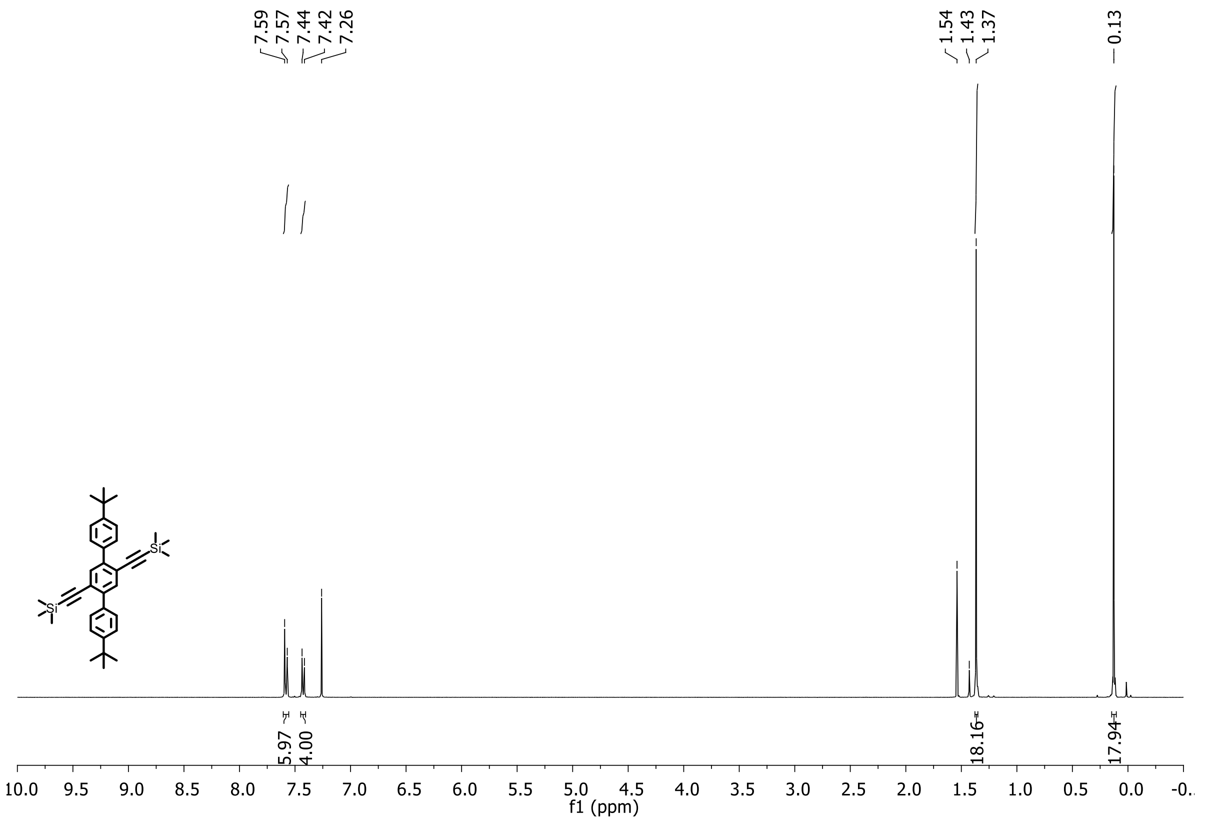

Supplementary Figure 22 | ^{1}H NMR of terphenyl derivative (**8**).

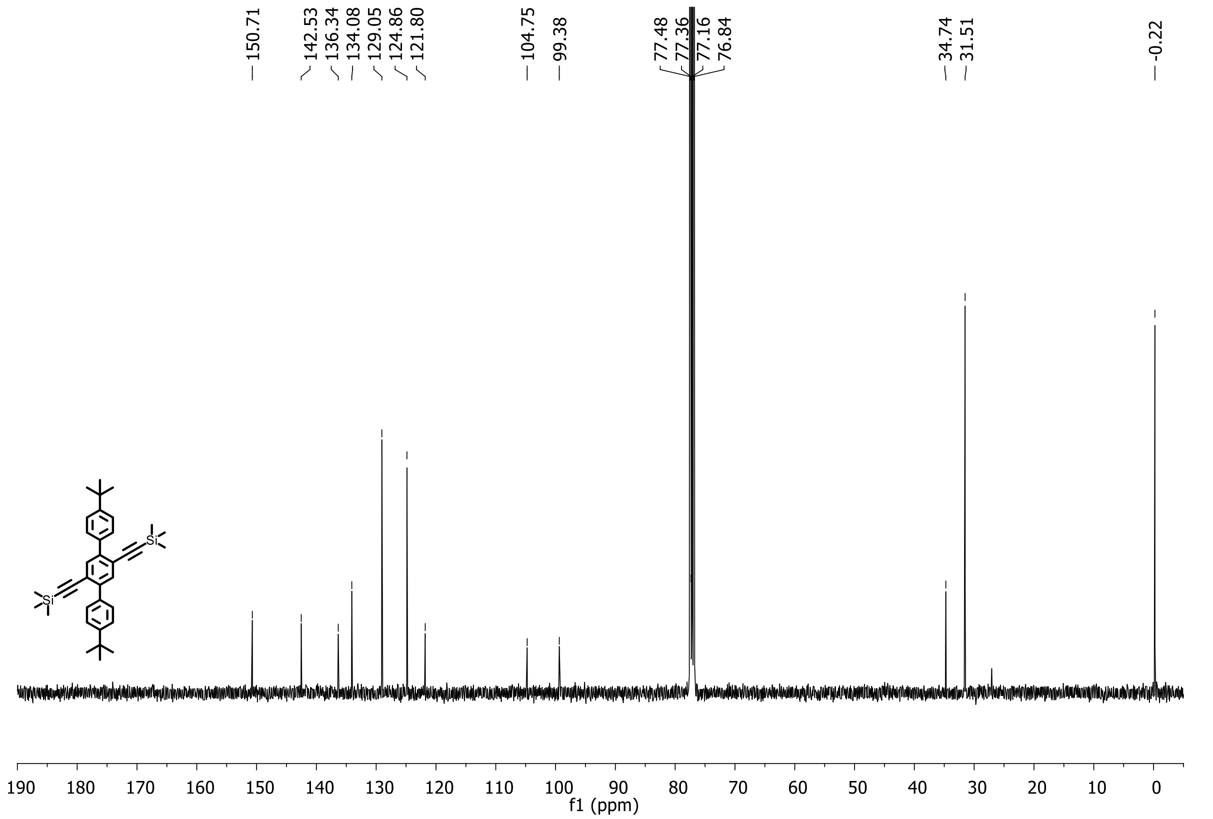

Supplementary Figure 23 | ^{13}C NMR of terphenyl derivative (**8**).

^{1}H and ^{13}C NMR of dendrimer (2)

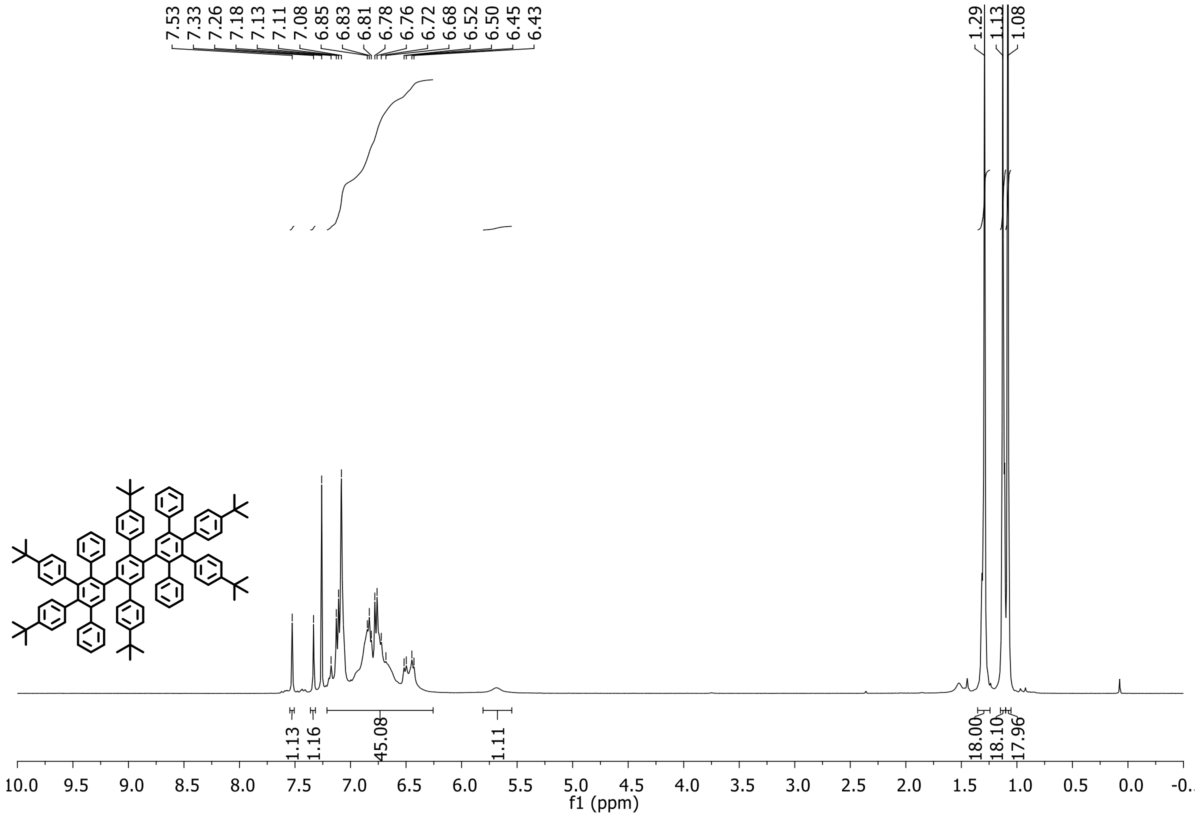

Supplementary Figure 24 | ^{1}H NMR of dendrimer (**2**).

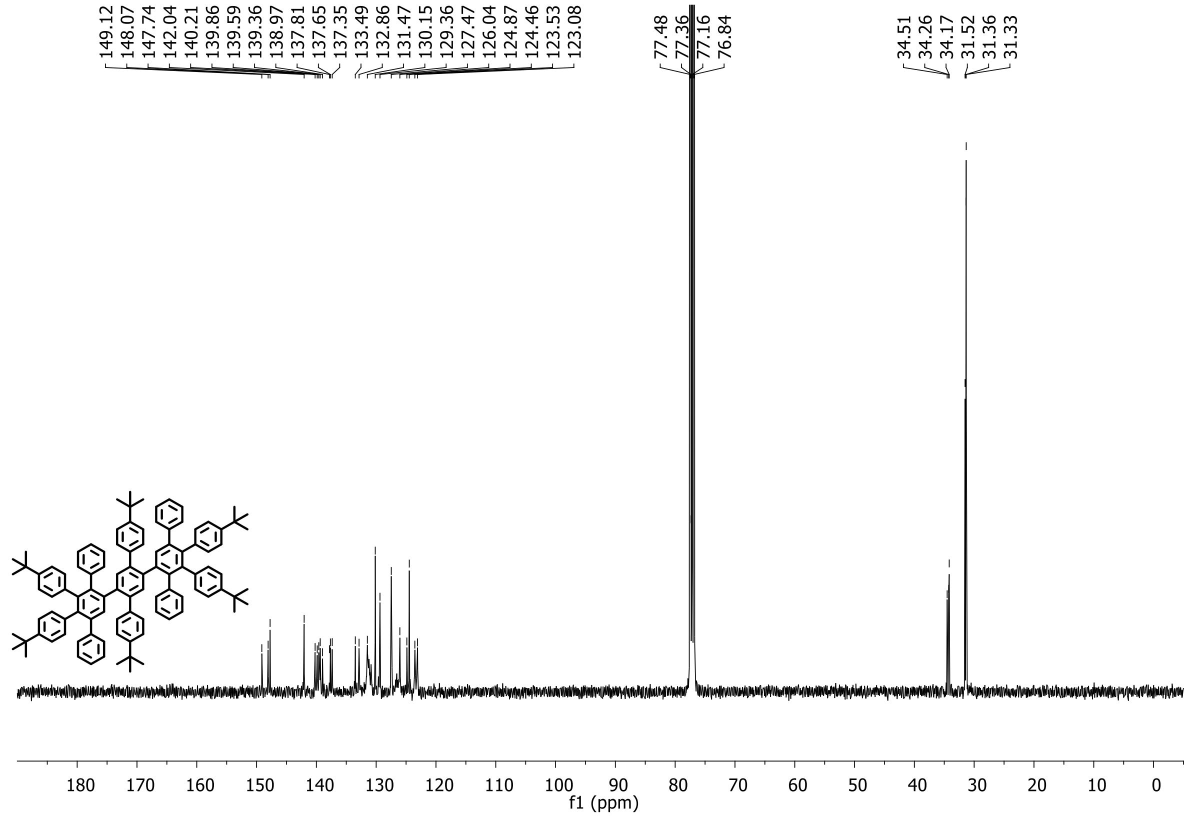

Supplementary Figure 25 | ^{13}C NMR of dendrimer (**2**).

^{1}H and ^{13}C NMR of terphenyl derivative (10)

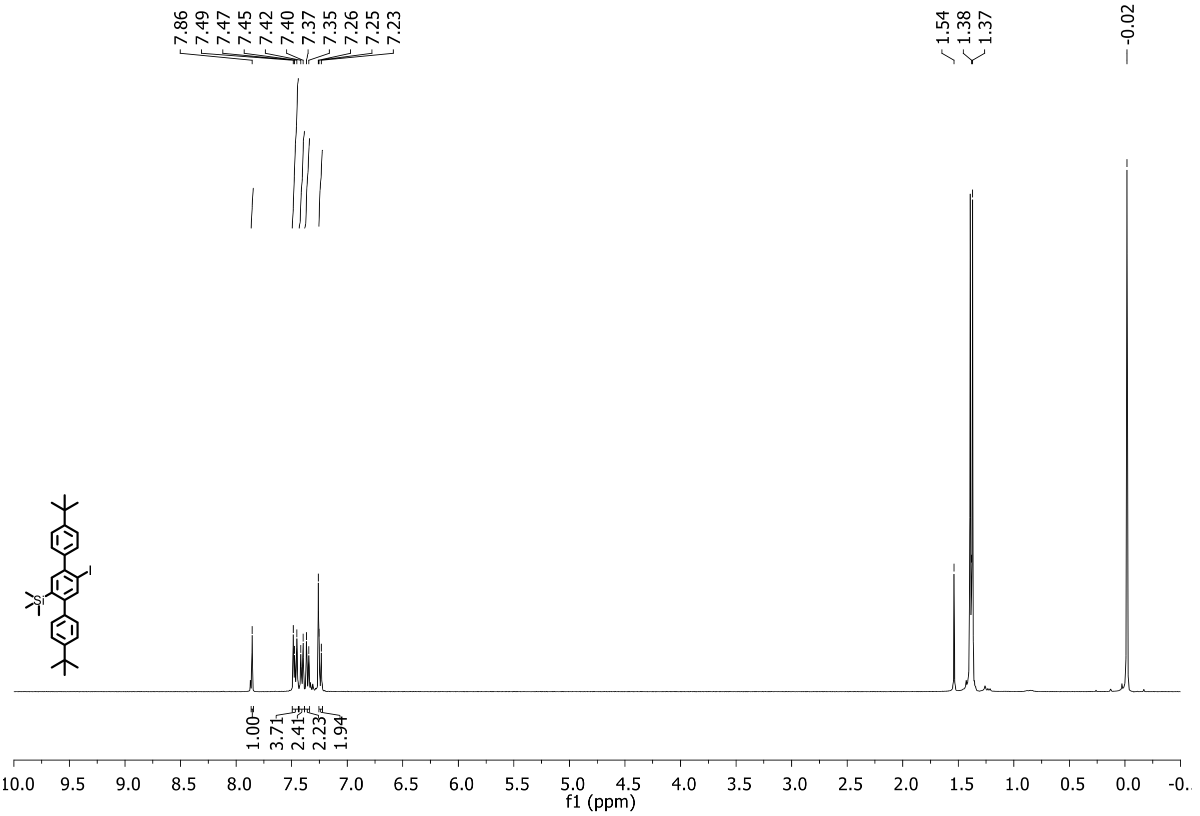

Supplementary Figure 26 | ^{1}H NMR of terphenyl derivative (**10**).

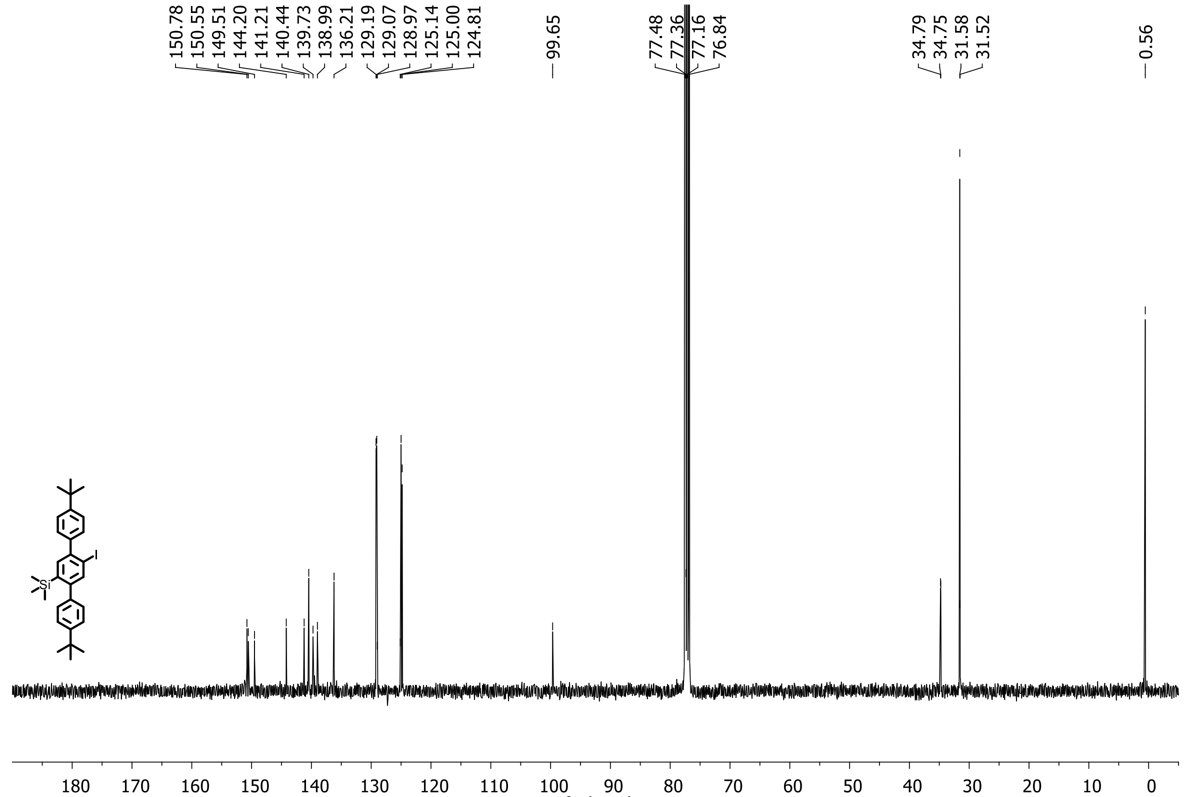

Supplementary Figure 27 | ^{13}C NMR of terphenyl derivative (**10**).

^{1}H and ^{13}C NMR of monoborylated terphenyl derivative (11)

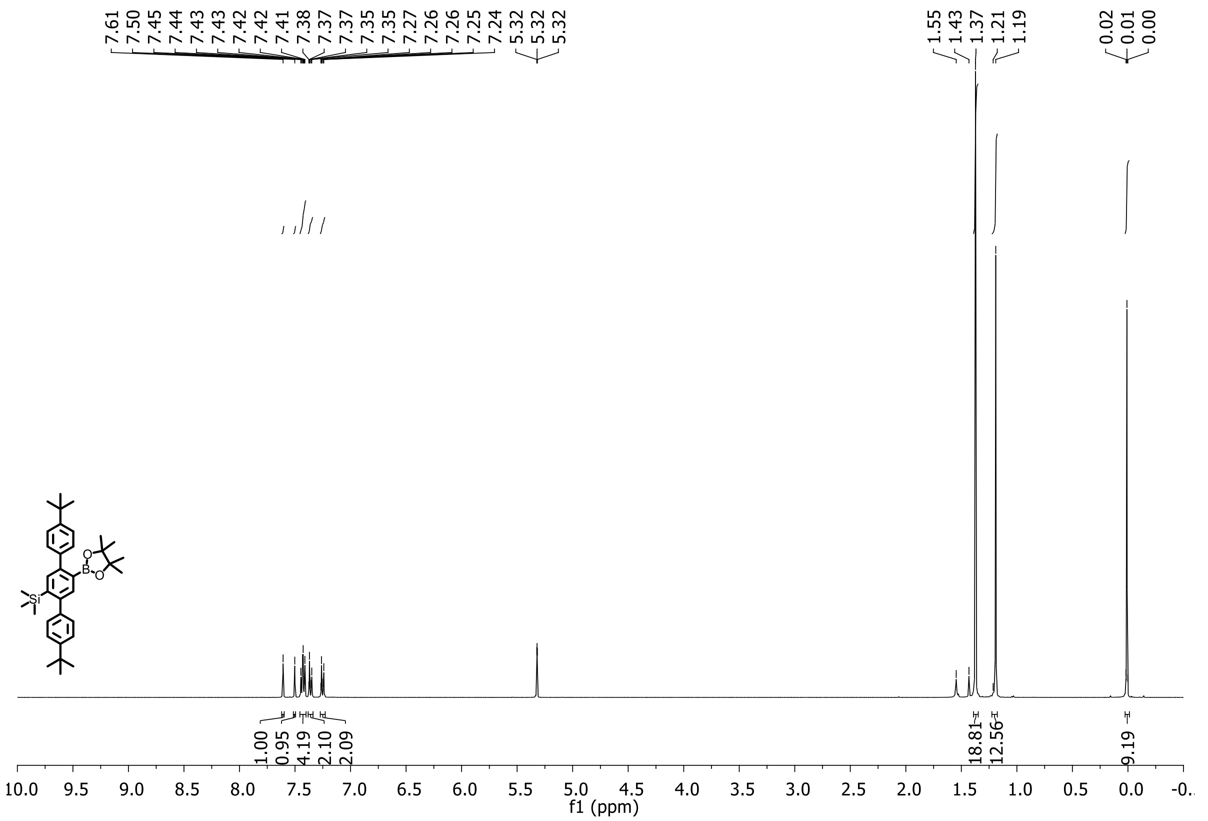

Supplementary Figure 28 | ^{1}H NMR of terphenyl derivative (**11**).

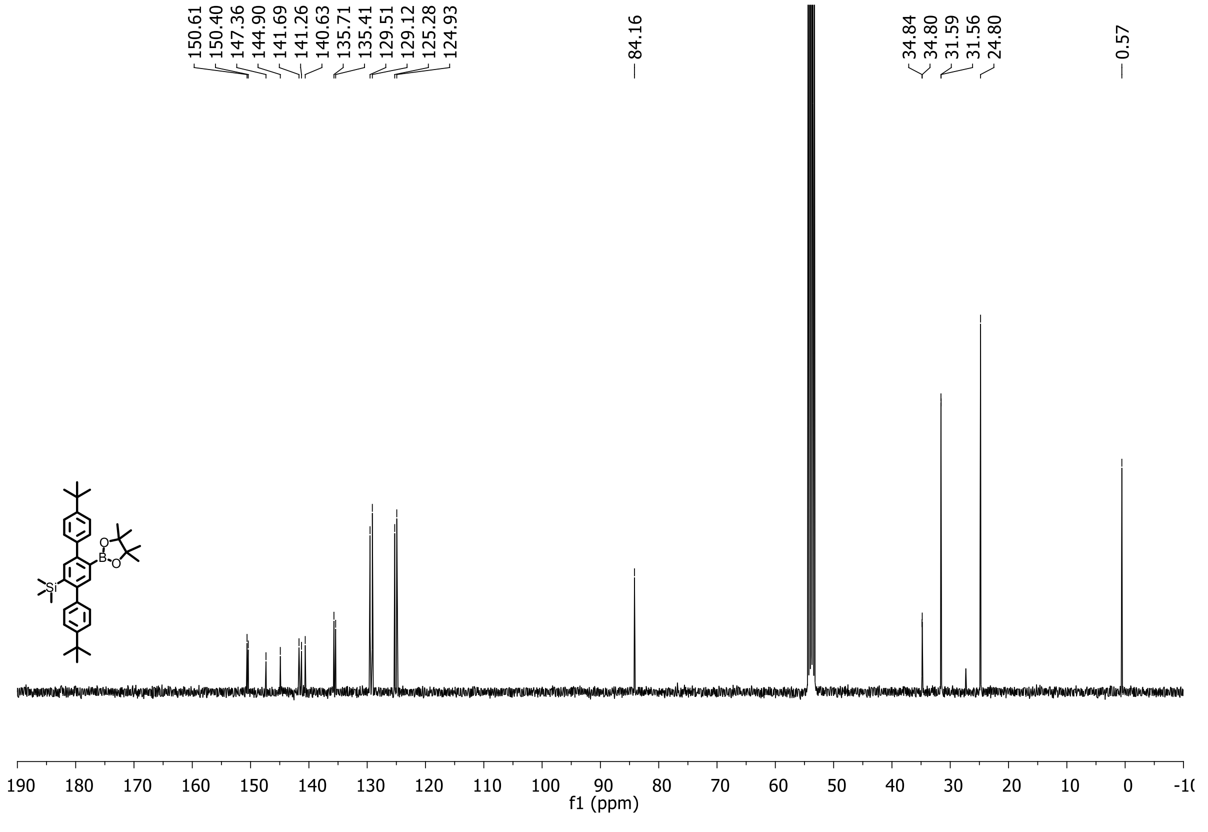

Supplementary Figure 29 | ^{13}C NMR of terphenyl derivative (**11**).

^{1}H and ^{13}C NMR of diterphenyl derivative (12)

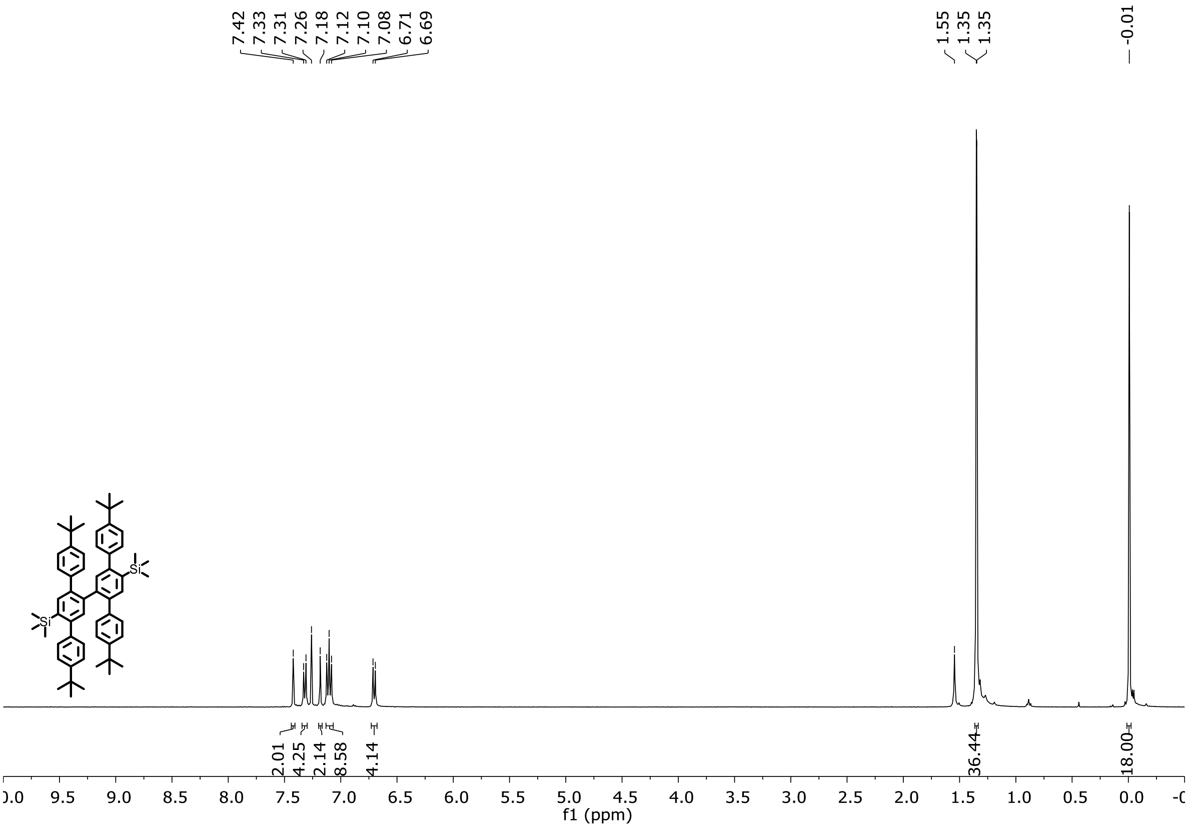

Supplementary Figure 30 | ^{1}H NMR of diterphenyl derivative (**12**).

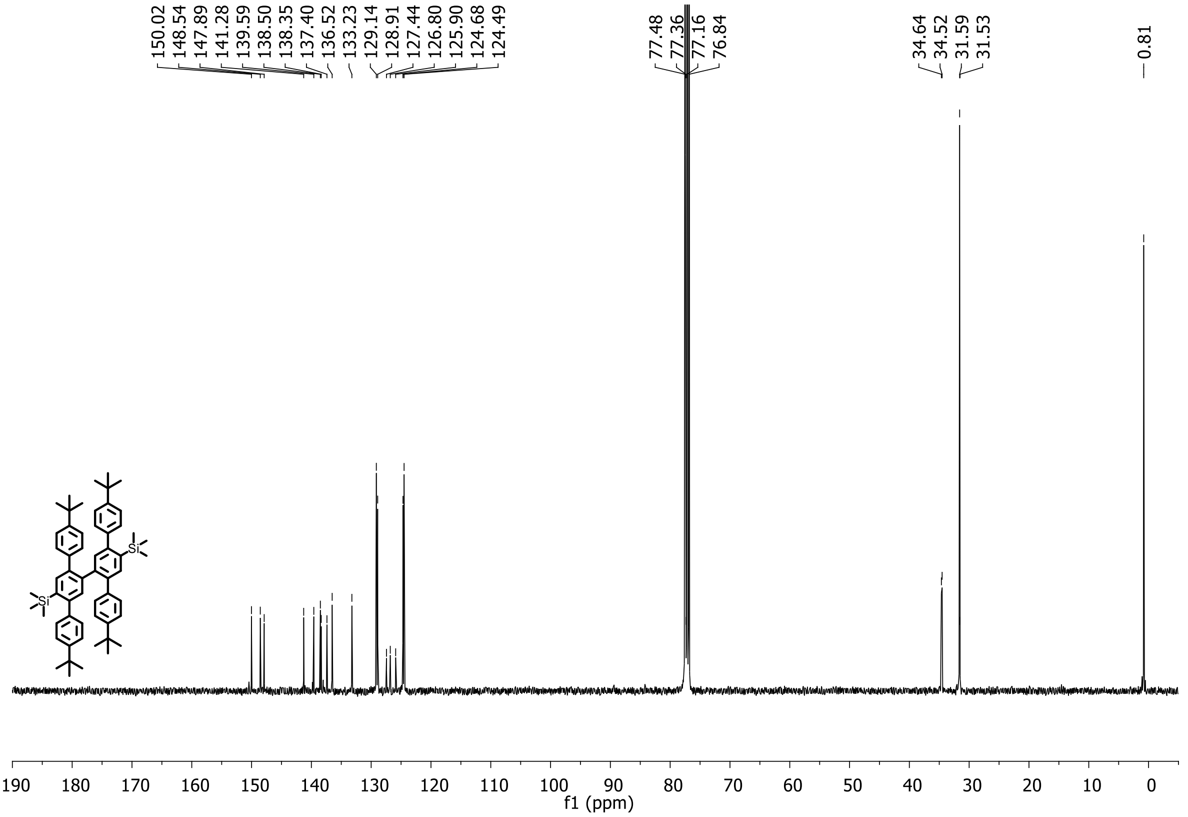

Supplementary Figure 31 | ^{13}C NMR of diterphenyl derivative (**12**).

^{1}H and ^{13}C NMR of diterphenyl derivative (13)

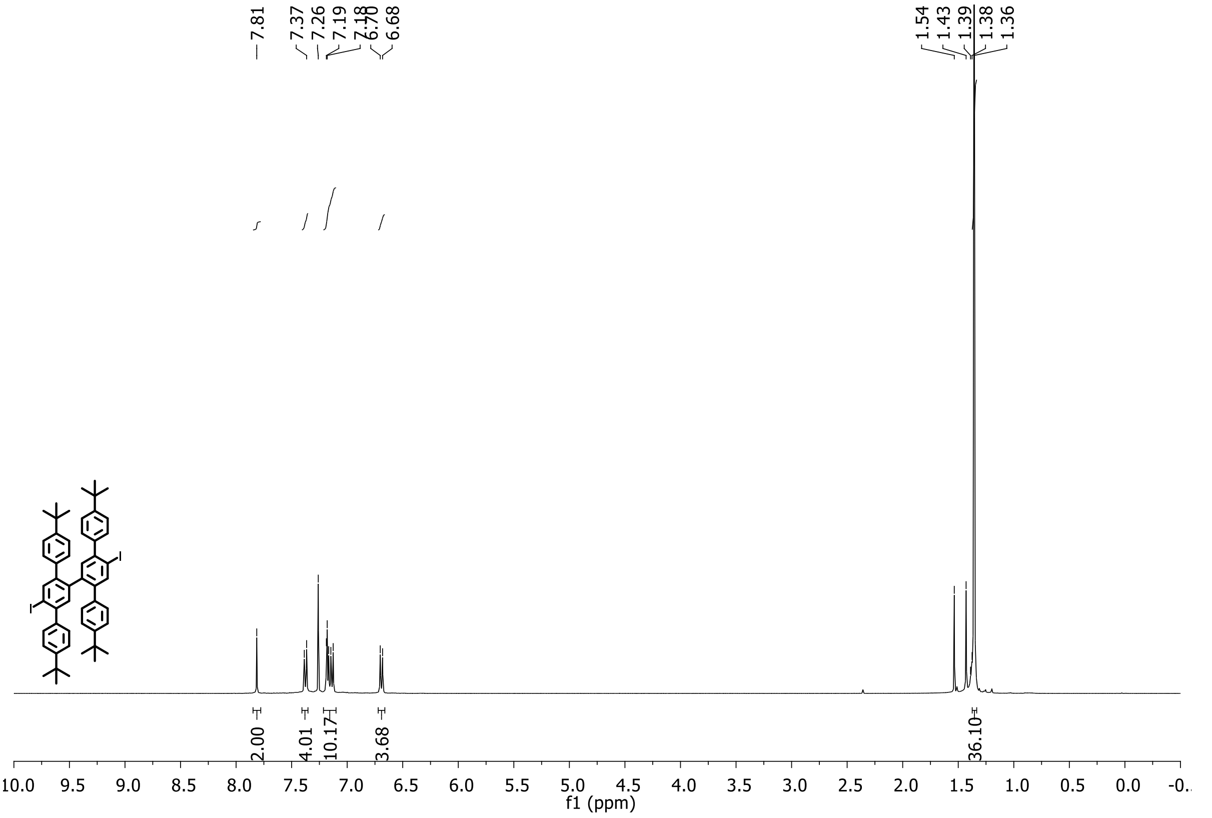

Supplementary Figure 32 | ^{1}H NMR of diterphenyl derivative (**13**).

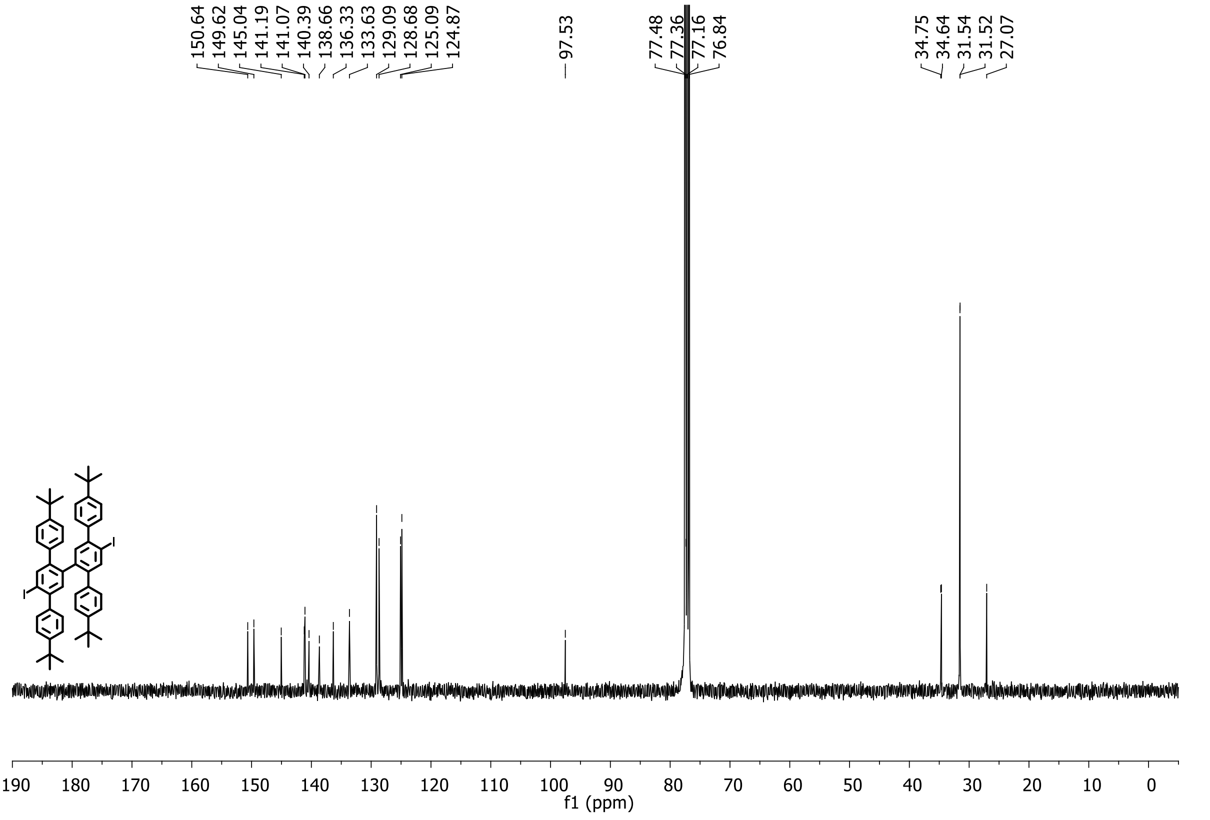

Supplementary Figure 33 | ^{13}C NMR of diterphenyl derivative (**13**).

^{1}H and ^{13}C NMR of diterphenyl derivative (14)

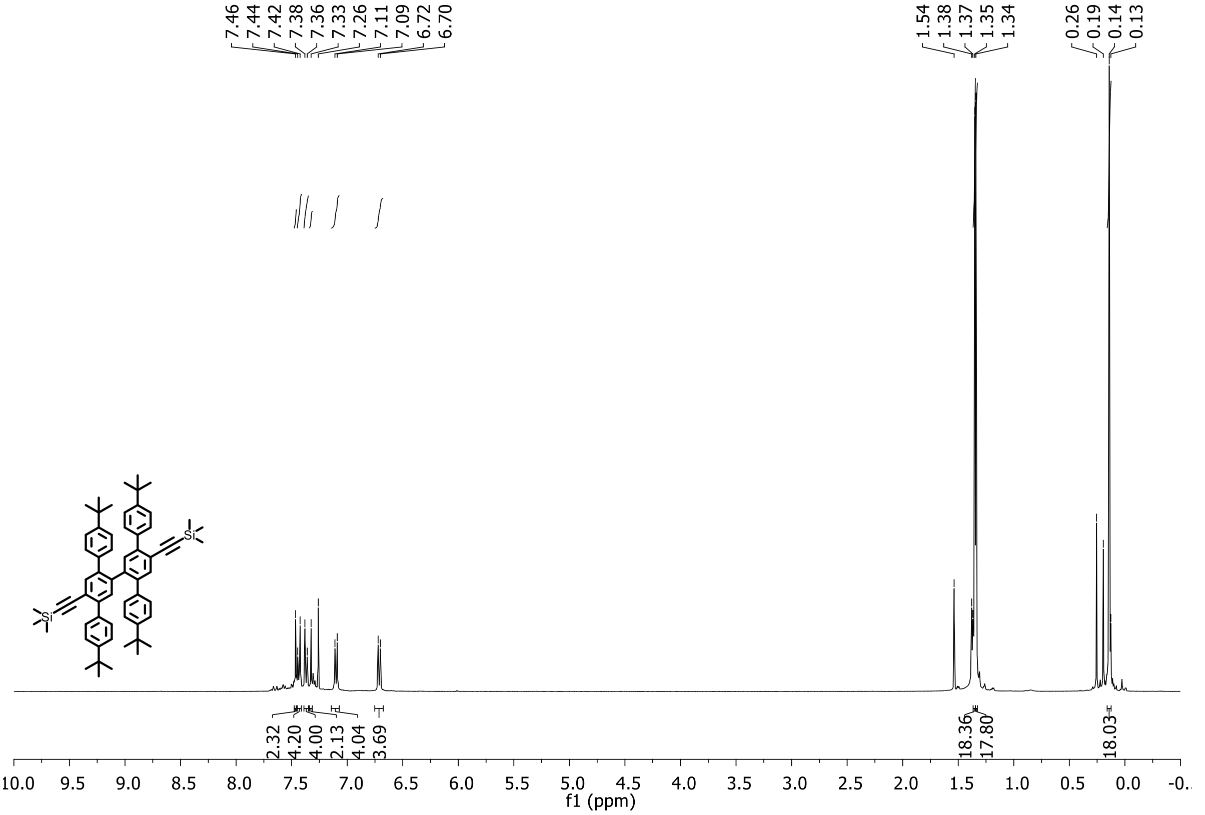

Supplementary Figure 34 | ^{1}H NMR of diterphenyl derivative (**14**).

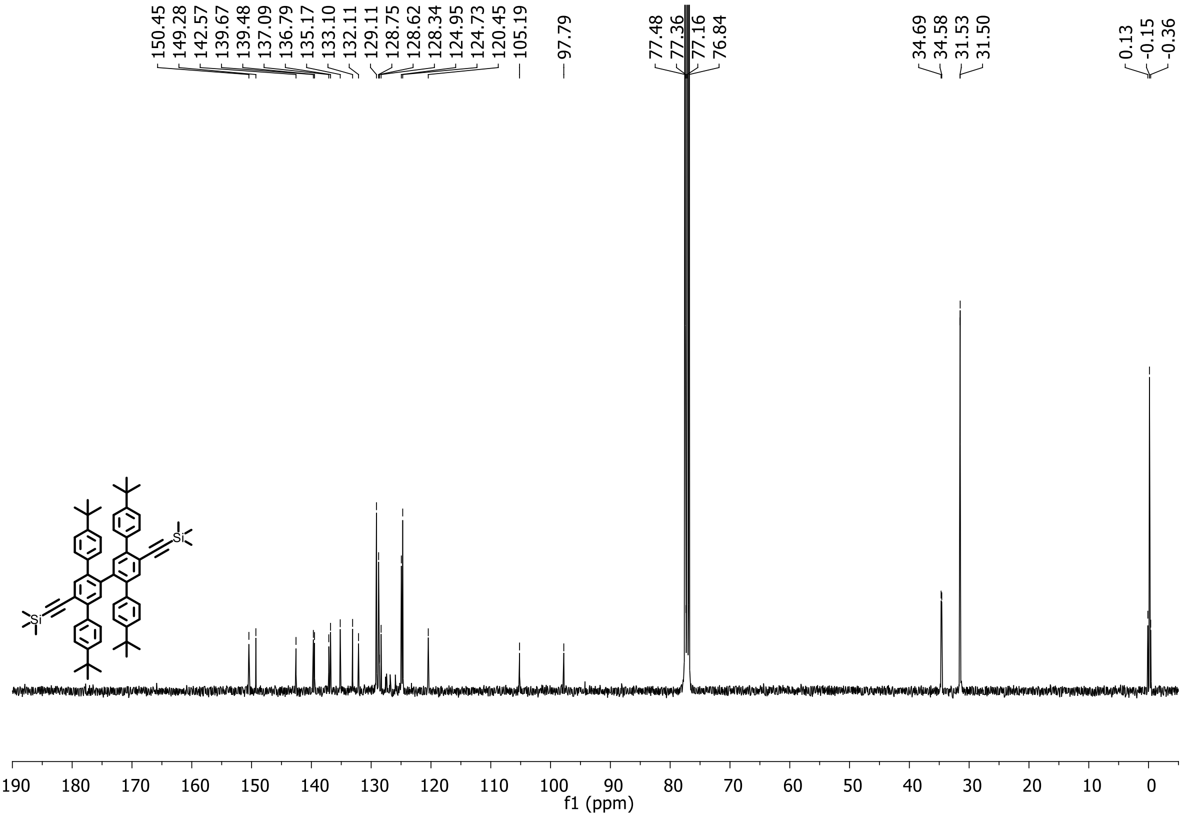

Supplementary Figure 35 | ^{13}C NMR of diterphenyl derivative (**14**).

^{1}H and ^{13}C NMR of dendrimer (1)

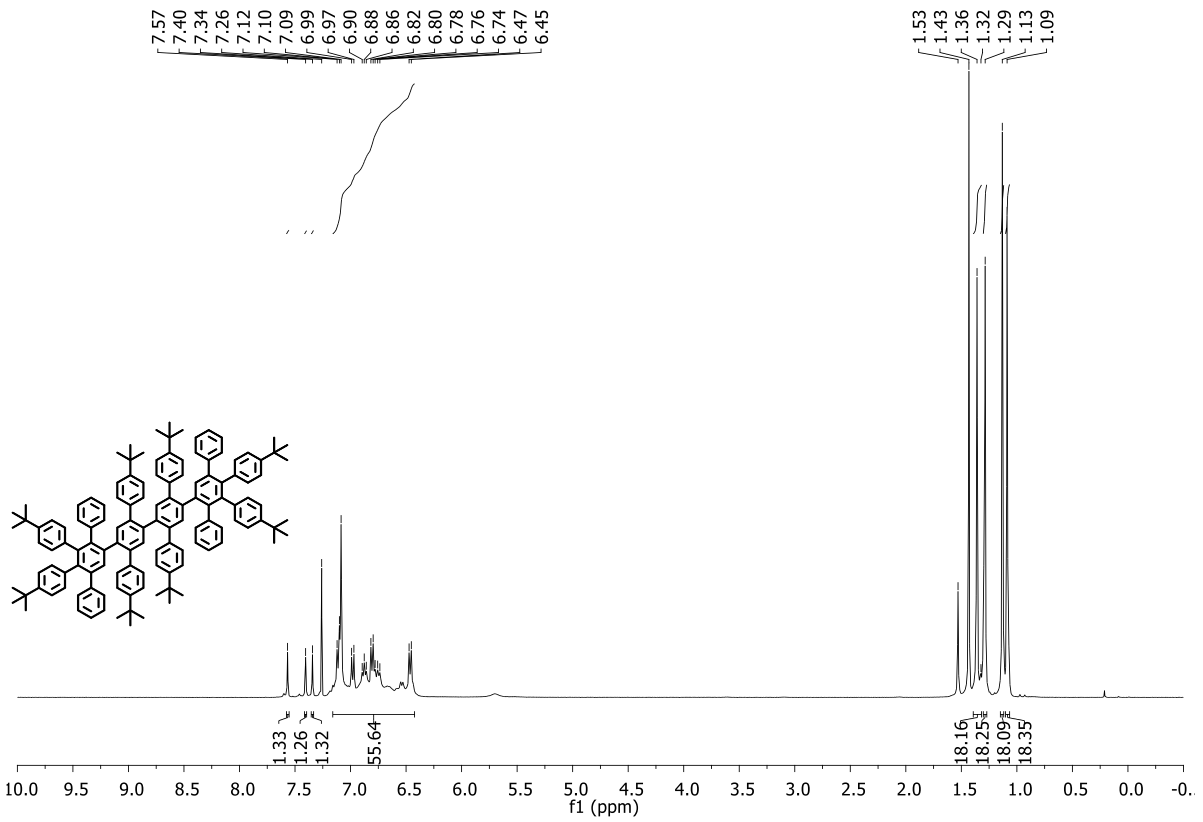

Supplementary Figure 36 | ^{1}H NMR of dendrimer (**1**).

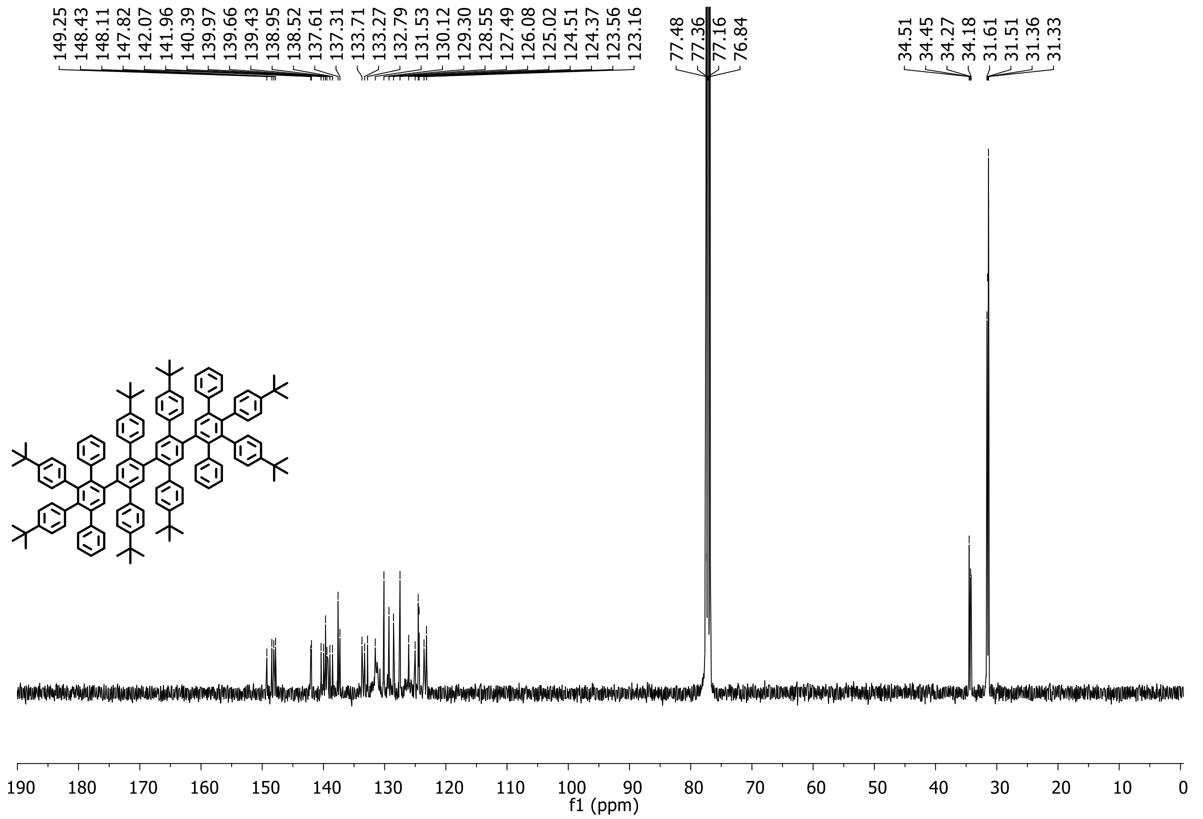

Supplementary Figure 37 | ^{13}C NMR of dendrimer (**1**).

^{1}H and ^{13}C NMR of triterphenyl derivative (15)

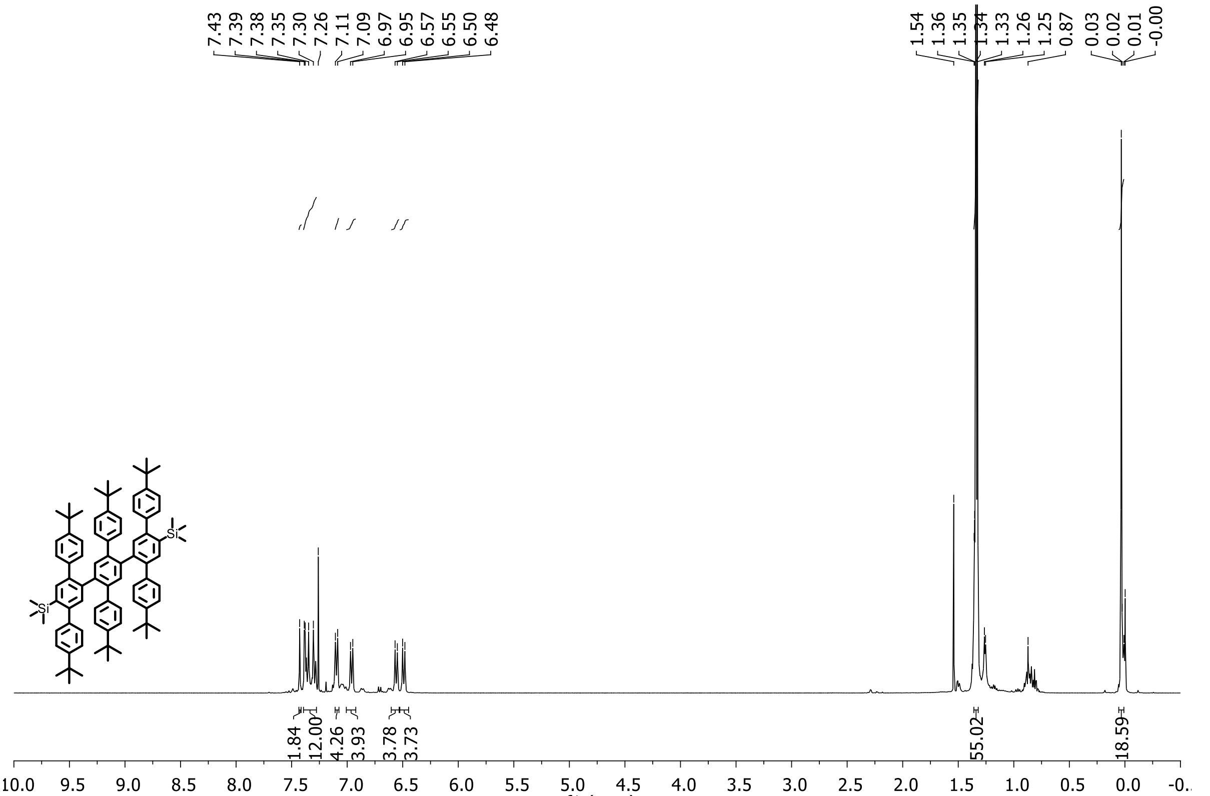

Supplementary Figure 38 | ^{1}H NMR of triterphenyl derivative (**15**).

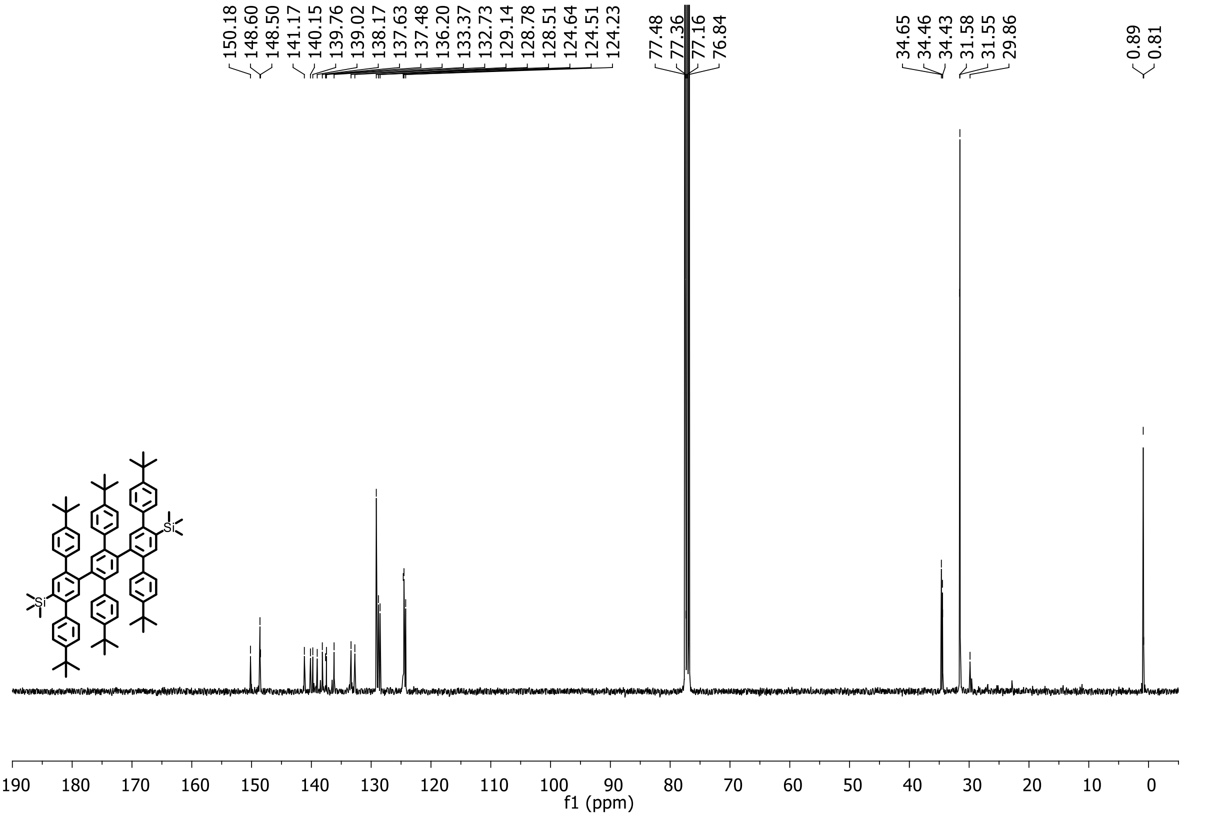

Supplementary Figure 39 | ^{13}C NMR of triterphenyl derivative (**15**).

^{1}H and ^{13}C NMR of triterphenyl derivative (16)

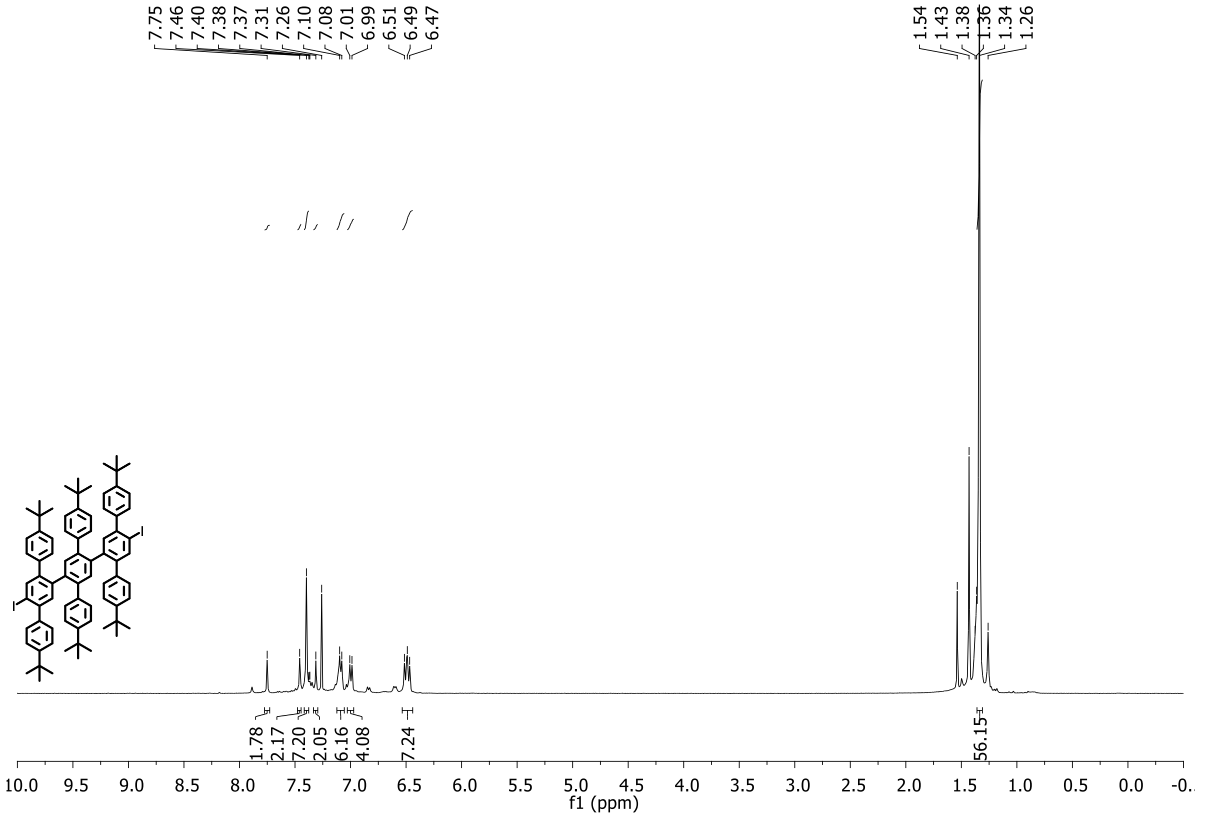

Supplementary Figure 40 | ^{1}H NMR of triterphenyl derivative (**16**).

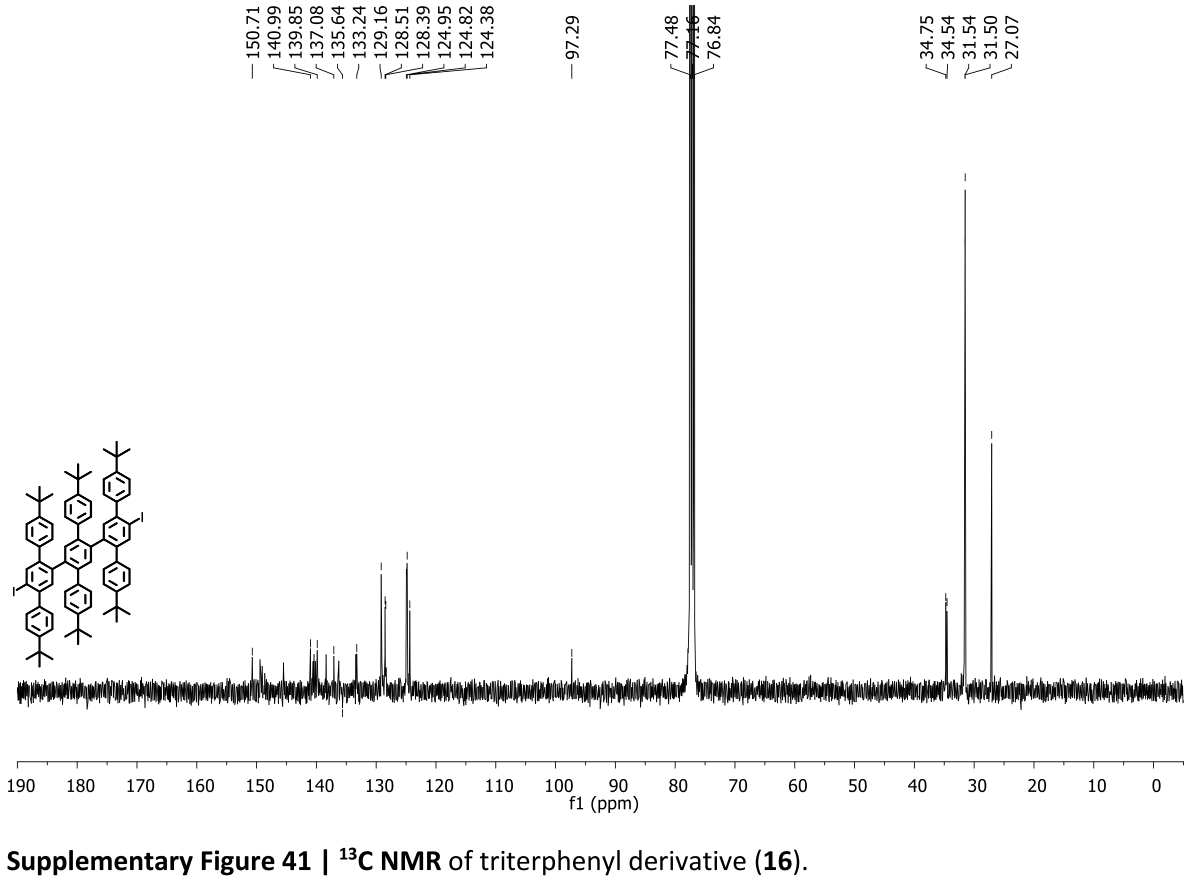

Supplementary Figure 41 | ^{13}C NMR of triterphenyl derivative (**16**).

^{1}H and ^{13}C NMR of triterphenyl derivative (17)

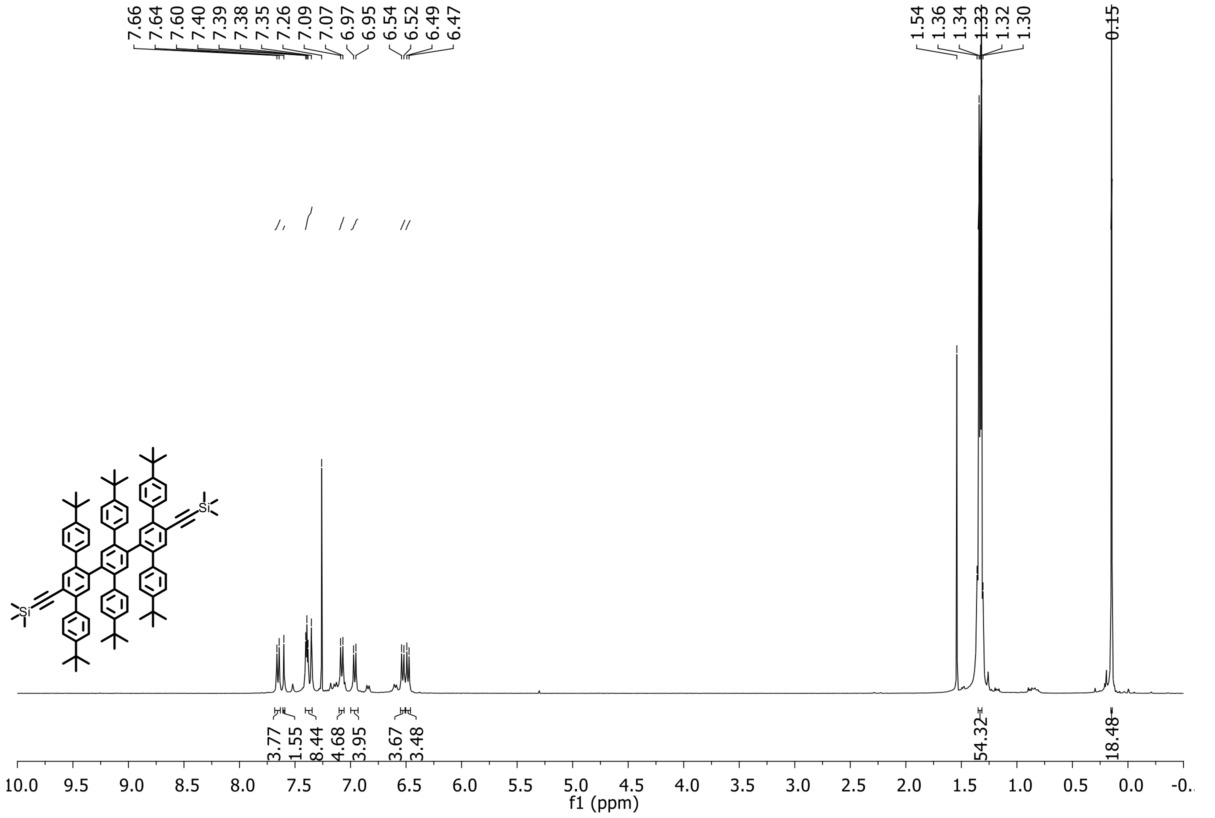

Supplementary Figure 42 | ^{1}H NMR of triterphenyl derivative (**17**).

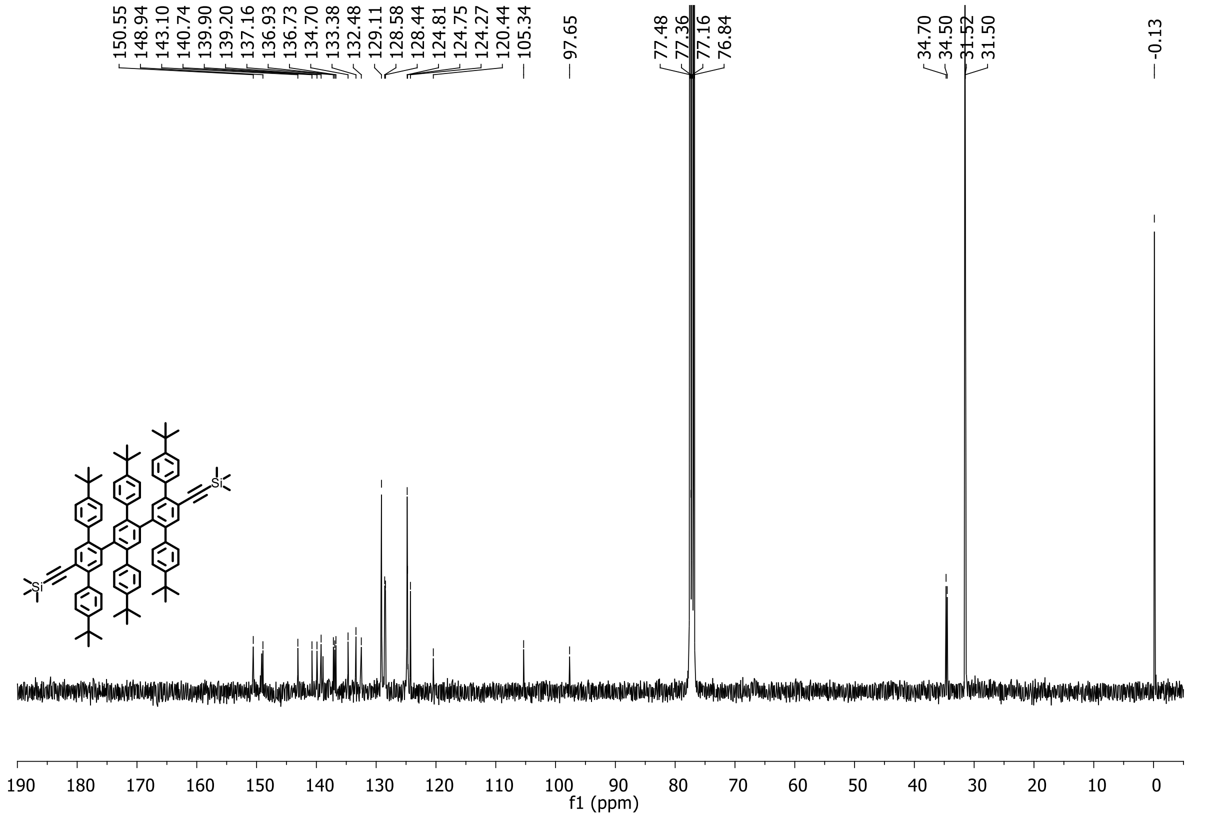

Supplementary Figure 43 | ^{13}C NMR of triterphenyl derivative (**17**).

^{1}H and ^{13}C NMR of dendrimer (3)

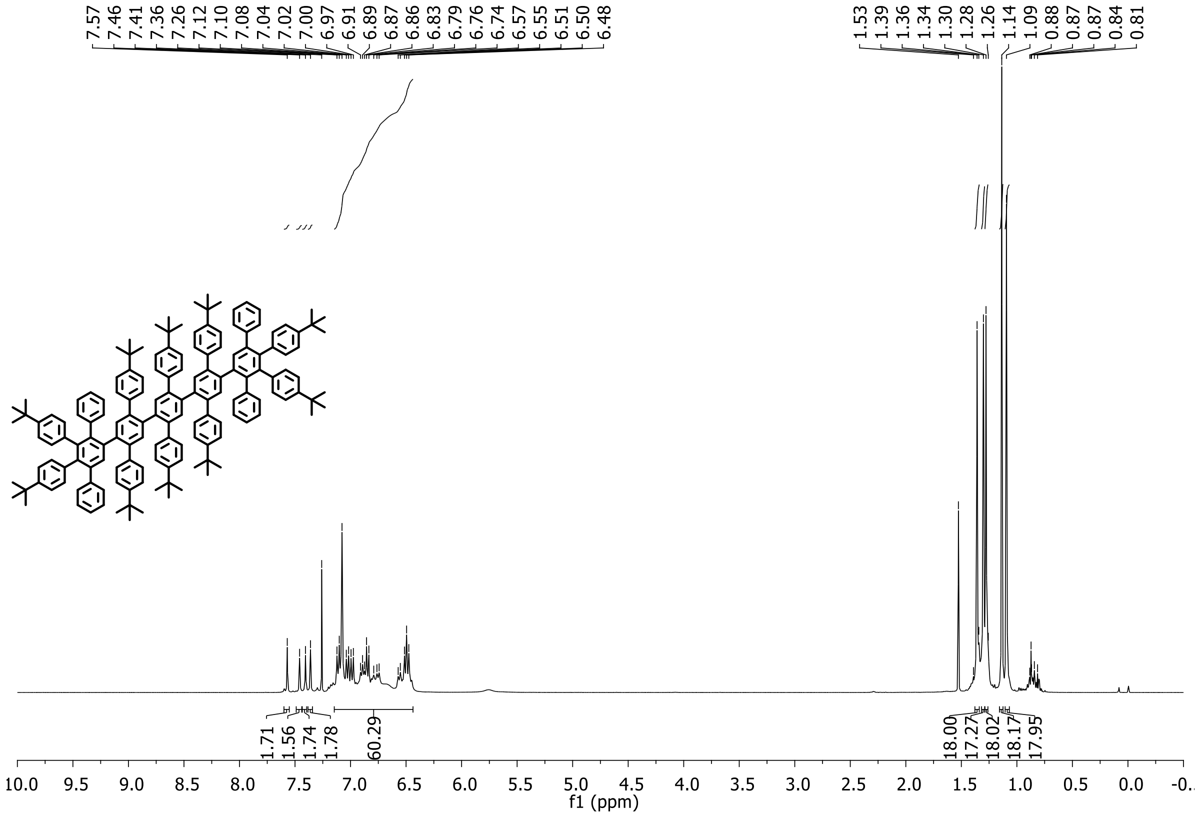

Supplementary Figure 44 | ^{1}H NMR of dendrimer (**3**).

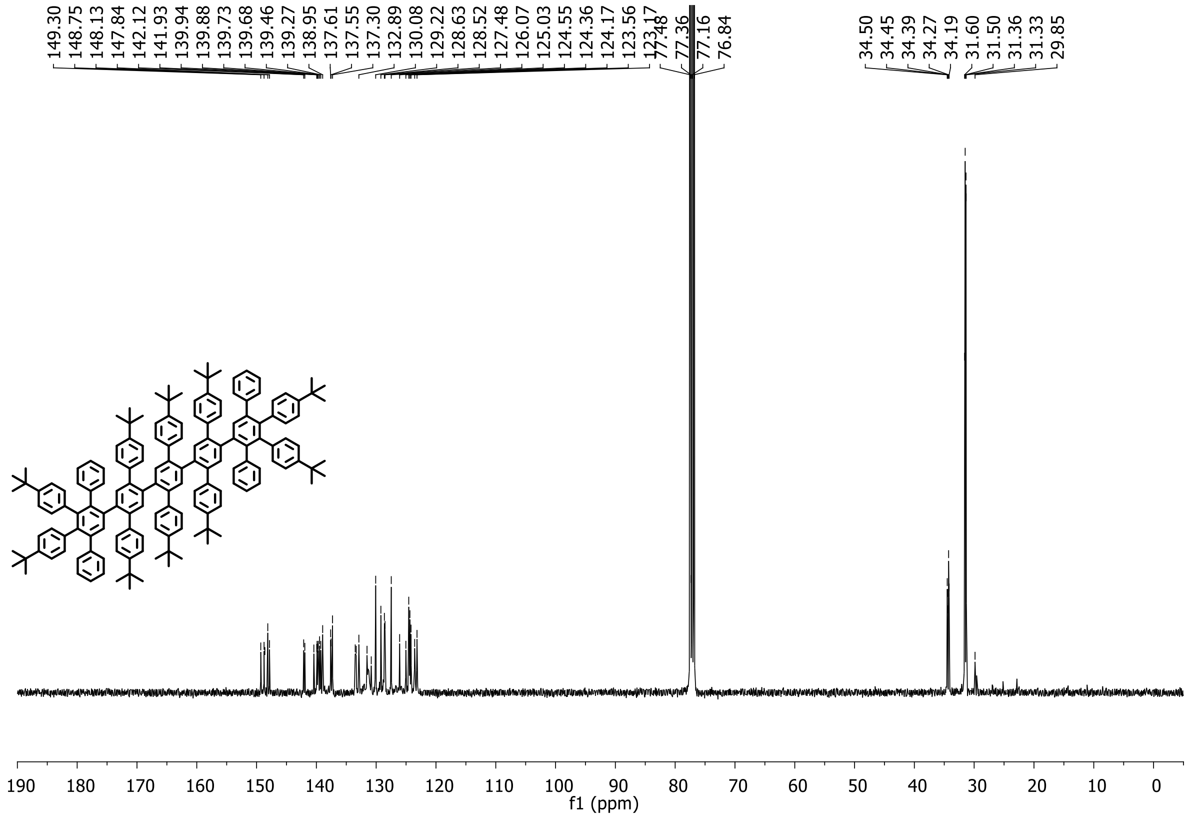

Supplementary Figure 45 | ^{13}C NMR of dendrimer (**3**).

^{1}H and ^{13}C NMR of ditrimetylsilyl tetraterphenyl derivative (18).

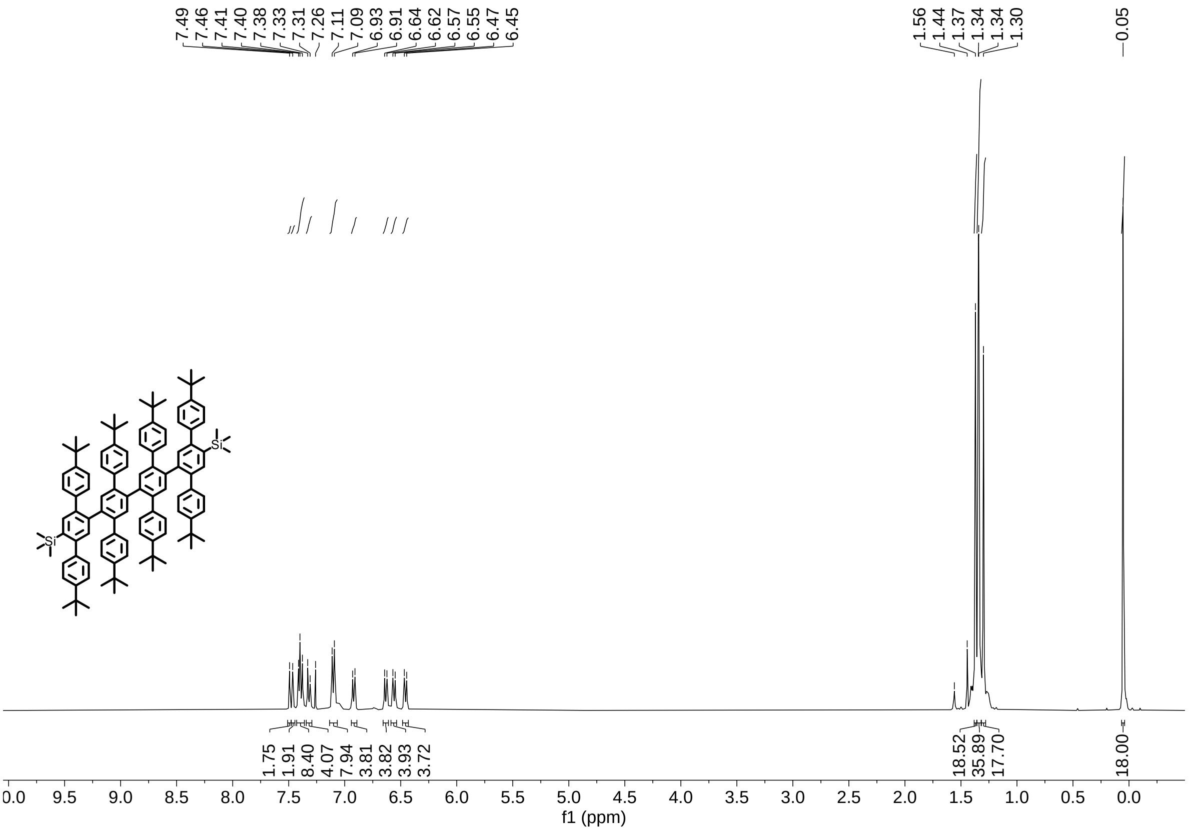

Supplementary Figure 46 | ^{1}H NMR of tetraterphenyl derivative (**18**).

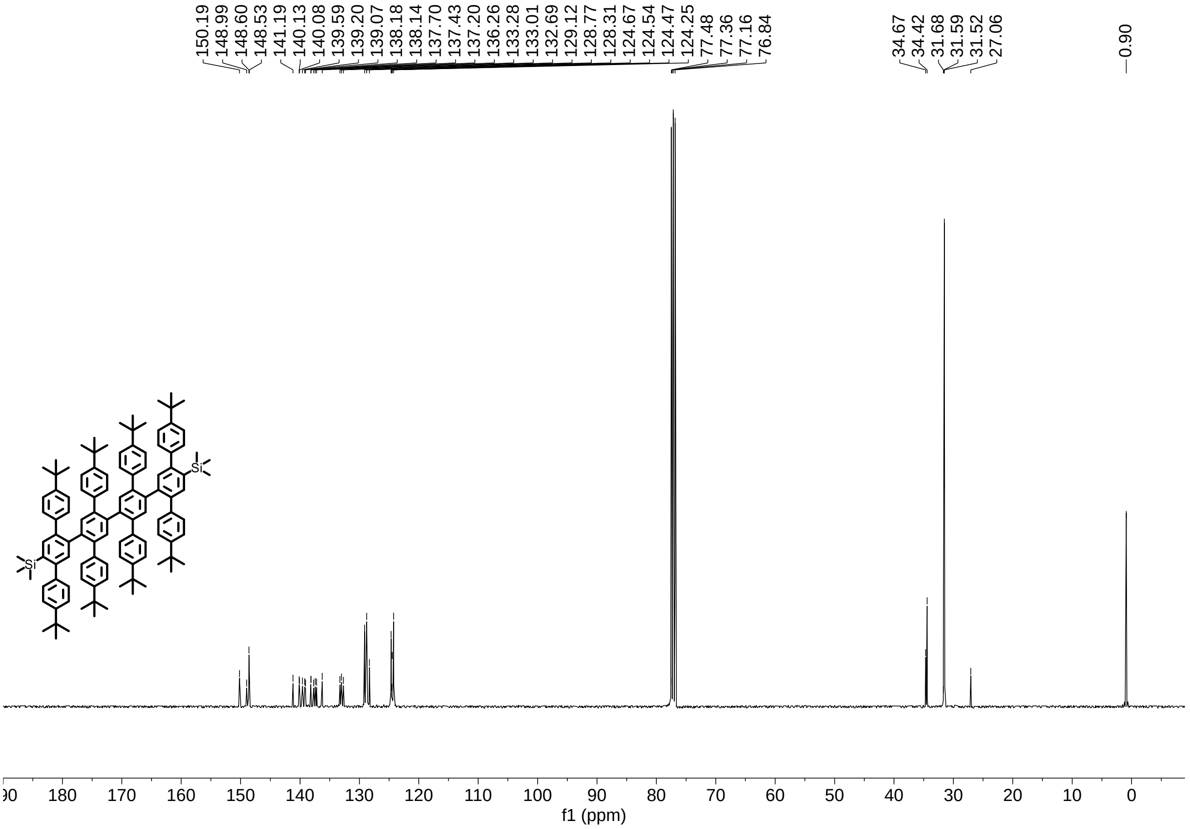

Supplementary Figure 47 | ^{13}C NMR of tetraterphenyl derivative (**18**).

^{1}H and ^{13}C NMR of diiodo tetraterpheyl derivative (19).

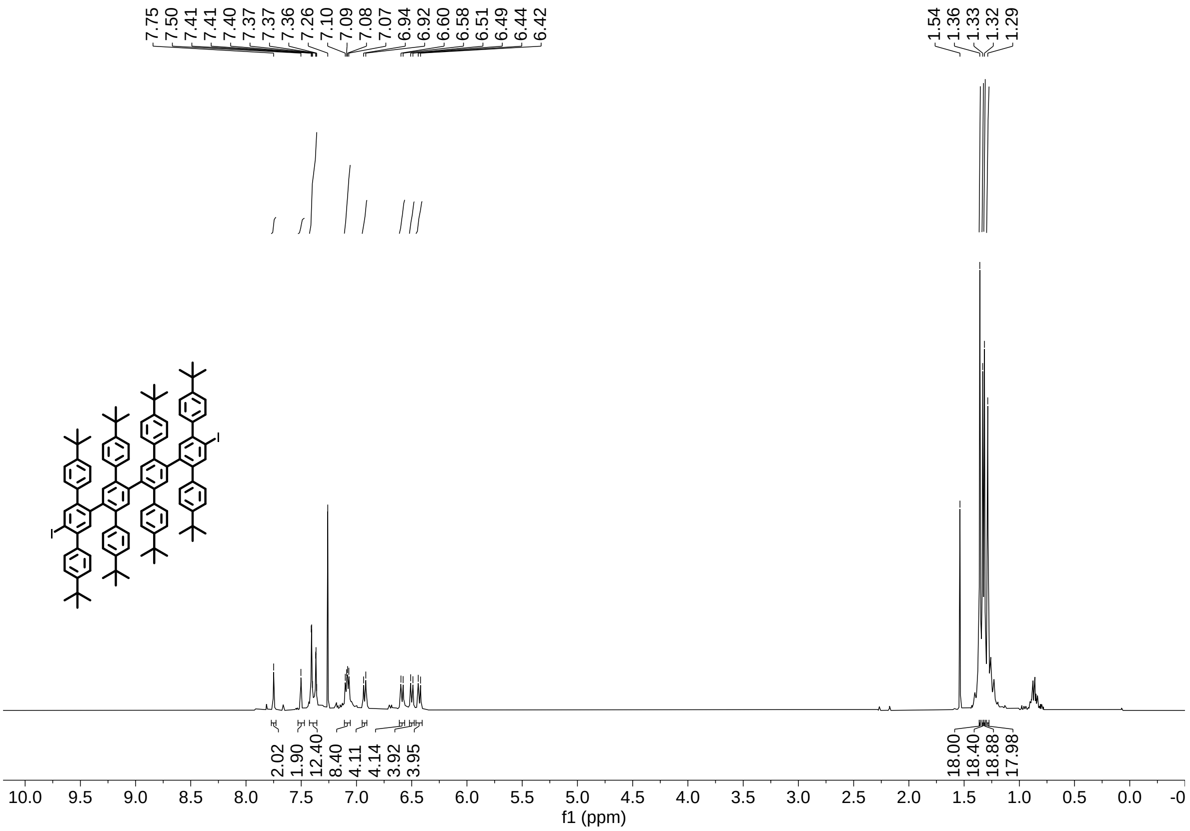

Supplementary Figure 46 | ^{1}H NMR of tetraterphenyl derivative (**19**).

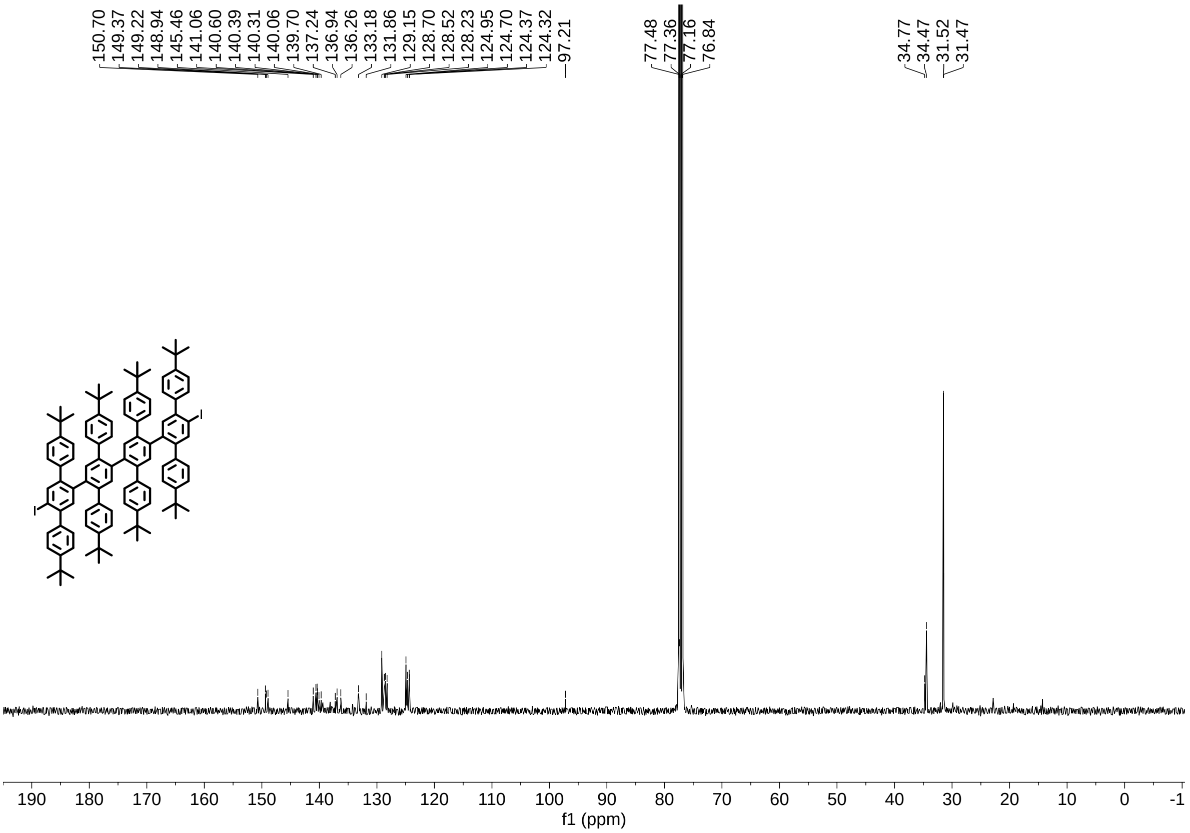

Supplementary Figure 49 | ^{13}C NMR of tetraterphenyl derivative (**19**).

^{1}H and ^{13}C NMR of di-(trimethylsilyl)-ethynyl tetraterphenyl derivative (20).

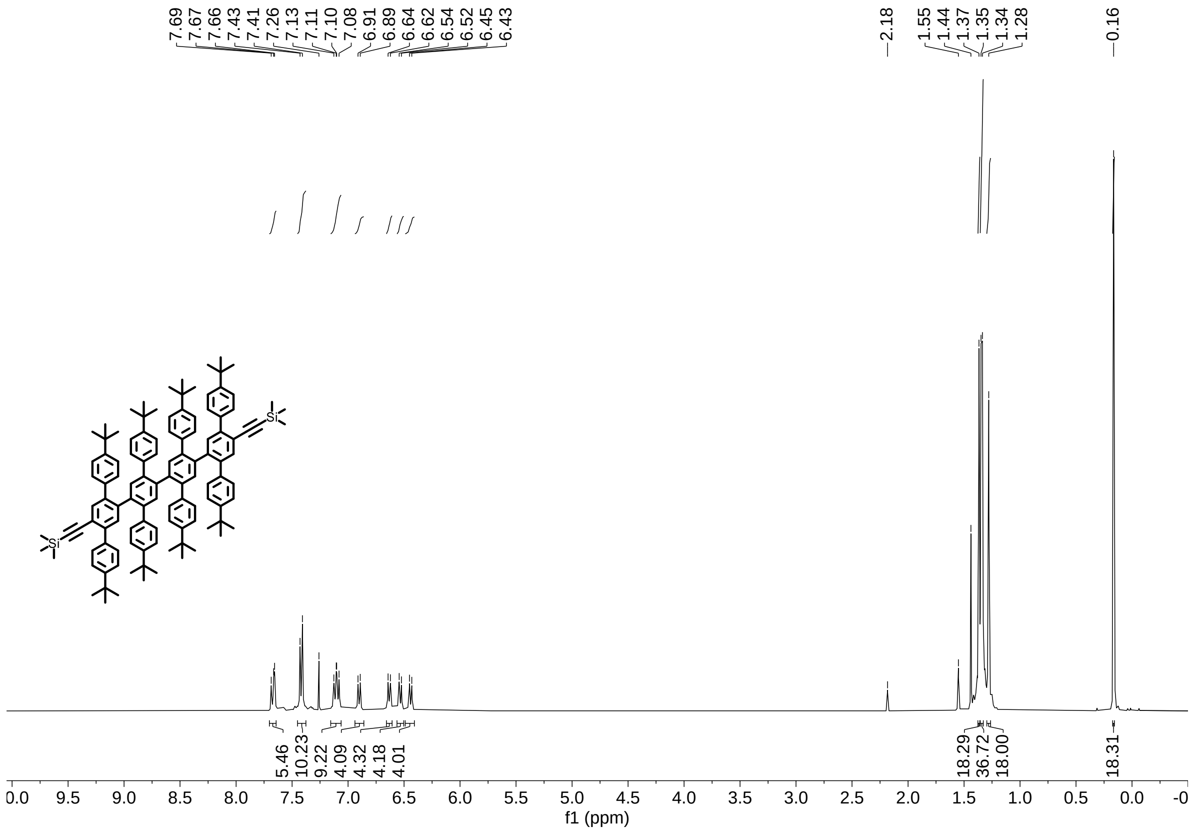

Supplementary Figure 50 | ^{13}H NMR of tetraterphenyl derivative (**20**).

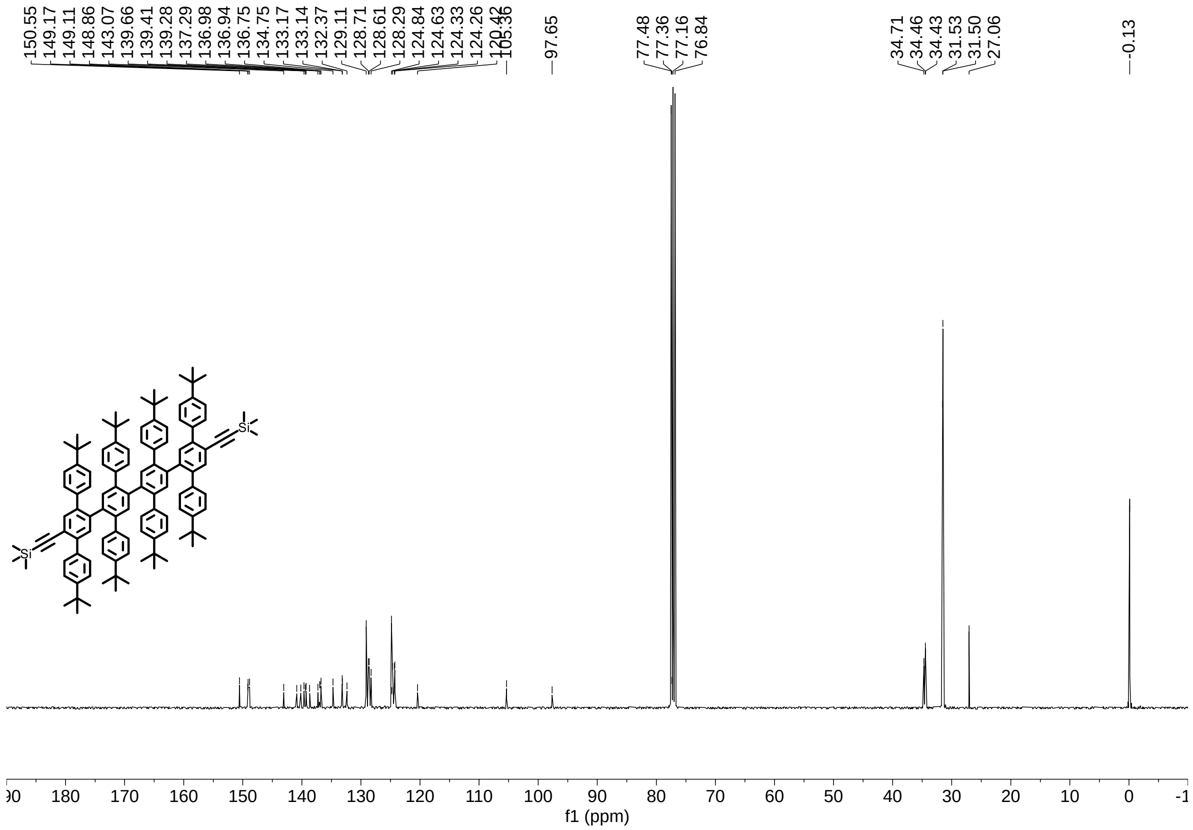

Supplementary Figure 51 | ^{13}C NMR of tetraterphenyl derivative (**20**).

^{1}H and ^{13}C NMR of Dendrimer (4)

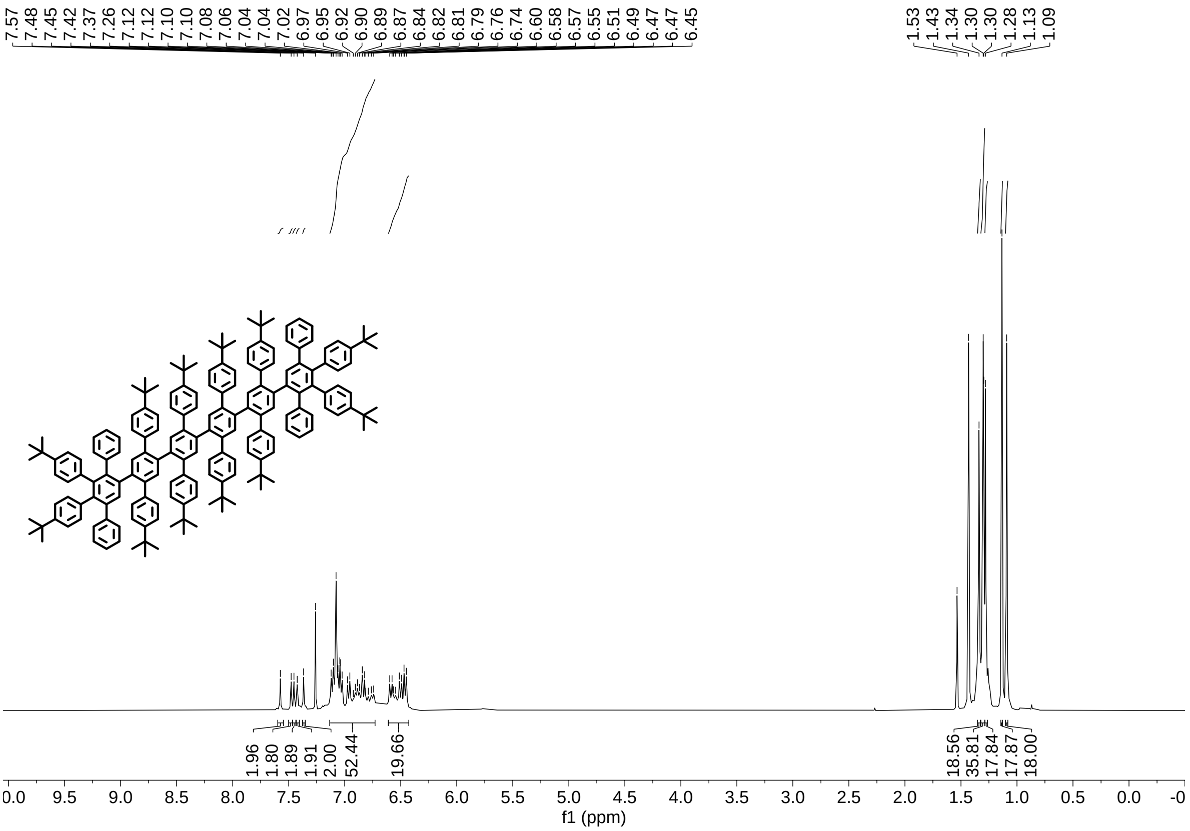

Supplementary Figure 52 | ^{1}H NMR of dendrimer (**4**).

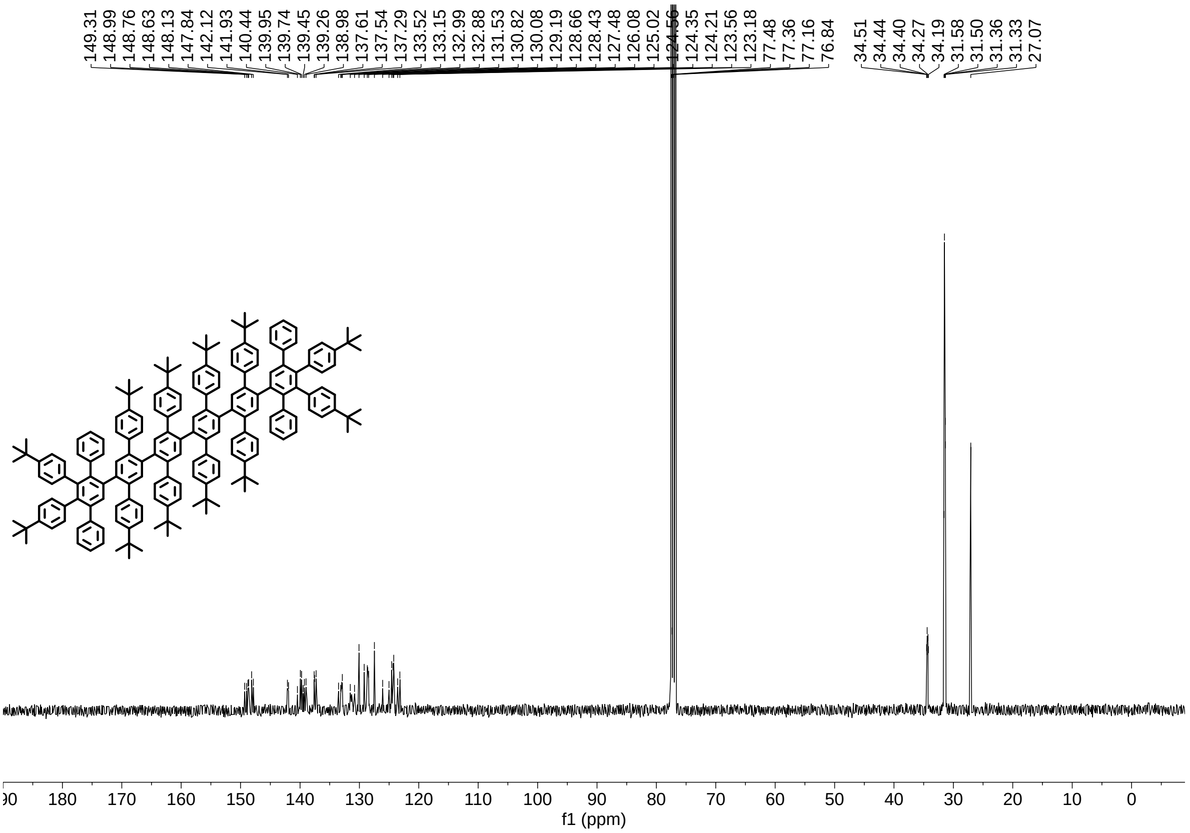

Supplementary Figure 53 | ^{13}C NMR of dendrimer (**4**).

NMR Spectra (600 MHz)

^{1}H NMR spectra of C_{78}-tBu$_6$ in CS_2+THF-d_8 (250 μL + 350 μL) at 298 K and $5.39\cdot10^{-4}$ M

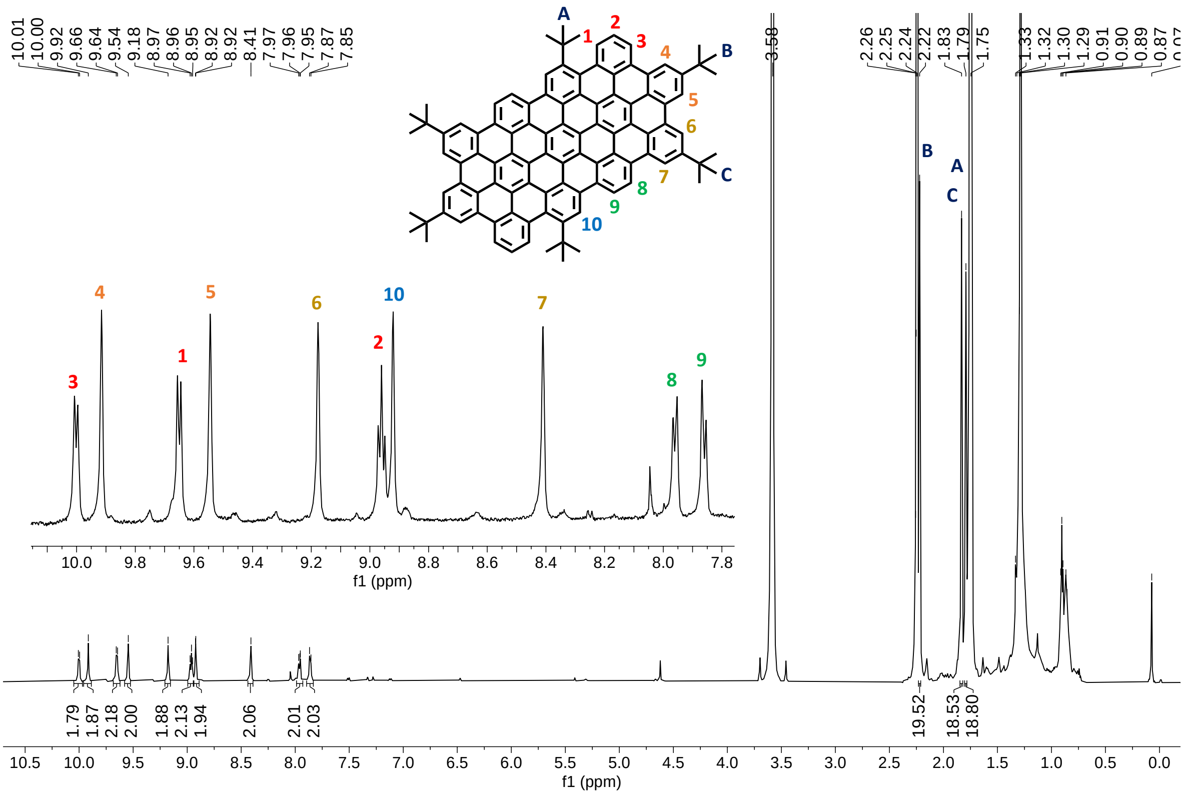

Supplementary Figure 54 | ^{1}H NMR of **C_{78}-tBu$_6$**.

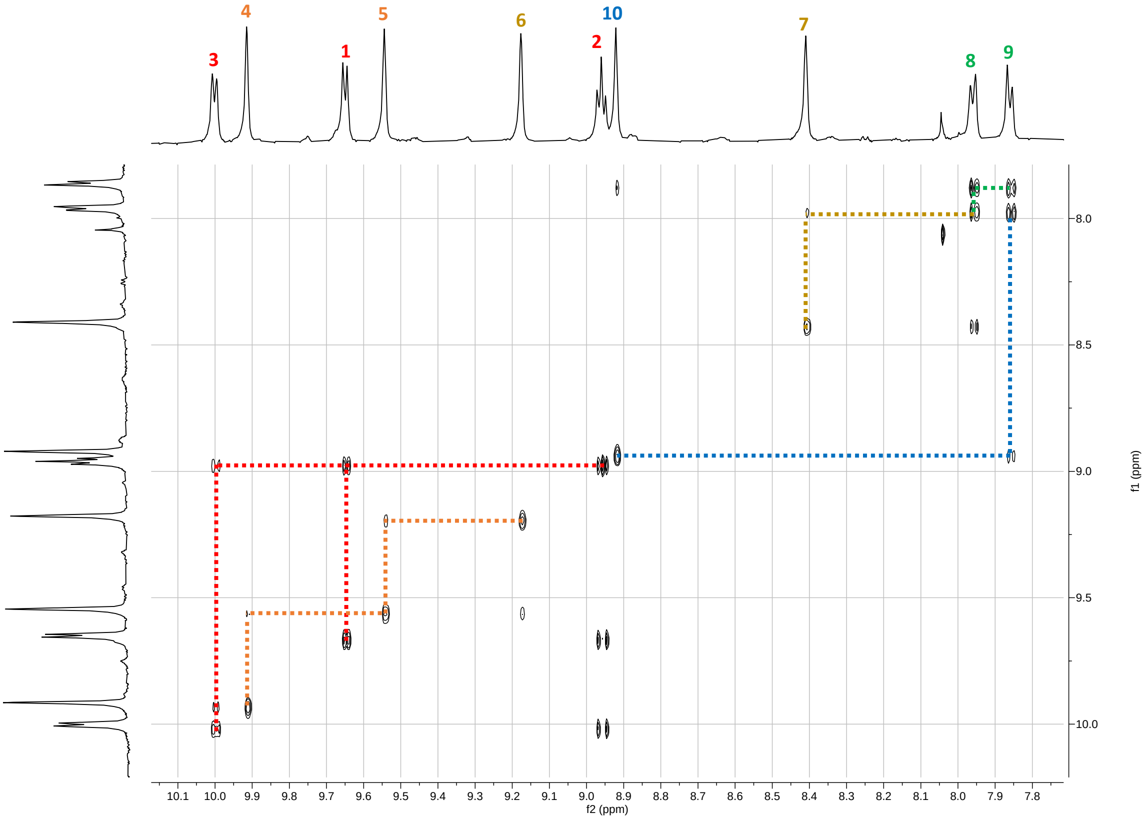

Supplementary Figure 55 | ^{1}H-^{1}H correlation spectroscopy (COSY) of **C_{78}-tBu$_6$**.

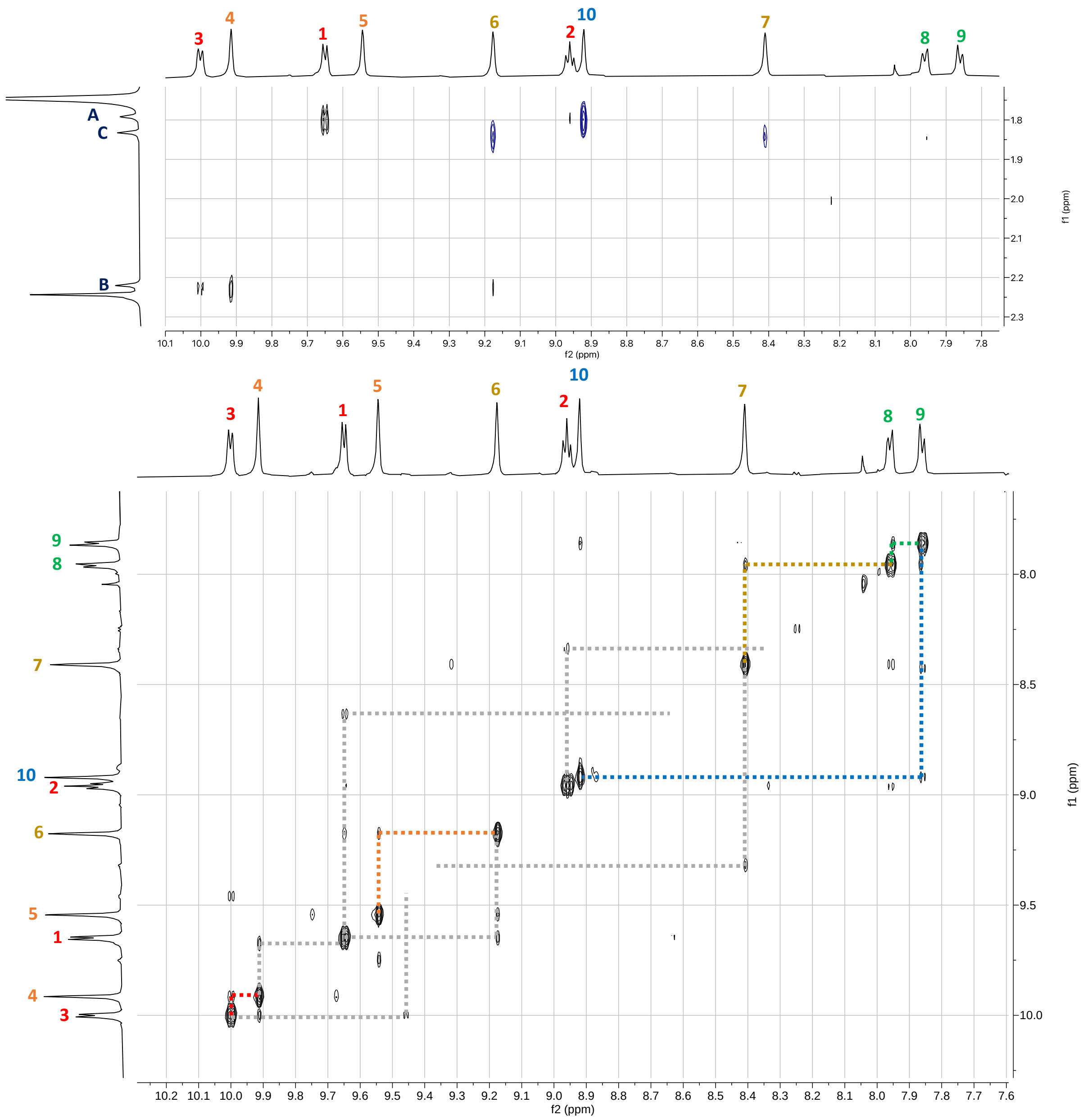

Supplementary Figure 56 | ^{1}H Nuclear Over Hauser Enhancement Spectroscopy (NOESY) of **C_{78}-tBu$_6$.**

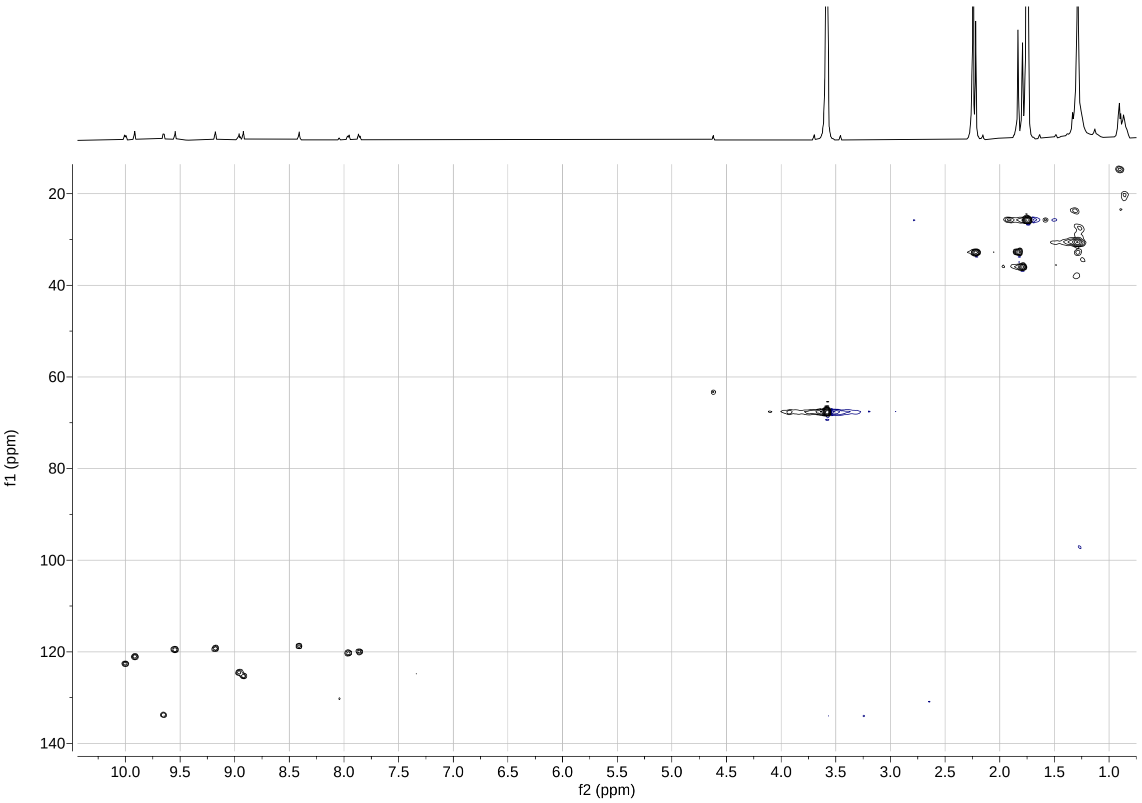

Supplementary Figure 57 | ^{1}H-^{13}C Heteronuclear Single Quantum Coherence (HSQC) spectrum of **C_{78}-tBu$_6$.**

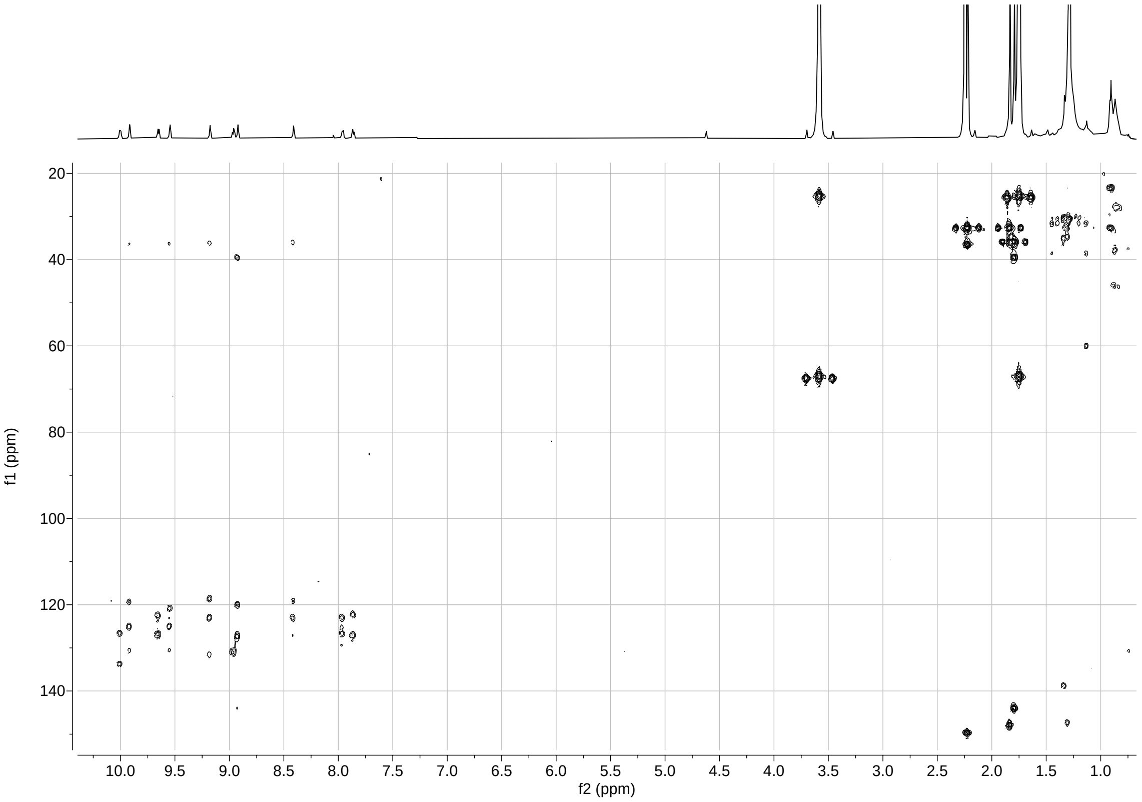

Supplementary Figure 58 | ^{1}H-^{13}C Heteronuclear Multiple Bond Correlation (HMQC) spectrum of **C_{78}-tBu$_6$.**

¹H NMR spectra of C_{96}-tBu$_8$ in CS_2+THF-d$_8$ (250 µL + 350 µL) at 298 K and 5.36·10^{-4} M

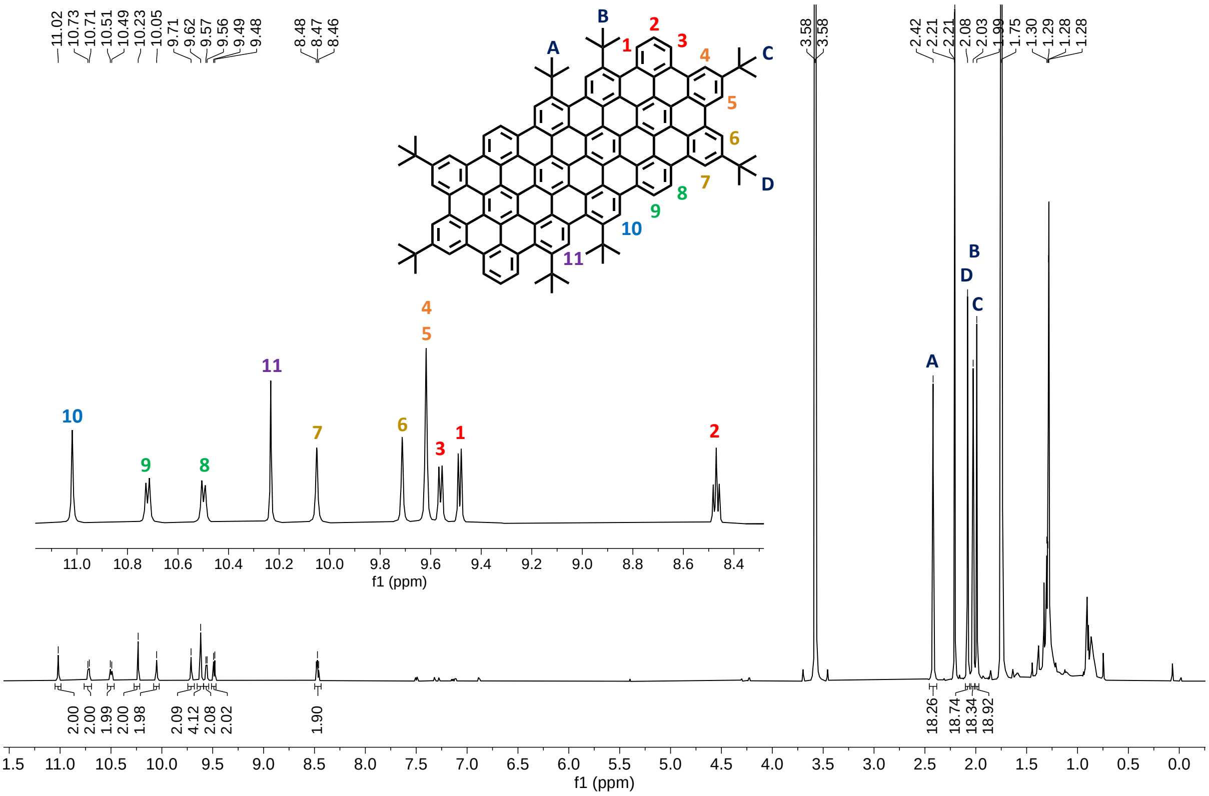

Supplementary Figure 59 | ¹H NMR of **C_{96}-tBu$_8$**.

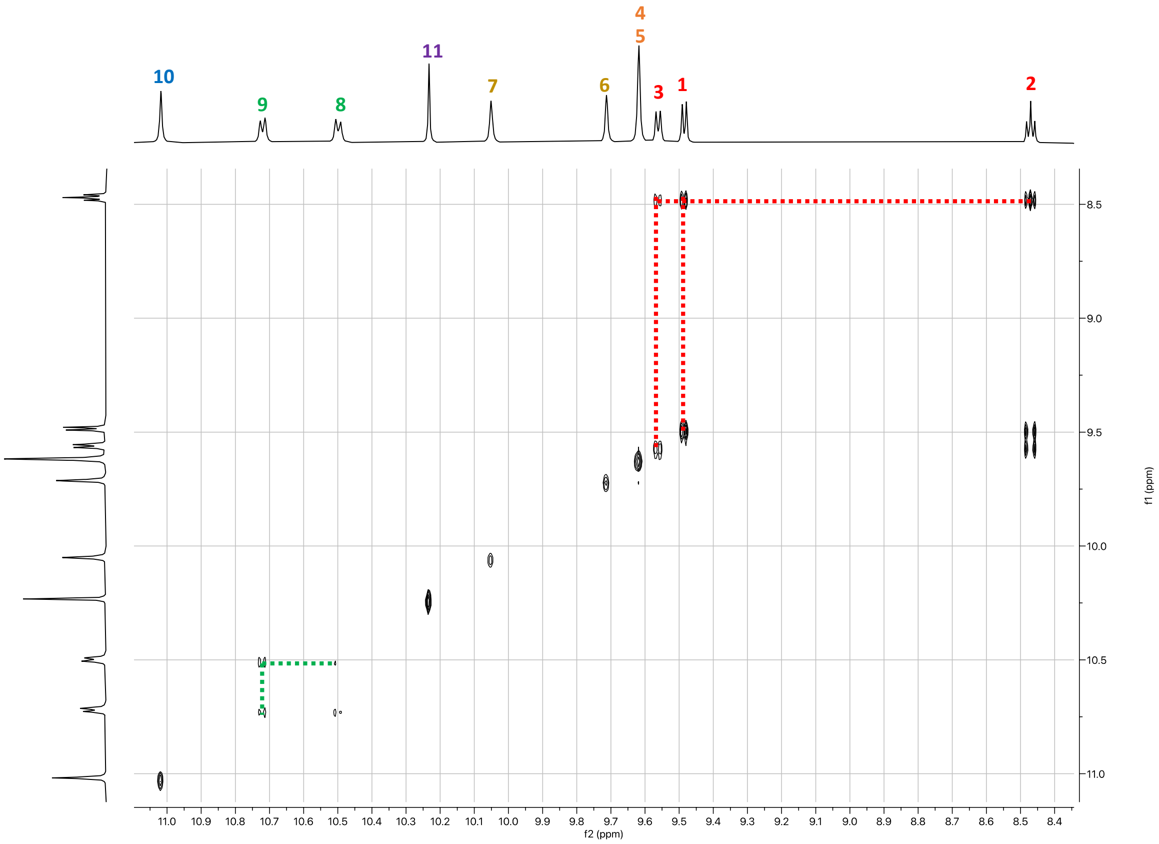

Supplementary Figure 60 | ¹H-¹H correlation spectroscopy (COSY) of **C_{96}-tBu$_8$**.

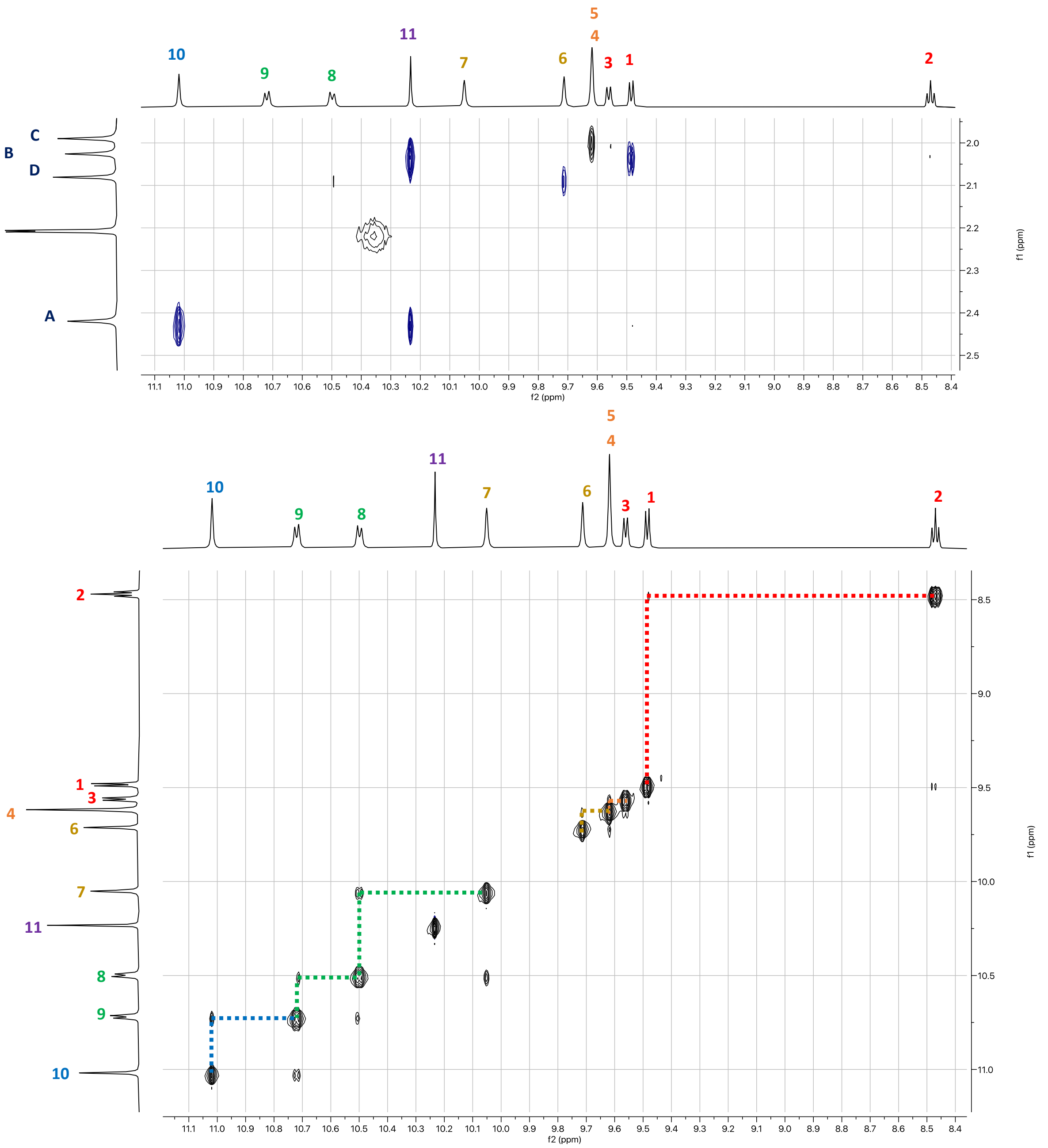

Supplementary Figure 61 | ^{1}H Nuclear Over Hauser Enhancement Spectroscopy (NOESY) of **C_{96}-tBu$_8$**.

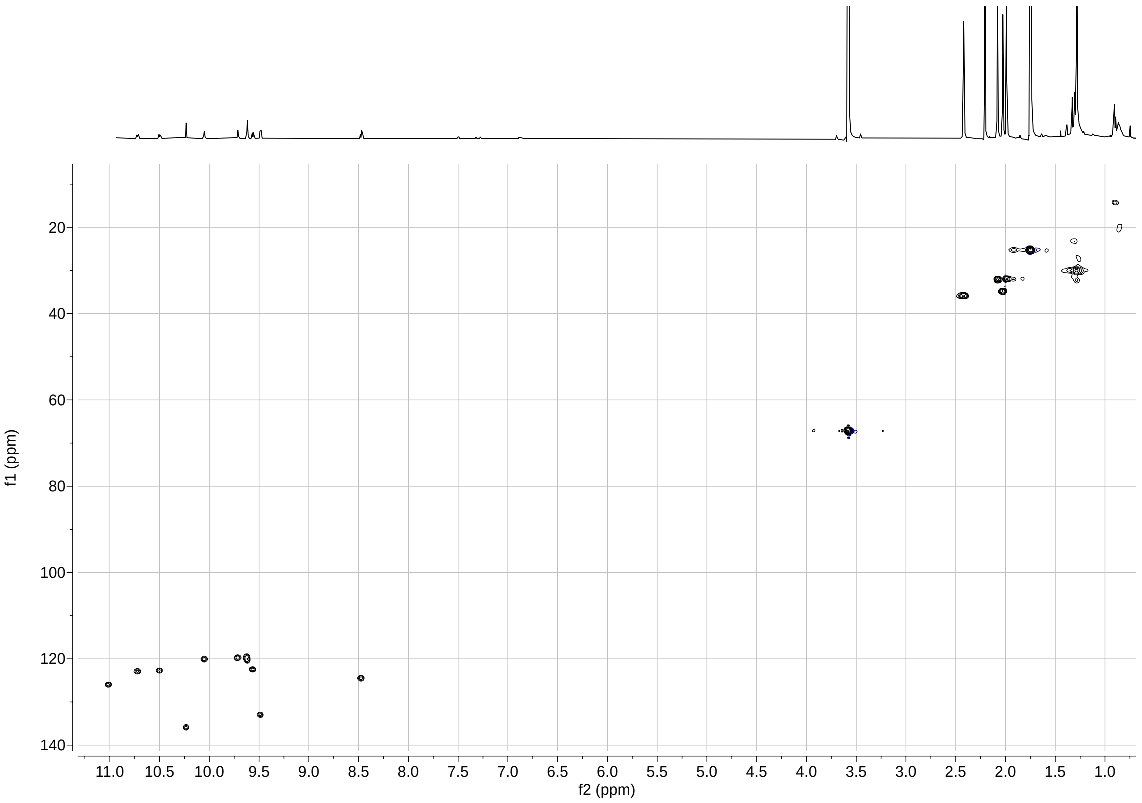

Supplementary Figure 62 | ^{1}H-^{13}C Heteronuclear Single Quantum Coherence (HSQC) spectrum of **C_{96}-tBu$_{8}$**.

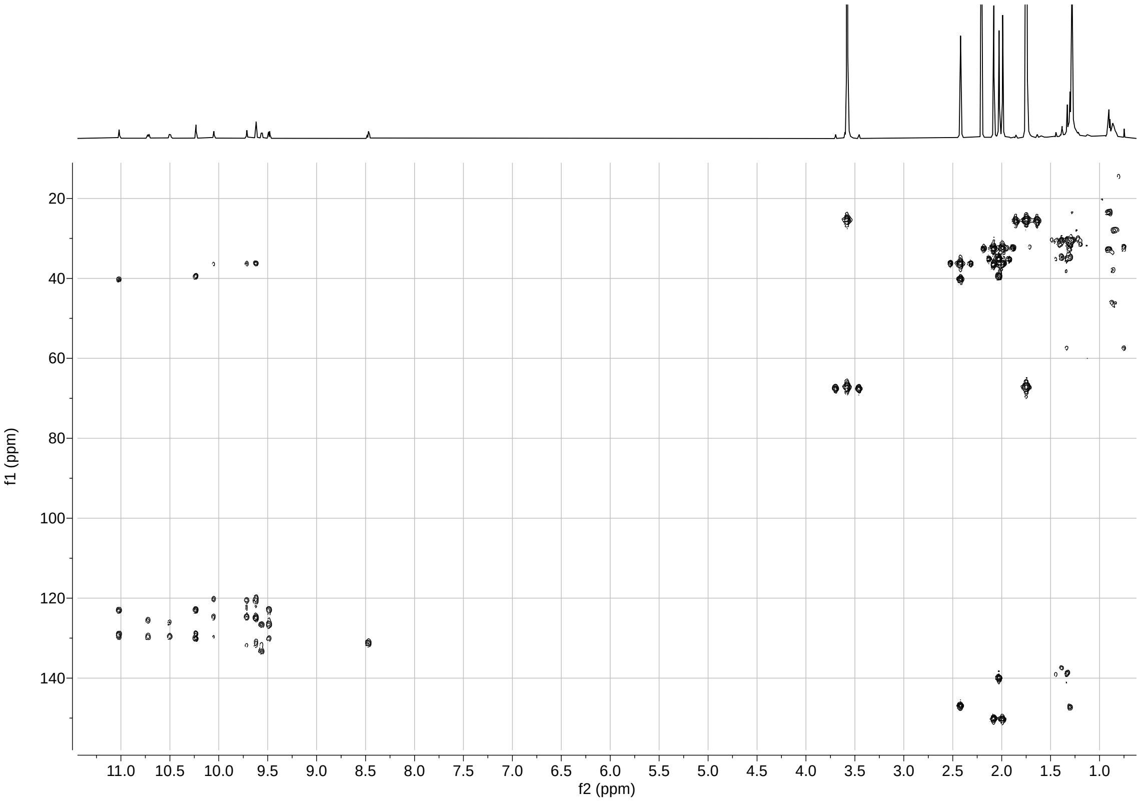

Supplementary Figure 63 | ^{1}H-^{13}C Heteronuclear Multiple Bond Correlation (HMQC) spectrum of **C_{96}-tBu$_{8}$**.

^{1}H NMR spectra of C_{114}-tBu$_{10}$ in CS_2+THF-d_8 (250 μL + 350 μL) at 298 K and $5.86 \cdot 10^{-4}$ M

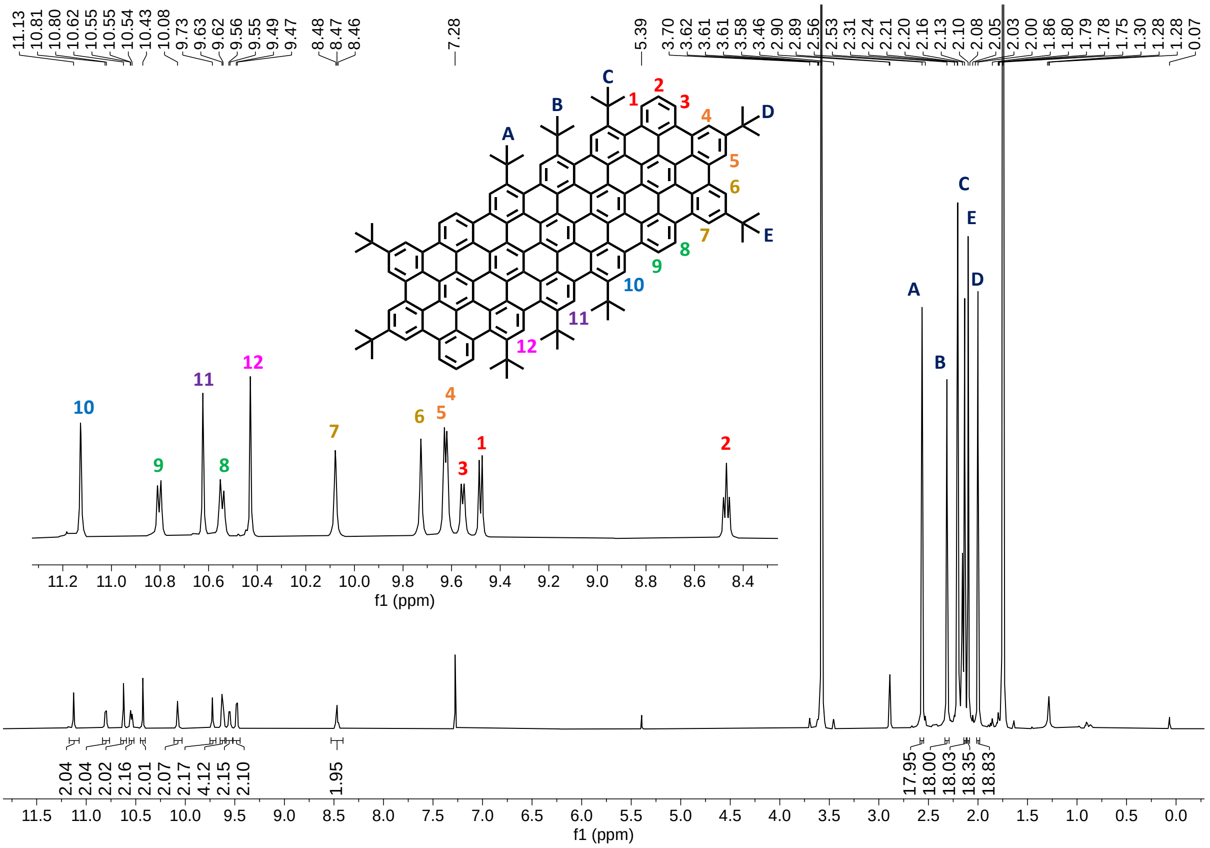

Supplementary Figure 64 | ^{1}H NMR of **C_{114}-tBu$_{10}$**.

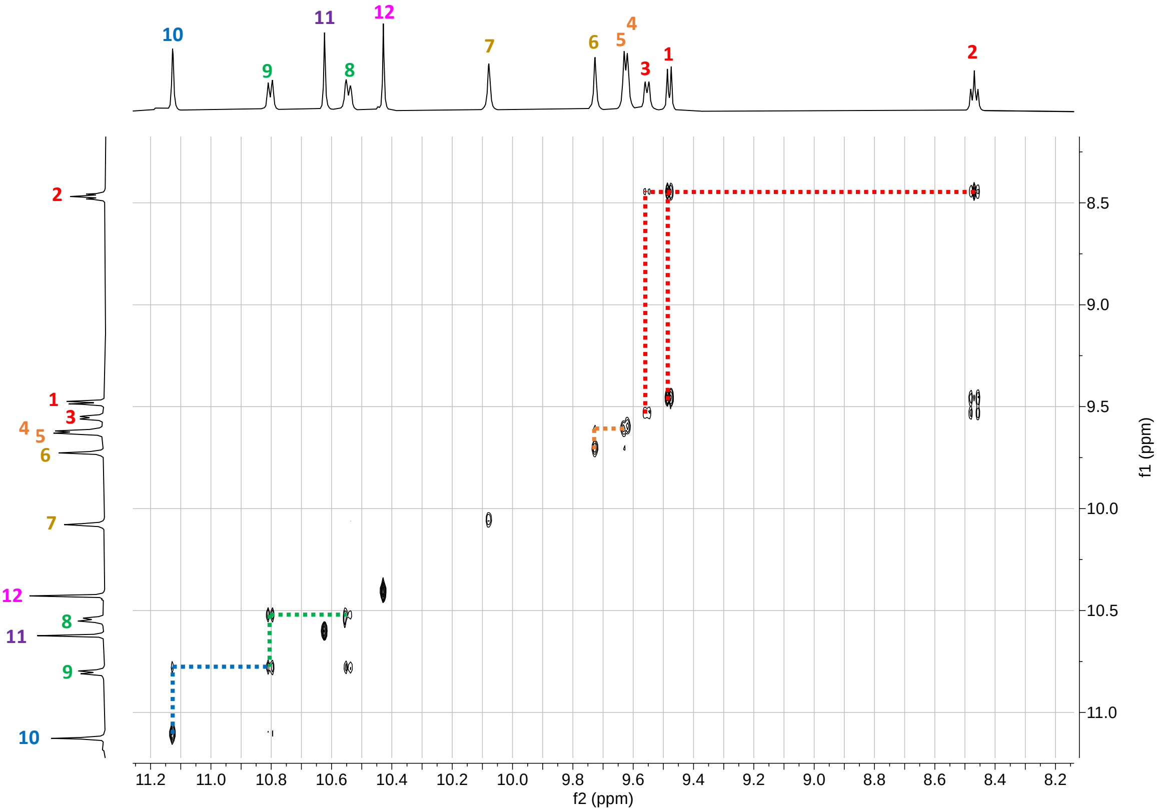

Supplementary Figure 65 | ^{1}H-^{1}H correlation spectroscopy (COSY) of **C_{114}-tBu$_{10}$**.

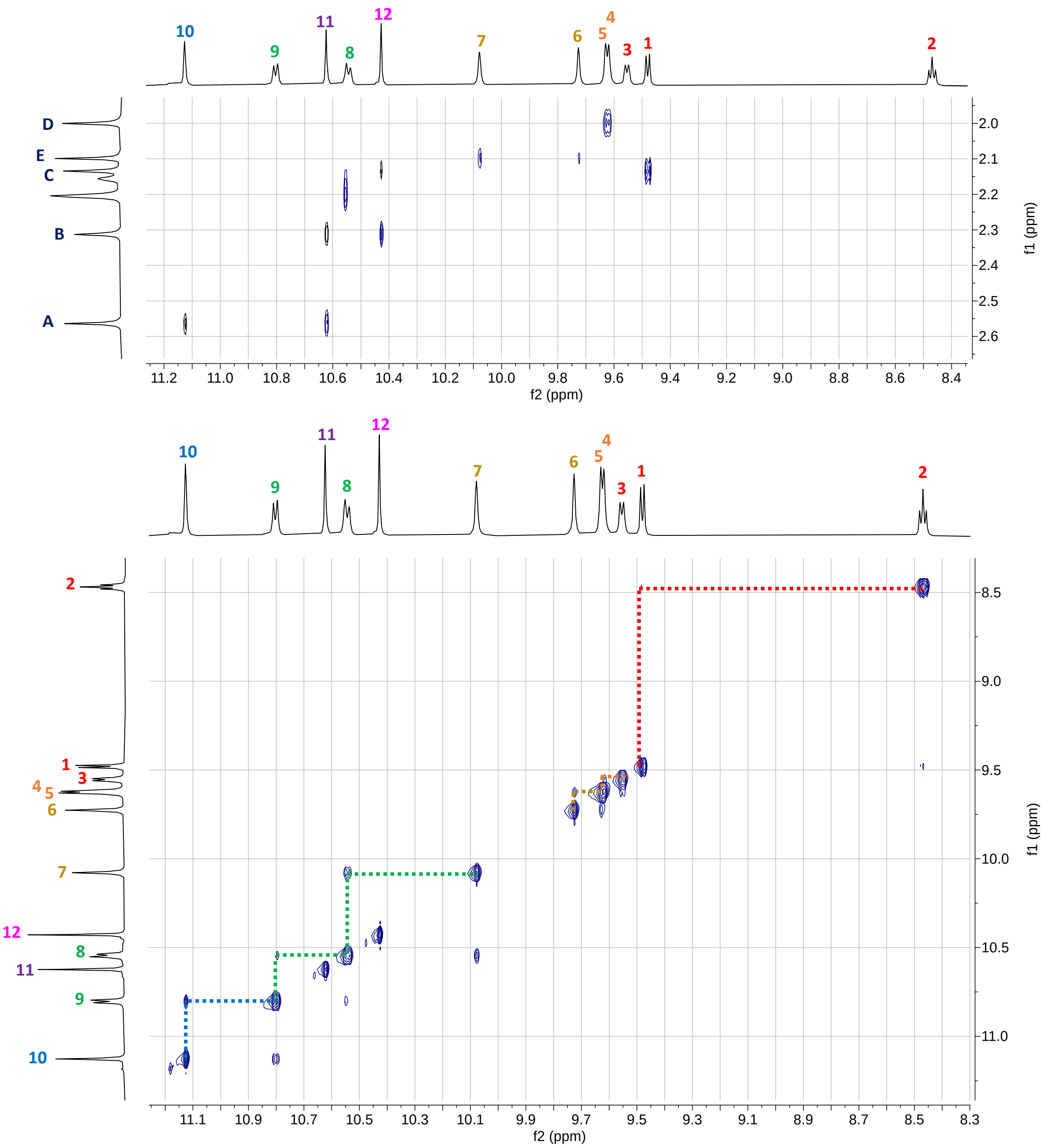

Supplementary Figure 66 | ^{1}H Nuclear Over Hauser Enhancement Spectroscopy (NOESY) of **C_{114}-tBu$_{10}$.**

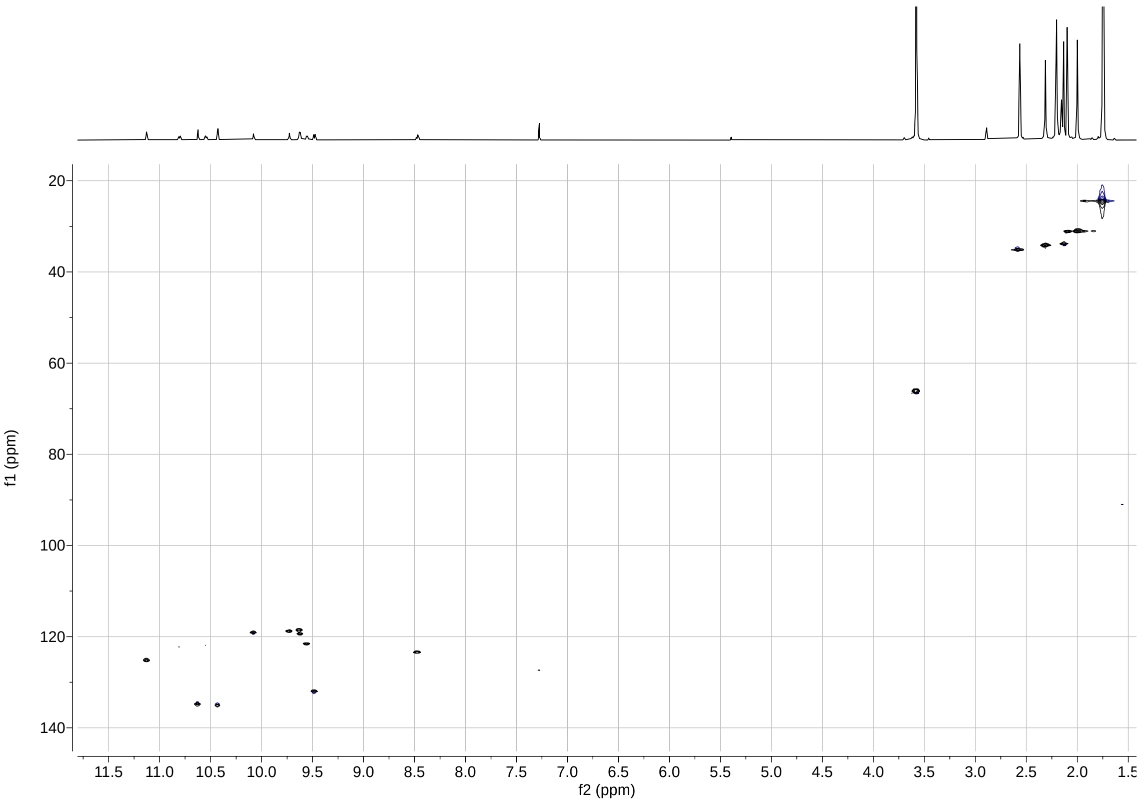

Supplementary Figure 67 | ^{1}H-^{13}C Heteronuclear Single Quantum Coherence (HSQC) spectrum of **C_{114}-tBu$_{10}$**.

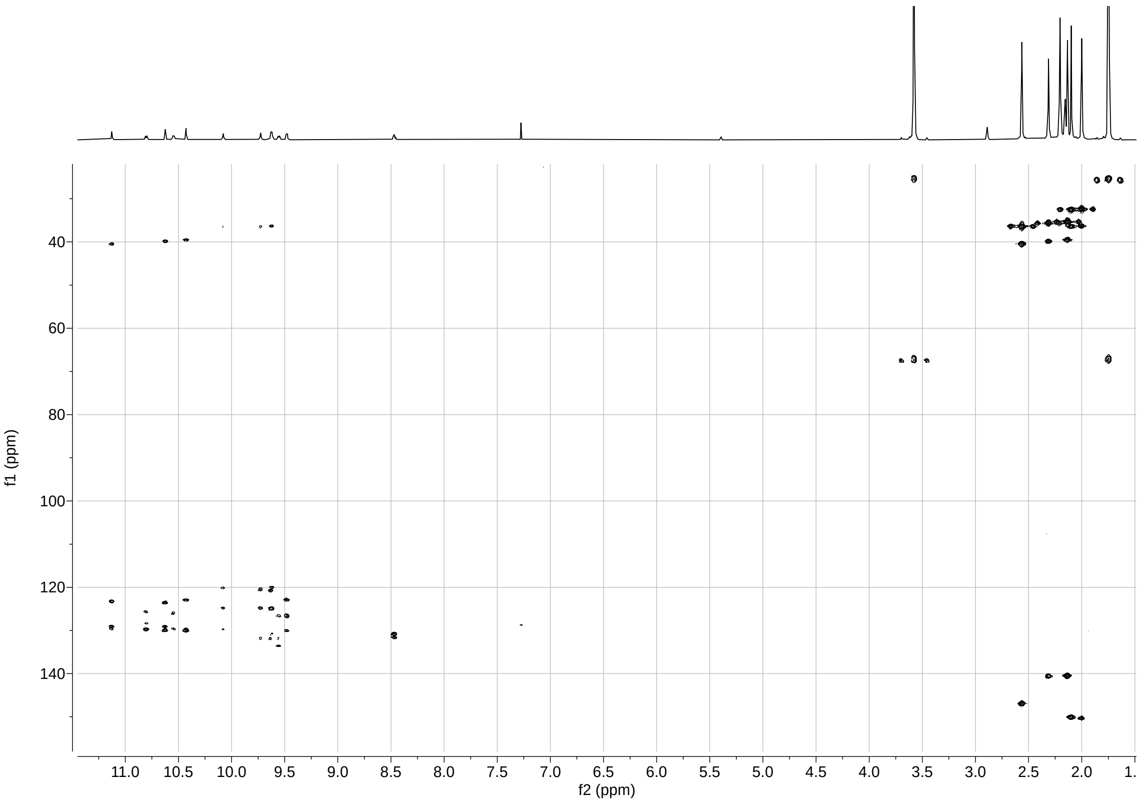

Supplementary Figure 68 | ^{1}H-^{13}C Heteronuclear Multiple Bond Correlation (HMQC) spectrum of **C_{114}-tBu$_{10}$**.

^{1}H NMR spectra of C_{132}-tBu$_{12}$ in CS_2+THF-d$_8$ (250 µL + 350 µL) at 298 K and 5.54·10^{-4} M

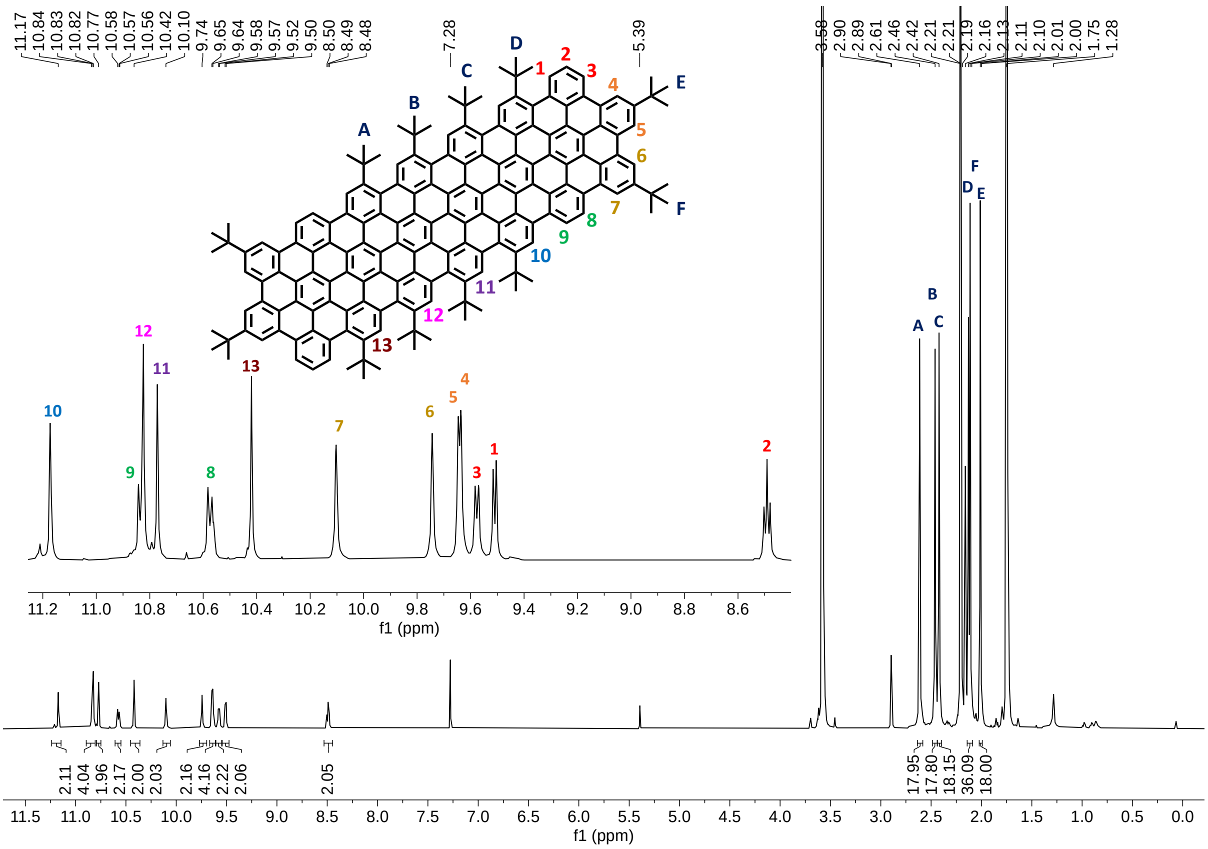

Supplementary Figure 69 | ^{1}H NMR of **C_{132}-tBu$_{12}$**.

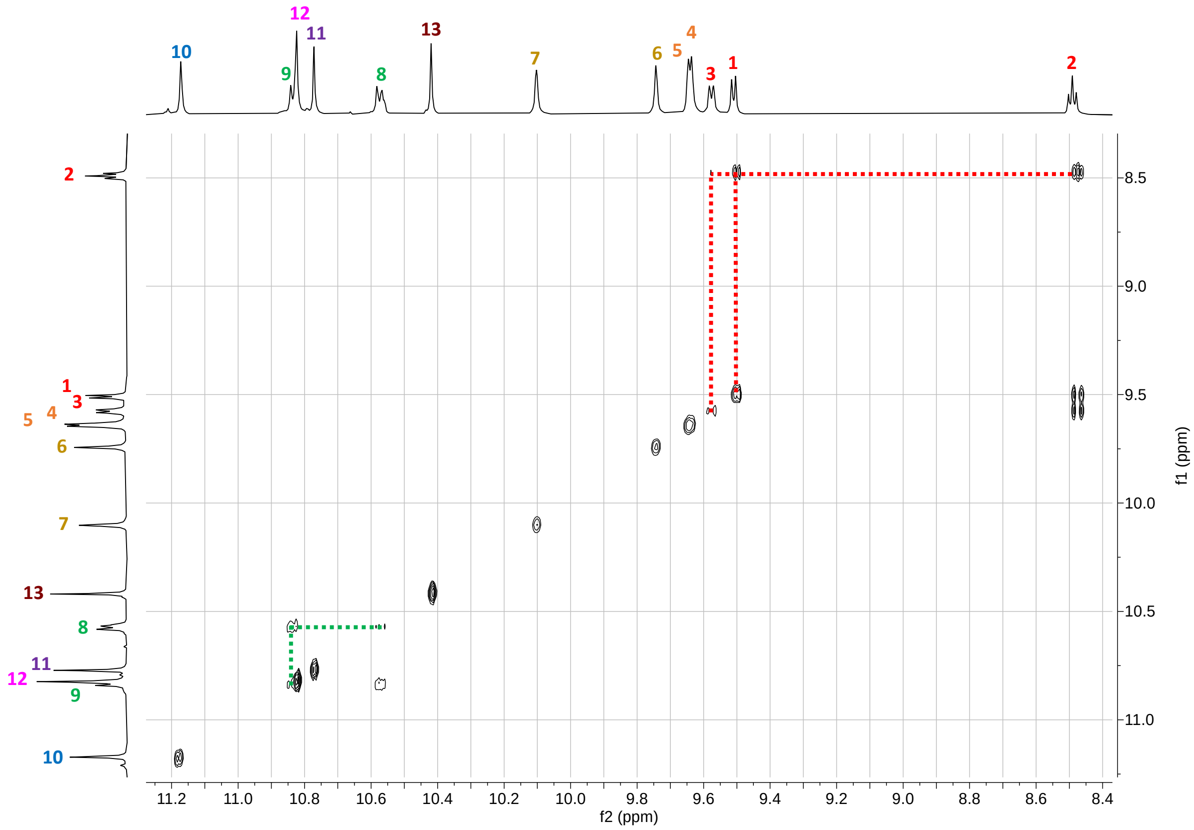

Supplementary Figure 70 | ^{1}H-^{1}H correlation spectroscopy (COSY) of **C_{132}-tBu$_{12}$**.

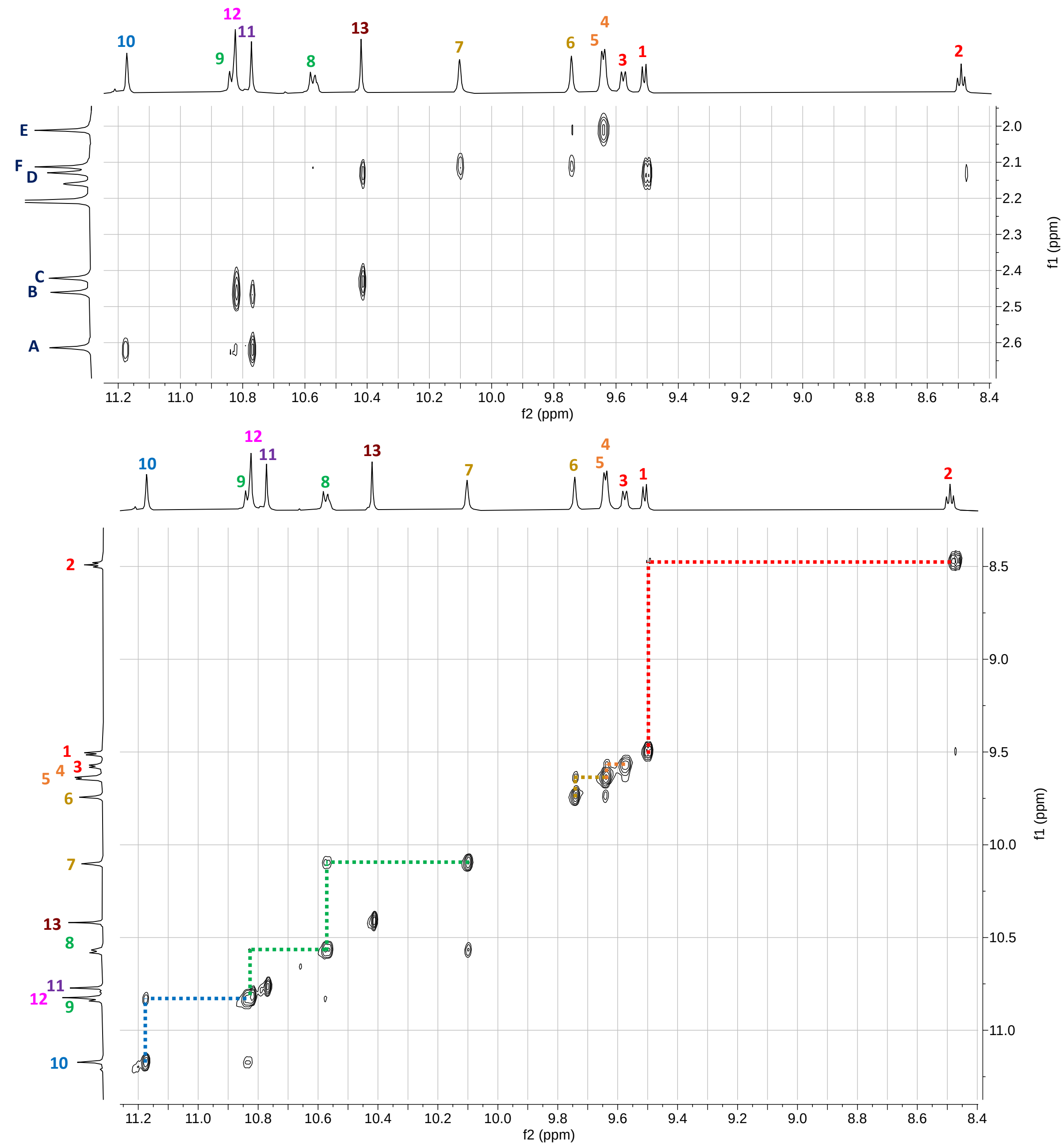

Supplementary Figure 71 | ^{1}H Nuclear Over Hauser Enhancement Spectroscopy (NOESY) of **C_{132}-tBu$_{12}$.**

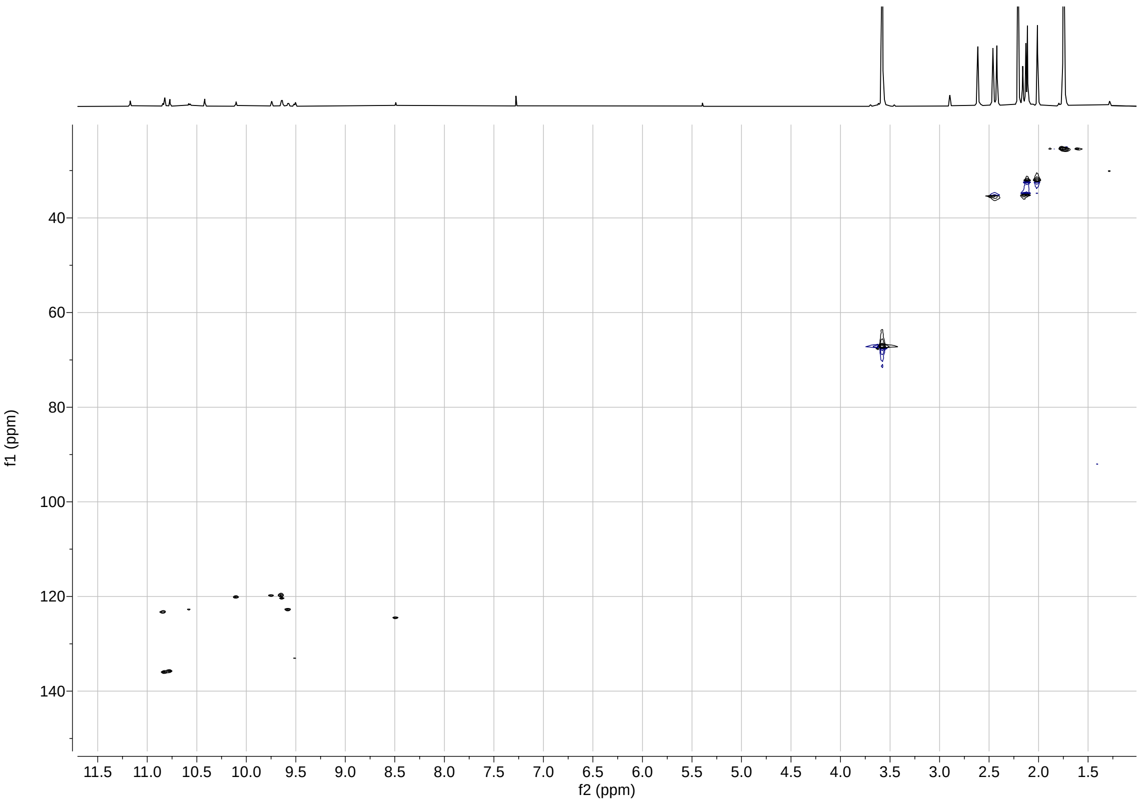

Supplementary Figure 72 | ^{1}H-^{13}C Heteronuclear Single Quantum Coherence (HSQC) spectrum of **C_{132}-tBu$_{12}$**.

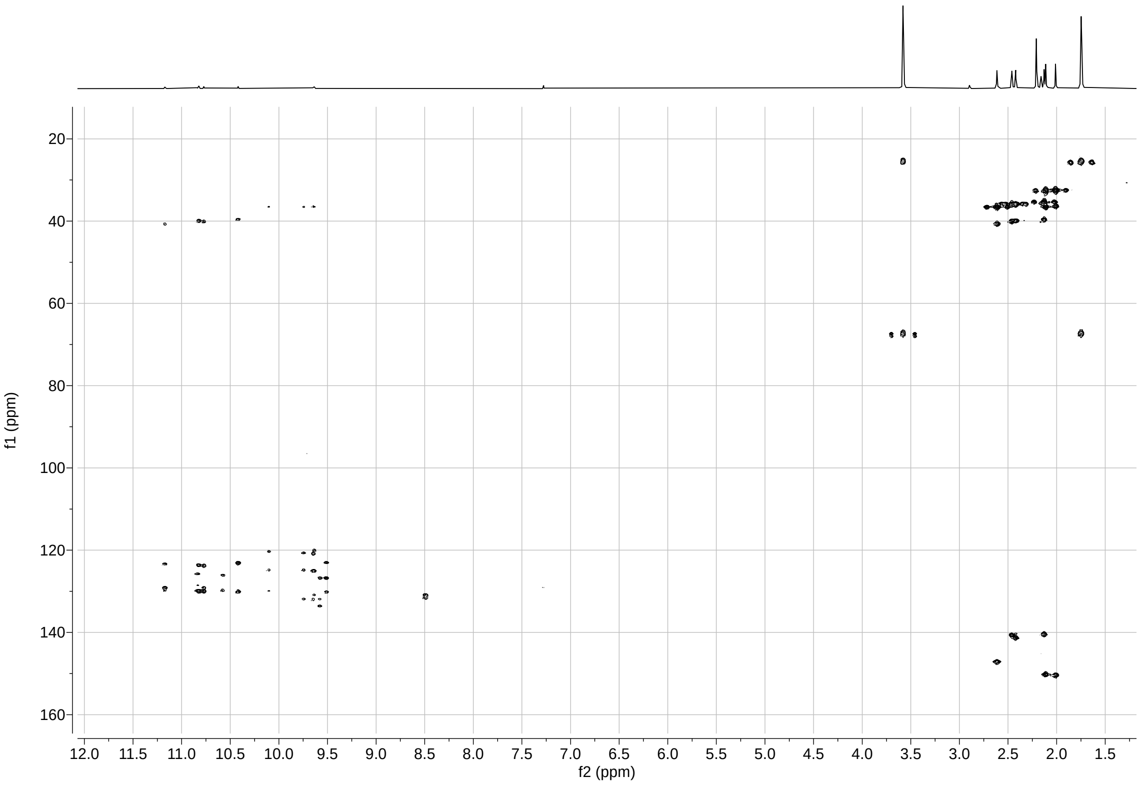

Supplementary Figure 73 | ^{1}H-^{13}C Heteronuclear Multiple Bond Correlation (HMQC) spectrum of **C_{132}-tBu$_{12}$**.

Supplementary references

1. Iyer, V. S., Wehmeier, M., Brand, J. D., Keegstra, M. A. & Müllen, K. From Hexa-peri-hexabenzocoronene to "Superacenes". *Angew. Chem. Int. Ed. Engl.* **36**, 1603-1607 (1997).
2. Thompson, A. P., Aktulga, H. M., Berger, R., Bolintineanu, D. S., Brown, W. M., Crozier, P. S. in 't Veld, P. J. Kohlmeyer, A. Moore, S. G. Nguyen, T. D. Shan, R. Stevens, M. J. Tranchida, J. Trott, C. & Plimpton, S. J. LAMMPS-a flexible simulation tool for particle-based materials modeling at the atomic, meso, and continuum scales. *Comput. Phys. Commun.* **271**, 108171 (2022).
3. Wang, J., Wolf, R. M., Caldwell, J. W., Kollman, P. A., & Case, D. A. Development and testing of a general amber force field. *J. Comput. Chem.* **25**, 1157-1174 (2004).
4. Lu, T., & Chen, F. Multiwfn: A multifunctional wavefunction analyzer. *J. Comput. Chem.* **33**, 580-592 (2012).
5. Gaussian 16, Revision A.03, Frisch, M. J. Trucks, G. W. Schlegel, H. B. Scuseria, G. E. Robb, M. A. Cheeseman, J. R. Scalmani, G. Barone, V. Petersson, G. A. Nakatsuji, H. Li, X. Caricato, M. Marenich, A. V. Bloino, J. Janesko, B. G. Gomperts, R. Mennucci, B. Hratchian, H. P. Ortiz, J. V. Izmaylov, A. F. Sonnenberg, J. L. Williams-Young, D. Ding, F. Lipparini, F. Egidi, F. Goings, J. Peng, B. Petrone, A. Henderson, T. Ranasinghe, D. Zakrzewski, V. G. Gao, J. Rega, N. Zheng, G. Liang, W. Hada, M. Ehara, M. Toyota, K. Fukuda, R. Hasegawa, J. Ishida, M. Nakajima, T. Honda, Y. Kitao, O. Nakai, H. Vreven, T. Throssell, K. Montgomery, J. A., Jr. Peralta, J. E. Ogliaro, F. Bearpark, M. J. Heyd, J. J. Brothers, E. N. Kudin, K. N. Staroverov, V. N. Keith, T. A. Kobayashi, R. Normand, J. Raghavachari, K. Rendell, A. P. Burant, J. C. Iyengar, S. S. Tomasi, J. Cossi, M. Millam, J. M. Klene, M. Adamo, C. Cammi, R. Ochterski, J. W. Martin, R. L. Morokuma, K. Farkas, O. Foresman, J. B. Fox, D. J. Gaussian, Inc., Wallingford CT (2016).
6. Bonomi, M., Branduardi, D., Bussi, G., Camilloni, C., Provasi, D., Raiteri, P. Donadio, D. Marinelli, F. Pietrucci, F. Broglia, R. A. & Parrinello, M. PLUMED: A portable plugin for free-energy calculations with molecular dynamics. *Comput. Phys. Commun.* **180**, 1961-1972 (2009).
